# Three New Stigmatellin Derivatives Reveal Biosynthetic Insights of Its Side Chain Decoration

**DOI:** 10.3390/molecules27144656

**Published:** 2022-07-21

**Authors:** Dorothy A. Okoth, Joachim J. Hug, Ronald Garcia, Rolf Müller

**Affiliations:** 1 Helmholtz-Institute for Pharmaceutical Research Saarland (HIPS), Helmholtz Centre for Infection Research (HZI) and Department of Pharmacy, Saarland University, Campus E8 1, 66123 Saarbrücken, Germany; doroakinyi2@gmail.com (D.A.O.); joachim.hug@helmholtz-hips.de (J.J.H.); ronald.garcia@helmholtz-hips.de (R.G.); 2 German Center for Infection Research (DZIF), Partner Site Hannover-Braunschweig, 38124 Braunschweig, Germany; 3 Helmholtz International Labs, Department of Microbial Natural Products, Saarland University, Campus E8 1, 66123 Saarbrücken, Germany

**Keywords:** stigmatellin, myxobacteria, biosynthesis, natural products, secondary metabolites

## Abstract

Myxobacteria generate natural products with unique chemical structures, which not only feature remarkable biological functions, but also demonstrate unprecedented biosynthetic assembly strategies. The stigmatellins have been previously described as potent inhibitors of the mitochondrial and photosynthetic respiratory chain and originate from an unusual polyketide synthase assembly line. While previous biosynthetic investigations were focused on the formation of the 5,7-dimethoxy-8-hydroxychromone ring, side chain decoration of the hydrophobic alkenyl chain in position 2 was investigated less thoroughly. We report here the full structure elucidation, as well as cytotoxic and antimicrobial activities of three new stigmatellins isolated from the myxobacterium *Vitiosangium cumulatum* MCy10943^T^ with side chain decorations distinct from previously characterized members of this compound family. The hydrophobic alkenyl chain in position 2 of the herein described stigmatellins feature a terminal carboxylic acid group (**1**), a methoxy group at C-12′ (**2**) or a vicinal diol (**3**). These findings provide further implications considering the side chain decoration of these aromatic myxobacterial polyketides and their underlying biosynthesis.

## 1. Introduction

Natural products feature chemical scaffolds, which are considered as privileged structures [1,2] since these molecules are more likely working as high-affinity ligands for more than one type of receptor [3]. An explanation of this frequently observed fact can be given by the evolutionary optimization over millions of years for interactions with biological entities, such as observed during the biosynthesis of natural products which are made via sequential binding of intermediates to different biosynthetic enzymes [4,5]. This optimization procedure results in molecules with multifaceted stereochemistry and polycyclic fused ring structures defining a specific chemical space distinct from synthetic molecules [6]. These unique chemical structures possess often evolutionarily optimized physicochemical properties resulting in diverse biological functions often even with clinical relevance [7].

In order to preserve natural products as a prolific resource for novel drug leads in the near future, it is fundamental to apply distinct concepts and methods and not further exploit the well-known microbial sources [8]. Encouragingly, over the past few decades under-investigated microbes including rare actinomycetes, cyanobacteria, plant endosymbionts, insect pathogenic bacteria and myxobacteria have entered the stage as prolific producers of bioactive natural products [9].

Myxobacteria are rod-shaped Gram-negative δ-proteobacteria, which are phylogenetically united in the order *Myxococcales*. These soil-dwelling microorganisms feature a cooperative “social behavior” based on chemical communication systems and multicellular development stages, resembling the complexity observed in macroscopic organisms [10]. In addition, myxobacteria possess the ability to glide on surfaces and synchronized directional swarming in order to hunt and feed on prey microorganisms cooperatively, via the secretion of enzymes [11]; this behavior is referred as the “wolf-pack strategy” [12].

**Figure 1:**
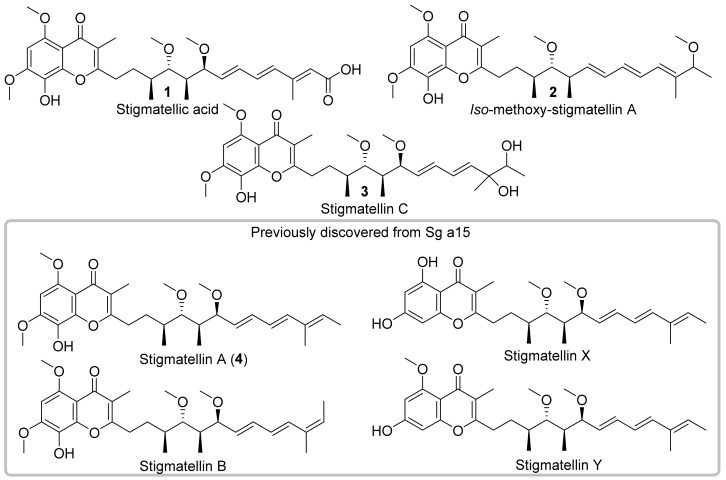
Chemical structures of new stigmatellin derivatives stigmatellic acid (**1**), *iso*-methoxy-stigmatellin A (**2**) and stigmatellin C (**3**) isolated from the myxobacterium *Vitiosangium cumulatum* MCy10943^T^. In contrast to the previously isolated stigmatellin A (**4**) and B (in grey box), these new derivatives show significant differences in the side chains underlining important biosynthetic steps during the biosynthesis of the stigmatellins.

An intriguing example for such exceptional biochemistry is highlighted by the stigmatellin biosynthesis—A polyketide synthase (PKS) derived compound class featuring a 5,7-dimethoxy-8-hydroxychromone aromatic part and a hydrophobic alkenyl chain in position 2. (Figure 1) Stigmatellin A (**4**) and B were originally isolated in 1984 from *Stigmatella aurantiaca* Sg a15 [13,14] and in the same year the mode of action of these antifungal compounds has been elucidated [15]. The ability of stigmatellin to inhibit the cytochrome *bc*_1_ segment of the respiratory chain has been investigated thoroughly in numerous studies [16,17], which also includes a complex structure of stigmatellin bound to cytochrome *bc*_1_ complex from chicken [18]. The total synthesis route to obtain stigmatellin A supported the absolute configuration being (*S*,*S*,*S*,*S*)-stigmatellin A [19].

The biosynthetic origin of the stigmatellins was identified in 1999 [20] and the biosynthesis was later clearly correlated through targeted-gene inactivation revealing the congeners stigmatellin X and Y (Figure 1) [21], of which the later showed potential as an anti-biofilm compound against the *Pseudomonas* quinolone signal system (PQS) [22]. Biosynthetic investigations were focused on the formation of the 5,7-dimethoxy-8-hydroxychromone ring. The chromone ring is probably formed by the action of the cyclization domain encoded in the last biosynthetic module, which replaces the typical thioesterase domain as a release domain. The biosynthesis of the hydrophobic alkenyl chain in position 2 was less investigated, since the formation seemed to be in accordance with the number and arrangement of biosynthetic modules and domains. Nonetheless, the structure of the side chain contains functionality essential for biological activity, since alterations in the side chain such as a shift of a methoxy group, loss of the methyl groups, or saturation of the C=C double bonds drastically affect the binding characteristics of **4** [23]. One of the few studies focusing on the side chain decoration of stigmatellin is presented by in vitro investigations of the *O*-methyltransferases StiD and StiE [24].

We report herein the full structure elucidation as well as cytotoxic and antimicrobial activities of three new stigmatellins, namely stigmatellic acid (**1**), *iso*-methoxy-stigmatellin A (**2**), and stigmatellin C (**3**, isolated as isomers) from the myxobacterium *Vitiosangium cumulatum* MCy10943^T^ and provide further implications considering the unusual biosynthesis and side chain decoration of these aromatic polyketides (Figure 1).

## 2. Results and Discussion

### 2.1. Discovery of Stigmatellic Acid (**1**), Iso-methoxy-stigmatellin A (**2**), and Stigmatellin C (**3**)

**Figure 2:**
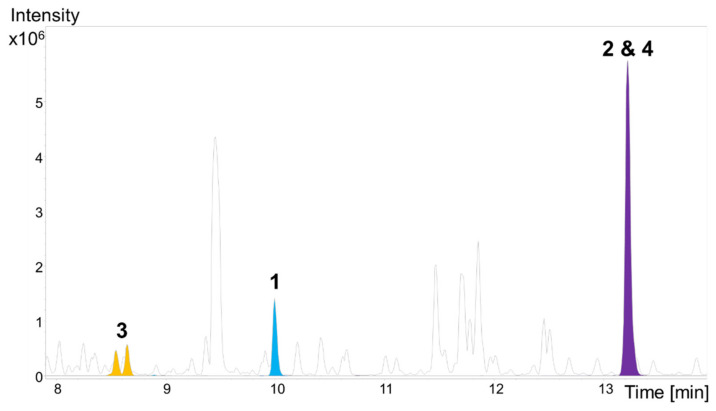
High performance liquid chromatography–mass spectrometry (HPLC–MS) analysis displaying the base peak chromatogram (grey) and extracted ion chromatograms (EICs) of **1** ([M + H]^+^ 545.2755 *m*/*z*, blue), **2** and **4** ([M + H]^+^ 515.3004 *m*/*z*, purple), and **3** ([M + H]^+^ 549.3060 *m*/*z* and 549.3064, orange) from *V. cumulatum* MCy10943^T^ crude extract.

Cultivation of *V. cumulatum* MCy10943^T^ was performed in CYHv3 medium with supplementation of adsorber resin XAD-16. The cell pellet and resin were submitted for further analysis of the associated secondary metabolome via liquid chromatography (LC) coupled with high-resolution mass spectrometry (HRMS), which revealed the uncharacterized secondary metabolites **1**–**3** alongside **4** (Figure 2). According to the observed tandem mass spectrometry (MS^2^) fragmentation pattern of the metabolites **1**–**4**, the compounds **1**–**3** have been identified as new derivatives of the previously described stigmatellin congener **4**. Hence, compound **4** was re-isolated from *V. cumulatum* MCy10943^T^ crude extract and its structure was verified using NMR spectroscopy (Supplementary Materials 1 (Table S1)).

The HRMS of **1** indicated a molecular ion [M + H]^+^ *m*/*z* 545.2755 consistent with a molecular formula of C_30_H_41_O_9_ and the MS^2^ fragmentation of **1** displayed the ion fragments *m*/*z* 513.2973 (C_29_H_37_O_8_^+^), 335.1501 (C_18_H_23_O_6_^+^), 303.1238 (C_17_H_19_O_5_^+^), 263.0924 (C_14_H_15_O_5_^+^), 223.0609 (C_11_H_11_O_5_^+^), and 197.0450 (C_9_H_9_O_5_^+^), consistent with chromone retro-Diels Alder fragmentation, CH_2_-CH_2_ bond cleavage between the chromone moiety and the side chain as well as oxygen mediated β-cleavage (Supplementary Materials 2 (Figures S1–S3)). Compound **1** contains 11 double bond equivalents. The electronic UV/VIS absorption maximum at 266 nm is characteristic for π-π* transition between electrons in the conjugated benzene nucleus and another maximum at 330 nm is attributed to π-π* transition assigned to the pyrone nucleus of a chromone moiety [14,25].

The recorded proton (^1^H) nuclear magnetic resonance (NMR) spectrum indicated one aromatic proton (δ_H_ 6.62 (1H, *s*, H-6), five olefinic protons (δ_H_ 6.71 (1H, *dd*, *J* = 10.45, 15.25, H-9′), 6.31 (1H, *dd*, *J* = 15.3, 10.5 H, H-8′), 6.32 (1H, *d*, *J* = 15.3, H-10′), 5.86 (1H, *dd*, *J* = 7.0, 15.6 H-7′), 5.81 (1H, *s*, H-12′)), four methoxy groups (δ_H_ 3.98 (3H, *s*, H-7-OCH_3_), 3.88 (3H, *s*, H-5-OCH_3_), 3.48 (3H, *s*, H-4′-OCH_3_), 3.24 (3H, *s*, H-6′-OCH_3_)), and two methylene groups (δ_H_ 2.84 (1H, *m,* H-1′) and 2.69 (1H, *m,* H-1′) and 1.92 (1H, *m*, H-2′) and 1.55 (1H, *m,* H-2′)), two oxymethines (δ_H_ 3.94 (1H, *dd*, *J* = 2.5, 7.0, H-6′), 3.11 (1H, *d*, *J* = 2.3, 9.4 Hz)), two methines (δ_H_ 1.71 (1H, *m*, H-3′), 1.69 (1H, *m*, H-5′)) and four methyl groups (δ_H_ 1.98 (3H, *s,* H-9), 2.28 (3H, *d*, *J* = 1.1 Hz, H-16′), 1.15 (3H, *d*, *J* = 6.9, H-14′), 0.72 (3H, *d*, *J* = 7.1, H-15′)).

The observed carbon-13 (^13^C) NMR resonances indicated the presence of nine downfield carbon resonance signals of the chromone (δ_C_ 179.9 (C-4), 165.6 (C-2), 154.0 (C-5), 152.7 (C-7), 148.2 (C-1a), 129.1 (C-8), 117.5 (C-3), 108.4 (C-4a), 93.9 (C-6), six olefinic carbons (δ_C_ 153.4 (C-11′), 138.3 (C-7′), 136.8 (C-10′), 135.2 (C-9′), 133.3 (C-8′), 121.1 (C-12′), four methoxy groups (δ_C_ 62.0 (C-4′-OCH_3_), 57.1 (C-7′-OCH_3_), 57.0 (C-6′-OCH_3_), 56.8 (C-5′-OCH_3_)), two methylene (δ_C_ 30.6 (C-1′) and 28.3 (C-2′)), two oxymethines (δ_C_ 88.5 (C-4′), 82.2 (C-6′)), two methines (δ_C_ 42.7 (C-5′), 35.5 (C-3′)), four methyl groups (δ_C_ 18.4 (C-14′), 14.0 (C-16′), 10.6 (C-15′), 10.1 (C-9)) and an additional carbonyl (δ_C_ 170.8 (C-13′)) for the carboxylic acid. 1D and 2D NMR spectra indicated that the chromone part of **1** was identical to that of **4**, whereas the side chain features different decorations. ^1^H-^13^C heteronuclear multiple bond correlation (spectroscopy) (HMBC) correlations between the aromatic proton H-6 and the carbons at C-1a, C-4a, C-5, C-7 and C-8 and the correlations between the H-9 methyl and the carbons at C-2, C-3 and C-4 exposed the substructure of a chromone ring in **1**. The positions of the C-7 and C-5 methoxy groups were confirmed by their H-7-OCH_3_/C-7 and H-5-OCH_3_/C-5 HMBC correlations. The side chain moiety C-1′ to C-10′ was revealed by the presence of ^1^H-^1^H correlation spectroscopy (COSY) correlations between H-1′/H-2′, H-2′/H-3′, H-3′/H-4′, H-5′/H-15′, H-6′-H-7′, H-7′-H-8′, H-8′/H-9′and H-10′ protons together with H-3′/H-14′-CH_3_ and H-5′/H-15′-CH_3_.

**Figure 3:**
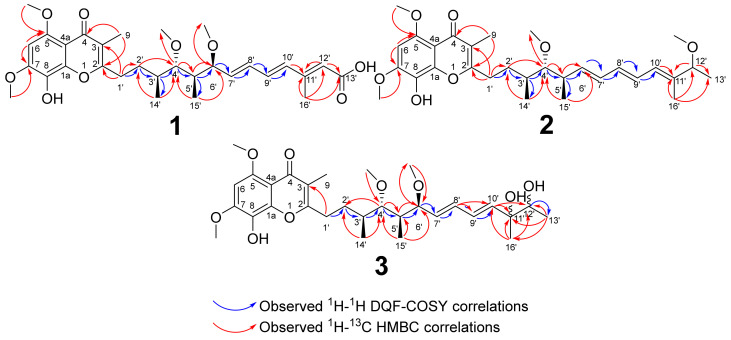
Key ^1^H-^1^H COSY and ^1^H-^13^C HMBC correlations for **1**, **2** and **3**.

Assignment of methoxy groups at the side chain was based on the HMBC cross peaks between 4′-OCH_3_ and 6′-OCH_3_ protons and C-4′ and C-6′ carbons, respectively. Further ^1^H-^1^H COSY correlations between H-16′ methyl (δ_H_ 2.28, d) and H-12′ olefinic proton (δ_H_ 5.81, *s*) in combination with ^1^H-^13^C HMBC correlations between the olefinic proton H-12′ (δ_H_ 5.81, *s*) and the side chain carbons C-10′ (δ_C_ 136.8), C-11′ (δ_C_ 153.4), C-13′ (δ_C_ 170.8), C-16′ (δ_C_ 14.0) and the methyl group H-16′(δ_H_ 2.28, *d*) and the carbons C-10′,C-11′, C-12′ and C-13′ allowed the construction of the -C(CH_3_)=CH-COOH substructure that is attached to the side chain at C-10′. The side chain was connected to the chromone ring at C-2 as evidenced by H-1′/C-2, H-1′/C-3 and H- 2′/C-2 ^1^H-^13^C HMBC cross peaks (Figure 3). Analysis of the 2D NMR data of **1** confirmed it is a stigmatellin derivative showing resonance signals of the chromone moiety together with the polyketide side chain. These COSY and HMBC correlations allowed the assignment of the positions of the three double bonds, the methyl and a terminal carboxylic acid group on the side chain. The large coupling constants (≥15 Hz) of the olefinic protons enabled the stereochemical assignment of the conjugated triene double bonds (C-7′=C-8′, C-9′=C-10′, C-11′=C-12′) to be *E*, *E*, *E*. The relative stereochemistry of C-3′, C-4′, C-5′ and C-6′ were assigned from the observed H-4′ to H-14′ and H-15′ and the H-6′ to H-3′ and H-5′ rotating frame nuclear Overhauser enhancement spectroscopy (ROESY) correlations (Supplementary Materials 3 (Table S2, Figures S4–S9)).

The absolute configuration of **1**–**3** (except C12′ and C13′) was established by in silico comparison and prediction of the ketoreductase domains from Sg a15 and MCy10943^T^ involved in the biosynthetic pathway of **1**–**3** (Supplementary Materials 4 (Tables S3 and S4)). The absolute stereochemistry of stigmatellin A (**4**) had previously been confirmed to be (3*S*, 4*S*, 5*S*, 6*S*, 7*E*, 9*E*, 10*E*) via chemical correlations by employing stereospecific synthetic methods [19,26,27].

Compound **2** with a molecular formula of C_30_H_42_O_7_ observed from HRMS molecular mass of 515.3004 was previously semi-synthetically prepared from stigmatellin A [13]. The observed MS^2^ fragments 335.1501 (C_18_H_23_O_6_^+^), 303.1238 (C_17_H_19_O_5_^+^), 263.0924 (C_14_H_15_O_5_^+^), 223.0609 (C_11_H_11_O_5_^+^), and 197.0450 (C_9_H_9_O_5_^+^) are indicative and characteristic for the stigmatellins and have been observed for all congeners [13,14] (Supplementary Materials 5 (Figure S10)). However, the ion fragments *m*/*z* 483.27 (C_29_H_39_O_6_^+^) and *m*/*z* 451.15 (C_28_H_35_O_5_+) arising from loss of methoxy groups (M + 1-CH_3_OH and M + 1-CH_3_OH-CH_3_OH) were specifically observed from the fragmentation pattern of **2** and **4** (Supplementary Materials 6 (Figures S11–S14)). The NMR spectra were characterized by a chromone ring linked to an alkenyl side chain substituted by two methyl groups as in **4**. In the ^1^H NMR spectrum, the H-8 and H-9 methyl protons of the chromone resonated at δ_H_ 6.68 (1H, *s*) and δ_H_ 1.98 (3H, *s*) while the H-5 and H-7 methoxy resonances were observed at δ_H_ 3.88 and δ_H_ 4.00, respectively. The side chain was characterized by two methylenes (δ_H_ 2.84 (1H, *m*, H-1′), 2.69 (1H, *m*, H-1′) and δ_H_ 2.06 (1H, *m*, H-2′), 1.56 (1H, *m*, H-2′)), two methines (δ_H_ 1.67 (1H, *m*, H-3′) and δ_H_ 2.51 (1H, *m*, H-5′), two oxygenated methines (δ_H_ 2.86 (1H, *m*, H-4′), δ_H_ 3.37 (1H, *s*, H-12′)), four methyl groups (δ_H_ 1.21 (3H, *dd*, H-13′), 1.01 (3H, *d*, H-14′), 1.04 (3H, *d*, H-15′), 1.67 (3H, *d*, H-16′), two methoxy groups δ_H_ 3.45,3H(H-4′), 3.17, 3H (H-12′-OCH_3_) and five olefinic protons δ_H_ 5.69, 1H (H-6′), 6.01, 1H (H-8′) and 6.14,2H (H-7′ and H-9′). From the ^13^C NMR spectrum the chromone signals were δ_C_ 165.5 (C-2), 117.5 (C-3), 179.7 (C-4), 146.1 (C-5), 93.8 (C-6), 152.2 (C-7), 128.9 (C-8), 9.9 (C-9), 148.1 (C-1a), 108.0 (C-4a), 56.7 (C-5-OCH_3_) and 56.9 (7-OCH_3_). ^13^C NMR resonances for the side chain were as follows: C-1′ (δ_C_ 30.6) and C-2′ (δ_C_ 30.4) methylenes, C-3′ (δ_C_ 37.2)and C-5′ (δ_C_ 41.3) methines, C-4′ (δ_C_ 91.6) and C-12′(δ_C_ 83.9) oxygenated methines, C-4′ (δ_C_ 61.7) and C-20′ (δ_C_ 56.1) methoxy groups and C-13′ (δ_C_ 20.3), C-14′ (δ_C_ 16.8), C-15′ (δ_C_ 18.4) and C-16′ (δ_C_ 11.1) methyl groups (Supplementary Materials 7 (Table S5, Figures S15–S20)).

It was noted that the C-6′ (δ_H_ 3.88, δ_C_ 82.4) methoxy group in **4** has shifted to C-12′ (δ_H_ 3.37, δ_C_ 83.9) in **2**. The C-4′ methoxy group (δ_H_ 2.86, δ_C_ 91.6) of **2** deviated from the C-4′ of the other stigmatellins resonating approximately δ_C_ 88.5. The double bond positions at the side chain are also shifted to C-6′, C-8′ and C-10′ instead of the other stigmatellin congeners C-7, C-9′ and C-11′ double bonds. These were confirmed from the ^I^H-^1^H COSY H-4′/H-5′, H-6′/H-7′, H-7′/8′8, H-8′/H-9′,H-9′/H-10′, H-10′/H-11′, H-11′/H-12′, H-12′/H-13′ and ^1^H-^13^C HMBC correlations H-6′/C-C-4′/C-5,C-15′ and H-12′/C-12′-OCH3/C-10′/C-11′, C-13′,C-16′. Höfle et al. suggested that this compound occurred due to proton electrophilic triggered isomerization resulting in the C-6′ methoxy group migrating to C-12′ presumably via heptatrienyl cation intermediate [13]. Due to an overlap of indicative proton signals which are attached to the secondary alcohol of the C-12 hydroxyl group, it was not possible to determine the orientation of the C-12 hydroxyl group in **2**.

The stigmatellin congener **3** was isolated as a mixture of two isomers. Although several attempts were made, it was not possible to separate these isomers. Both isomers seemed to interconvert, and no pure isomer was obtained to allow further stereochemical analyses. The high resolution mass spectrum indicated a molecular ion [M + H]^+^ *m*/*z* 549.3064 and 549.3060 both consistent with a molecular formula of C_30_H_45_O_9_ (Supplementary Materials 8 (Figures S21 and S22)). The MS^2^ fragments of **3** were characterized by *m*/*z* 531.2946 (C_30_H_43_O_8_^+^), 499.2688 (C_29_H_39_O_7_^+^) arising from loss of water (M+H-H_2_O) and methanol (MeOH) (M+H-H_2_O-CH_3_OH) indicative of alcohol and methoxy functional groups presence. In addition, the MS^2^ fragments also showed 335.1501 (C_19_H_23_O_6_^+^), 303.1238 (C_17_H_19_O_5_^+^), 263.0924 (C_14_H_15_O_5_^+^), 223.0609 (C_11_H_11_O_5_^+^), and 197.0450 (C_9_H_9_O_5_^+^), consistent with chromone retro-Diels Alder fragmentation, CH_2_-CH_2_ bond cleavage between the chromone moiety and the side chain and as well as oxygen mediated β-cleavage and α-C-O due to the ester bonds (Supplementary Materials 9 (Figures S23–S25)). Compound **3** contains nine double bond equivalents. The ^1^H NMR spectrum consisted of one aromatic proton (δ_H_ 6.63 (1H, *s*, H-6)) four olefinic protons (δ_H_ 6.31 (1H, *ddd, J* = 3.3, 4.9, 7.2, 15.4, H-11′), 6.20 (1H, *dd*, *J* = 10.5, 15.3, H-8′), 5.81 (1H*, dd, J* = 15.3, H-10′) and 5.60 (1H, *dd*, *J* = 7.4, 15.2, H-7′), four methoxy groups (δ_H_ 4.00 (3H, *s*, 7-OCH_3_), 3.88 (3H, *s*, 5-OCH_3_), 3.47 (3H, *s*, 4′-OCH_3_), 3.22 (3H, *s*, 6′-OCH_3_)), four methines (δ_H_ 3.88 (1H, *dd,* H-6′), 3.11 (1H, *dd*, H-4′), 1.73 (1H, *m*, H-3′), 1.66 (1H, *m,* H-5′)), two methylene groups (δ_H_ 2.84 (1H, *m*, H-1′), 2.70 (1H, *m*, H-1′)) and (δ_H_ 1.88 (1H, *m*, H-2′), 1.55 (1H, *m*, H-2′), five methyl groups δ_H_ 1.98 (3H, *s*, H-9), 1.24 (3H, dd, *J* = 2.1, 2.8, H-16′), 1.14 (3H, *d*, *J* = 6.8, H-14′), 1.10 (3H, *dd*, *J* = 6.5, 10.5, H-13′), 0.73 (3H, *d*, *J* = 7.1, H-15′) and one secondary alcohol proton (δ_H_ 3.56 (1H, *s*, H-12′)).

This was corroborated by ^13^C NMR data, that indicated the olefinic carbons (δ_C_ 138.9/139.1 (C-10′), 133.5 (C-7′), 133.9/133.8 (C-8′), 129.7/129.5(C-11′)) four methoxy groups (δ_C_ 61.7 (C-4′-OCH_3_), 56.9 (C-7-OCH_3_), 56.7 (C-5-OCH_3_), 56.6 (C-6′-OCH_3_), five methyl groups (δ_C_ 23.2 (C-16′), 18.2 (C-14′), 17.8 (C-13′), 10.3 (C-15′), 10.0 (C-9)), one quaternary carbon alcohol (δ_C_ 76.4/76.3 (C-11′), four methines (δ_C_ 88.5 (C-4′), 82.4 (C-6′), 42.9 (C-5′), 35.5 (C-3′)), two methylene groups (δ_C_ 30.2 (C-1′) and 28.3 (C-2′) and a secondary carbon alcohol (δ_C_ 74.9/74.8 (C-12′)).

A comparison of 1D and 2D NMR spectra indicated that **3** comprised a chromone moiety, characterized by downfield carbon signals (δ_C_ 179.7, 165.5, 153.9, 152.6, 148.1, 129.6, 117.3, 108.2, 93.8) similar to that of **4** and stigmatellin B [13], but differed on the structure of the polyketide side chain. The ^1^H NMR spectrum of the side chain of **3** was characterized by four olefinic protons (δ_H_ 6.31 (1H, *ddd, J* = 3.3, 4.9, 7.2, 15.4, H-11′), 6.20 (1H, *dd*, *J* = 10.5, 15.3, H-8′), 5.81 (1H*, dd, J* = 15.3, H-10′) and 5.60 (1H, *dd*, *J* = 7.4, 15.2, H-7′)) and an oxymethine proton (δ_H_ 3.56 (1H, *s*, H-12′)). ^1^H-^1^H gradient COSY (gCOSY) observed between the olefinic proton (δ_H_ 5.60) and methine (δ_H_ 3.88) and δ_H_ olefinic proton 6.20, between olefinic proton (δ_H_ 6.20) and two protons (δ_H_ 5.60) and (δ_H_ 6.31), and between the proton (δ_H_ 6.31) and (δ_H_ 5.82). The oxymethine proton (δ_H_ 3.56) was ^1^H-^1^H gCOSY correlated with the methyl group (δ_H_ 1.13/1.10). ^1^H-^13^C HMBC cross peaks were observed between the terminal methyl group (δ_H_ 1.13/1.10), one quaternary carbon (δ_C_ 76.2/76.0) and a secondary carbon alcohol (δ_C_ 74.8/74.7). The oxymethine proton (δ_H_ 3.56) was HMBC correlated with two methyl groups (δ_C_ 23.2, 18.2/17.8), a quaternary carbon alcohol (δ_C_ 76.2/76.0), and an olefinic carbon (δ_C_ 138.9). The position of the hydroxyl and the double bonds was assigned based on these COSY and HMBC correlations. COSY correlations were observed between the secondary alcohol proton (δ_H_ 3.56) and the terminal methyl group (δ_C_ 17.8). Due to the large coupling constants (*J* = 15) of the olefinic protons, the configurations of the double bonds were assigned to be *E*,*E* (Supplementary Materials 10 (Table S6, Figures S26–S34)).

### 2.2. Bioactivity Testing of **1**–**4**

In general, the stigmatellins **1**–**4** did not feature significant inhibitory activity against Gram-negative or Gram-positive bacterial strains, whereas **4** is moderately active against the yeast *Pichia anomala* and *Candida albicans*, and the fungus *Mucor hiemalis*. It was noted that **4** featured superior antimicrobial activity than the new derivatives **1**–**3** (Table 1). Although all stigmatellins inhibited proliferation of the tested cancer cell lines, **4** displayed higher bioactivity than the congeners **1**–**3** (Table 2).

The modifications in the side chain seen in **1**–**3** might led to decreased antimicrobial and cytotoxic activity. The saturation of the C-11′-C-12′ double bond via oxidation or methoxylation, the different location of the methoxy group or the oxidation of C-13′, led to reduced biological activity of the stigmatellins. This did not seem to be the case for **1** and **3**, which were more polar compared to **4** as evidenced by their retention times on the reverse phase HPLC systems (see Figure 2). Since the congener **2** features similar polarity as its constitutional isomer **4**, the difference in the biological activity originates from the specific chemical structure of **4**.

### 2.3. Biosynthesis of **1**–**3**

**Figure 4:**
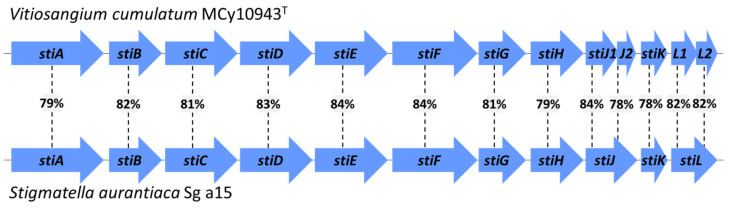
Comparison of the stigmatellin BGC from *V. cumulatum* MCy10943^T^ and *S. aurantiaca* Sg a15. Percent in rectangles highlights identity score in BLAST comparison.

The genetic locus responsible for the formation of stigmatellins in the myxobacterium *S. aurantiaca* Sg a15 alongside the biosynthetic assembly line has been described previously through in silico analysis and gene deletions experiments [20,21]. Therefore, we re-investigated the secondary metabolome of *S. aurantiaca* Sg a15 carefully and could indeed identify the prolific production of **2** and **3**, whereas **1** was only present in minute amounts (Supplementary Materials 11 (Figure S35)). The genome sequence of *V. cumulatum* MCy10943^T^ enabled the identification of the stigmatellin biosynthetic gene cluster (BGC); this BGC clearly resembles the BGC from *S. aurantiaca* Sg a15, with two exceptions in its genetic organization. The gene homolog of *stiJ* within the genome of *V. cumulatum* MCy10943^T^ is divided into the larger gene *stiJ1* (3138 bp) and *stiJ2* (543 bp). Both genes are most likely encoding the last PKS module in the biosynthesis of stigmatellin similar to *stiJ* (3780 bp) from *S. aurantiaca* Sg a15. Similarly, the second deviation can be seen in the genetic organization of *stiL* (Sg a15; 1572 bp) the functionality of which is also encoded by two separate genes *stiL1* (921 bp) and *stiL2* (693 bp) in the genome of *V. cumulatum* MCy10943^T^ (Figure 4, Supplementary Materials 12 (Table S7, Figure S36)). In contrast to that, the surrounding area of the core biosynthetic region features significant differences. The previously identified open reading frames *orf1–8* located upstream of the stigmatellin BGC in the genome of Sg a15 and *orf9* located downstream are not present in the genome of *V. cumulatum* MCy10943^T^ (Supplementary Materials 13 (Figure S37)). These insights further support the previous observation, that *orf8,* which seems to encode a cytochrome P450 enzyme (CYP450), has no function in further decoration of stigmatellin. If these side decorations are catalyzed either by the CYP450 encoded by *stiL* (*stiL*1/2) or by other yet unknown CYP450s remains yet elusive.

**Figure 5:**
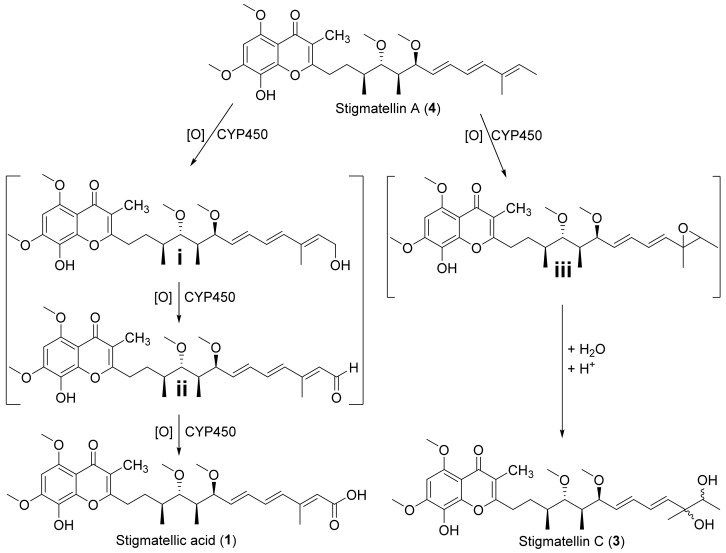
Proposed cytochrome P450 enzyme (CYP450)-catalyzed biosynthetic steps leading to the formation of **1** and **3**. While oxidation of the terminal methyl group of **4** might lead to a hydroxylation cascade with the generation of the intermediates **i** and **ii**, which only differs in their degree of oxidation in comparison to **1**, oxidation of the terminal C=C bond might lead to the formation of the epoxide intermediate **iii** and subsequent hydrolysis forms compound **3**.

Nevertheless, we propose that **1** and **3** are formed from **4** by hydrogenation or epoxidation reactions presumably catalyzed by (different) CYP450s (Figure 5), whereas **2** seems rather to be a direct product from the PKS assembly line without further side chain decoration (Supplementary Materials 12 (Figure S36)). The proposed oxidation of the terminal methyl group of **4**, which leads to a hydroxylation cascade affording **1** via the putative biosynthetic intermediates **i** and **ii**, has been previously described for other natural products. For example the ent-kaurenoic acid oxidase (CYP88A subfamily) catalyzes the formation of gibberellic acid GA_12_ in three steps, starting from ent-kaurenoic acid via the respective hydroxyl and aldehyde intermediate [28]. The formation of the diol side chain presented in **3** via an epoxide intermediate **iii**, parallels the commonly described mechanism for aromatic hydroxylation catalyzed by CYP450s [29]; after the initial epoxidation of the aromatic ring, the newly formed epoxide is opened and re-aromatized via hydride migration to yield a diol [30,31], such as proposed for the biosynthesis of prototenellin-C and proto desmethylbassianin C (*proto* DMB C) [32].

An alternative biosynthetic route leading to **1** and **3** parallels the biosynthetic carboxyl formation in xiamycin A catalyzed by the CYP450 XiaM. XiaM catalyzes a three step hydroxylation cascade to convert a methyl group to a carboxylic acid during xiamycin biosynthesis producing first a hydroxyl intermediate, afterwards a geminal diol, which is transformed to an aldehyde and finally further oxidized to a carboxylic acid [33]. The broad spectrum of stereoselective and stereospecific oxidation of non-activated hydrocarbons catalyzed by CYP450s highlights the difficulty to narrow down if **1** and **3** are formed by the action of one or more tailoring enzymes, since the catalyzed oxidation can be determined by subtle structural changes. For example, the remarkable heterocycle-forming CYP450s AurH in aureothin biosynthesis was engineered by a single-mutation to change its function to regioselectively catalyze the oxidation of a methyl group to a carboxylic acid. In addition, other biosynthetic tailoring enzymes such as peroxygenases could also afford the enzymatic conversion to yield the side chain decorations highlighted by **1** and **3**.

## 3. Materials and Methods

### 3.1. Maintenance of Myxobacterial Cultures

*V. cumulatum* MCy10943^T^, MCy10943^T^, (=DSM 102952^T^=NCCB 100600^T^) a soil isolated species belonging to a novel genus in the suborder *Cystobacterineae* [34] and the producer of sorangiadenosine and 2-hydroxysorangiadenosine [35], was cultivated in 58 L CYHv3 medium [%, (*w*/*v*) 0.2 soytone (BD), 0.3 casitone (BD), 0.2 Glucose (Sigma-Aldrich, St. Louis, MO, USA), 0.8 Soluble starch (Roth), 0.15 Yeast extract (BD), 0.1 CaCl_2_ × 2H_2_O, 0.1 MgSO_4_ × 7H_2_O, 50 mM HEPES, 8 mg/L Fe-EDTA, pH adjusted to 7.2 with 10 N KOH before autoclaving] containing 5% (*v*/*v*) cell inoculum and 2% (*v*/*v*) sterile amberlite resin XAD-16 (Sigma-Aldrich Chemie GmbH, Taufkirchen, Germany) for 10 d at 160 rpm, 30 °C. At the end of fermentation, resin and cells were harvested together by centrifugation at 8000 rpm, for 30 min at 4 °C.

### 3.2. Analysis of Secondary Metabolism of Broth Extracts

The broth extracts were analyzed by high-performance liquid chromatography–high-resolution electrospray ionization-diode array-detector–mass spectrometry (HPLC-HRESI-DAD-MS) on a maXis 4G mass spectrometer (Bruker Daltonics, Billerica, MA, USA) coupled with a Dionex UltiMate 3000 Rapid Separation (RS)LC system (Thermo Fisher Scientific, Waltham, MA, USA) using a BEH C18 column (100 × 2.1 mm, 1.7 μm) (Waters, Eschborn, Germany) with a gradient of 5–95% acetonitrile (ACN) + 0.1% formic acid (FA) in H_2_O + 0.1% FA at 0.6 mL/min and 45 °C over 18 min with ultraviolet (UV) detection by a diode array detector (DAD) at 200–600 nm. Mass spectra were acquired from 150 to 2000 *m*/*z* at 2 Hz. Detection was performed in the positive MS mode, as more secondary metabolites can be expected to ionize in this mode in comparison to negative ion mode [36,37]. The plugin for Chromeleon Xpress (Thermo Fisher Scientific, Waltham, MA, USA, version 6.8) was used for operation of the Dionex UltiMate 3000 RSLC system. HyStar (Bruker Daltonics, Billerica, MA, USA, version 3.2) was used to operate on the maXis 4G mass spectrometer system. HPLC-MS mass spectra were analyzed with DataAnalysis (Bruker Daltonics, Billerica, MA, USA, version 4.2).

### 3.3. Isolation of **1**–**4** by Semi-Preparative HPLC

The extraction, isolation and purification of **1**–**4** from the myxobacterial broth was initiated by chemical extraction via liquid–liquid extraction to concentrate the stigmatellins in the chloroform (CHCl_3_) and ethyl acetate (EA) phase. Subsequent fractionation of these extracts by flash chromatography and further purifications of these resulted in different fractions containing **1**–**4**. Further processing via semi-preparative HPLC yielded pure compound **1**–**4**. Similar compound isolation procedures from myxobacterial broth have been described previously [38,39].

The cell pellet and XAD-16 resin (obtained by centrifugation, see Section 3.1) were extracted by acetone elution and subsequently evaporated under vacuum (6.9 g). The extract was then partitioned between MeOH and *n*-hexane solvents. The MeOH layer was dried under vacuum to yield 5.5 g of extract. This extract was partitioned in water using chloroform (CHCl_3_) and ethyl acetate (EA) to yield 287 mg and 486 mg, respectively, after in vacuo solvent evaporation.

The EA extract (486 mg) was then subjected to flash chromatography on an Isolera™One (Biotage, Uppsala, Sweden) with a SNAP 100 g column packed with reverse phase silica gel (C_18_) (90 Å, 200–400 mesh, 40–63 μm), using H_2_O + 0.1% FA as solvent **A**, MeOH + 0.1% FA as solvent **B** and acetone + 0.1% FA as solvent **C**. The flow rate was 50 mL/min, UV/VIS absorption was set at 270 and 335 nm. Collected fractions (45 mL) were monitored on a Dionex UltiMate 3000 RSLC system (Thermo Fisher Scientific, Waltham, MA, USA) coupled to an amaZon ion trap MS (Bruker Daltonics, Billerica, MA, USA). The elution gradient consisted of an initial isocratic mixture of 95:5 (H_2_O:MeOH) for five column volumes (CVs), then raised to 5:95 (H_2_O:MeOH) for 20 CV. This was followed by another isocratic solvent system to 5:95 (H_2_O:MeOH) for eight CVs. A final gradient of 5:95 (MeOH:acetone) was reached after five CVs. Using high resolution mass spectrometry, three fractions containing compounds of similar masses and identical retention time were pooled together and dried under vacuum; fraction 56–58 (126 mg), fraction 59–62 (300 mg) and fraction 73–76 (425 mg). These fractions were separately purified on an UltiMate 3000 semi-preparative system coupled to a Thermo Scientific Dionex UltiMate 3000 series automated fraction collector (Bruker Daltonics, Billerica, MA, USA) using a C18 Phenomenex Luna (100 Å, 5 µm, 10 × 250 mm) LC column (Phenomenex, Torrance, CA, USA) and eluted with H_2_O + 0.1% FA and ACN + 0.1% FA. The fractions were monitored by mass spectrometry and by using the UV/VIS detector set at 220 nm, 280 nm, 320 nm and 400 nm. The gradient program was set to an initial isocratic gradient of 60:40 (H_2_O:ACN) for five min followed by gradient ramp to 30:70 (H_2_O:ACN) in five min. The gradient was then maintained to 30:70 (H_2_O:ACN) for 18 min before being raised again to 5:95 (H_2_O:ACN) in five min and held for two min before lowering the gradient back to 95:5 (H_2_O:ACN) in one min. The column was re-equilibrated for five min using 95:5 (H_2_O:ACN). The compounds were detected using mass spectrometry on the Agilent 1100 series (Agilent Technologies, Santa Clara, CA, USA) coupled to the HCT 3D ion trap (Bruker Daltonics, Billerica, MA, USA) or with a UV detector on the Dionex UltiMate 3000 RSLC system by UV absorption at 220 nm, 260 nm, 320 nm and 400 nm. The HPLC fractions were dried under N_2_. Compound **1** (10.3 mg) from fraction 59–62 eluted at retention time 14 min, compound **2** (16.5 mg) isolated from fraction 73–76 eluted at retention time 23 min) and compound **3** (21.5 mg) purified from fraction 56–58 was obtained at retention time 17 min). Compound **3** was isolated as a mixture.

The CHCl_3_ extract (287 mg) was chromatographed on an Isolera™One (Biotage, Uppsala, Sweden) with a SNAP 100 g column packed with silica gel (60 Å, 70–230 mesh, 63–200 μm), using *n*-hexane + 0.1% FA as solvent A, EA + 0.1% FA as solvent B, and MeOH + 0.1% FA as solvent C. The mobile phase flow rate was 50 mL/min and UV/VIS absorbance detection was set at 280 and 320 nm. Subsequently after flash chromatographic separation a Dionex UltiMate 3000 RSLC system (as above) coupled to an amaZon ion trap MS (as above) was used to monitor the collected fractions (45 mL aliquots). The gradient elution consisted of an initial isocratic mixture of 95:5 (*n*-hexane:EA) for five CVs, then ramped to 5:95 (*n*-hexane:EA) for 16 CVs. This was followed by another isocratic solvent system 100% (EA) for five CVs. A final gradient of 80:20 (EA:MeOH) was reached after five CVs. Fractions 52–70 contained two compounds of similar masses but different retention times according to LCMS analysis. These fractions were then purified on reverse phase preparative HPLC described above with a slight modification on the gradient elution. The initial gradient was set to 95:5 (H_2_O:ACN) for five min before raising it to 40:60 (H_2_O:ACN) in five minutes. The gradient was again changed to 20:80 (H_2_O:ACN) in 20 min before being raised again to 5:95 (H_2_O:ACN) in five min and held for two min before lowering the gradient back to 95:5 (H_2_O:ACN) in one min. The column was re-equilibrated for five min using 95:5 (H_2_O:ACN). Fraction 52–60 yielded compound **2** (15 mg) at retention time 20.52 min while fraction 61–70 yielded compound **4** (90 mg) at 26.34 min.

Stigmatellic acid (**1**):

Brown amorphous solid; UV λ_max_ 224 nm, 266 nm, 330 nm HRESIMS *m*/*z* 545.2755 [M + H]^+^ (calcd. for C_30_H_41_O_9_, 545.2745, Δ = 1.8 ppm), retention time 10.01 min, [α]^25^_D_ in MeOH = +40.2.

*Iso*-methoxy-stigmatellin A (**2**):

Brown amorphous solid; UV λ_max_ 224 nm, 266 nm, 330 nm HRESIMS *m*/*z* 515.3004 [M + H]^+^ (calcd. for C_30_H_43_O_7_, 515.3003, Δ = 0.2 ppm), retention time 13.03 min, [α]^25^_D_ in MeOH = +36.5.

Stigmatellin C (**3**):

Brown amorphous solid; UV λ_max_ 224 nm, 266 nm, 330 nm, HRESIMS *m*/*z* 549.3062 [M + H]^+^ (calcd. for C_30_H_49_O_9,_ 549.3058, Δ = 0.7 ppm), retention time 8.54 min (isomer 1) and 8.66 min (isomer 2), [α]^25^_D_ in MeOH = +8.9 (mixture of both isomers).

Stigmatellin A (**4**):

Brown amorphous solid; λ_max_ 224 nm, 266 nm, 330 nm HRESIMS *m*/*z* 515.304 [M + H]^+^ (calcd. for C_30_H_43_O_7_, 515.3003, Δ = 0.2 ppm), retention time 13.20 min, [α]^25^_D_ in MeOH = +38.9 (literature values; +37.7 [19] and +38.5 [13]).

### 3.4. NMR Based Structure Elucidation and Chiroptical Measurement

The chemical structure of compounds **1**, **2** and **3** were determined via multidimensional NMR analysis. ^1^H-NMR, ^13^C-NMR and 2D spectra were recorded at 500 MHz (^1^H)/175 MHz (^13^C), conducting an Ascend 500 spectrometer using a cryogenically cooled triple resonance probe (Bruker Biospin, Rheinstetten, Germany). Samples were dissolved in CDCl_3_. Chemical shifts are reported in ppm relative to tetramethylsilane; the solvent was used as the internal standard.

Optical rotation of **1**–**4** were measured in MeOH using the polarimeter model 341 (PerkinElmer Inc., Waltham, MA, USA) in a 50 mm x 2 mm cell at 25 °C ([α]^25^_D_). The sample solution concentration was 2 mg/mL.

### 3.5. Bioactivity Profiling

For evaluation of antibacterial and antifungal activities of compound**s 1**, **2**, **3** and **4** *Escherichia coli* DSM 1116^T^, *E. coli* JW0451-2 (acrB-efflux pump deletion mutant of *E. coli* BW25113), *Pseudomonas aeruginosa* PA14, *Bacillus subtilis* DSM10^T^, *Mycobacterium smegmatis* DSM 43756, *Staphylococcus aureus* Newman, *Candida albicans* DSM 1665, *Citrobacter freundii* DSM 30039^T^, *Wickerhamomyces* *anomalus* DSM 6766 (*Pichia anomala* DSM 6766) and *Acinetobacter baumannii* DSM 30007^T^ strains were assayed using the microbroth dilution assay as described previously [35]. Well-known antimicrobial drugs and negative controls with solvents (MeOH/DMSO) are regularly used to validate the functionality of the performed antimicrobial testing. These strains present a representative selection of bacterial and fungal microorganisms to evaluate the antimicrobial activity of natural products, which has proven its validity in previous myxobacterial studies [40,41,42].

Cytotoxic activity of compounds was determined using HCT-116 (human colon carcinoma cell line, DSMZ No. ACC 581), KB-3-1 (cervix carcinoma cell line, DSMZ No. ACC 158) and U2OS (human bone osterosarcoma epithelial cells) cells seeded at 6 × 10^3^ cells per well of 96-well plates in 180 μL complete medium and treated with test compounds in serial dilution after 2 h equilibration. After five days of incubation, 20 μL of 5 mg/mL MTT (thiazolyl blue tetrazolium bromide) in phosphate-buffered saline (PBS) was added per well and it was further incubated for 2 h at 37 °C. The medium was discarded, and cells were washed with 100 μL PBS before adding 100 μL isopropanol/10 N HCl (250:1) in order to dissolve formazan granules. The absorbance at 570 nm was measured using the microplate reader Infinite^®^ M200Pro (Tecan Group Ltd., Männedorf, Switzerland), and cell viability was expressed as a percentage relative to the respective MeOH control. IC_50_ values were determined by sigmoidal curve fitting. Doxorubicin was regularly used to validate the performance of the cytotoxicity testing. The used human cell lines works as valid indicators to evaluate the cytotoxicity of natural products, which has been proven in previous studies [43].

### 3.6. Applied Software, DNA Sequence Analysis, and Bioinformatics Methods

Genomic DNA isolation and sequencing of *V. cumulatum* MCy10943^T^ has been described previously by Okoth et al. [35]. The *V. cumulatum* MCy10943^T^ genome was screened for secondary metabolite BGCs using the antiSMASH 6.0 [44] online tool and the software Geneious Prime^®^ (Biomatters Ltd., Auckland, New Zealand, 2020.0.5) [45]. The nucleotide or amino acid sequence of interest was aligned with the basic local alignment search tool (BLAST) against our in-house genome database or the publicly available nucleotide database, in order to find homologous genes or proteins. The functional prediction of ORFs was performed by either using protein blast and/or blastx programs and Pfam [46]. To obtain further information concerning the catalytic function of the identified biosynthetic proteins, the amino acid sequences were evaluated by the in silico protein homology analogy recognition engine 2 (Phyre2) [47]. Raw data from the alignments for in silico evaluation of the stigmatellin biosynthetic proteins were stored on the in-house server. Sequence alignments were performed with the embedded Geneious alignment software with the following setups:

Pairwise alignments (alignment type: global alignment with free end gaps; cost matrix: Blosum62; gap open penalty: 12; gap extension penalty: 3). Multiple alignments (alignment type: global alignment with free end gaps; cost matrix: Blosum45; gap open penalty: 12; gap extension penalty: 3; refinement iterations: 2).

The nucleotide sequence of the stigmatellin BGC originating from MCy10943^T^ has been deposited in GenBank and is accessible under the accession number ON210143. The same nucleotide sequence will be implemented in the Minimum Information about a Biosynthetic Gene cluster (MIBiG) database. Further information concerning gene sequences can be found in the Supplementary Information.

## 4. Conclusions

We describe here the discovery, isolation, structure elucidation, biosynthetic, and bioactivities investigations of three new stigmatellins from *V. cumulatum* MCy10943^T^ and highlight the previously unknown side chain decoration of the hydrophobic alkenyl chain of these stigmatellins. These stigmatellins feature either a terminal carboxylic acid group (**1**), a methoxy group at C-12′ (**2**) or a vicinal diol at C-11′/C-12′ (**3**). Genetic in silico analysis of the identified BGC suggest that **1** and **3** are formed from **4** by PKS post assembly hydrogenation or epoxidation reactions, whereas **2** seems to be PKS assembly line product. Although the initial biological evaluation of **1**–**3** revealed disadvantageous antimicrobial and cytotoxic potential in comparison to **4**, these new derivatives might feature another interesting biological function which is currently unknown.

The discovery of the catalytic capability to modify the hydrophobic alkenyl chain of the stigmatellins could also extend the toolbox of catalysts in organic synthesis. The involved biosynthetic proteins could functionalize stereo-, regio-, and chemo-selectively different alkenyl chains, which is often difficult to achieve by non-enzymatic catalysis. The required tailoring reactions to obtain **1** and **3** set the stage for further in-depth biochemical analysis to provide further puzzle pieces to reveal the biosynthesis of these fascinating aromatic myxobacterial electron transport inhibitors. In closing, this study emphasizes the importance of the myxobacterium *V. cumulatum* MCy10943^T^ to discover notable natural products featuring interesting chemical scaffolds and biosynthetic pathways.

## Figures and Tables

**Table 1:** Minimum inhibitory concentration (MIC) values (μg/mL) of stigmatellic acid (**1**), *iso*-methoxy stigmatellin (**2**), stigmatellin C, stigmatellin A (**4**) and different well-known antimicrobial drugs as control (Ctr) against common microbial pathogens.

	MIC Values of 1–4 in μg/mL				
Microorganism	1	2	3	4	Ctr.
*Acinetobacter baumanii* DSM 30007	>128	128	>128	128	1.00 ^a^
*Mucor hiemalis* DSM 2656	64	32	128	16	0.25 ^c^
*Staphylococcus aureus* Newman	128	128	>128	128	1.00 ^b^
*Pseudomonas aeruginosa* PA14 (DSM 19882)	>128	>128	>128	>128	0.13 ^a^
*Escherichia coli acrB* JW0451-2	>128	>128	>128	>128	<0.01 ^a^
*E. coli* wild-type (DSM 1116)	>128	>128	>128	>128	0.03 ^a^
*Bacillus subtilis* DSM 10	>128	>128	>128	128	0.50 ^b^
*Candida albicans* DSM 1665	128	128	128	16	0.25 ^c^
*Pichia anomala* DSM 6766	64	128	128	16	0.25 ^c^
*Citrobacter freundii* DSM 30039	>128	>128	>128	>128	0.03 ^a^

^a^ Ciprofloxacin; ^b^ Vancomycin; ^c^ Amphotericin B.

**Table 2:** Half maximal inhibitory concentrations (IC_50_ values in µg/mL) of stigmatellic acid (**1**), *iso*-methoxy stigmatellin (**2**), stigmatellin C (**3**), stigmatellin A (**4**) and doxorubicin as well-known cytotoxic drug as control (Ctr) against HCT-116 (human colon carcinoma cell line, DSMZ No. ACC 581), KB-3-1 (cervix carcinoma cell line, DSMZ No. ACC 158) and U2OS human bone osteosarcoma epithelial cells.

	IC_50_ Values of 1–4 in μg/mL				
Cancer Cell Line	1	2	3	4	Ctr.
HCT-116	0.35	0.25	1.16	0.09	0.02
KB-3-1	0.95	0.67	3.15	0.14	0.19
U2OS	5.36	3.34	18.80	0.50	0.13

## Data Availability

All data presented in this study are available from the corresponding author on reasonable request.

## Supplementary Materials

The following supporting information can be downloaded at: https://www.mdpi.com/article/10.3390/molecules27144656/s1, Figure S1: UV/VIS and partial ESI+MS spectra of purified stigmatellic acid (**1**); Figure S2: Fragmentation pattern in ESI+MS^2^ experiment of stigmatellic acid (**1**); Figure S3: MS^2^ fragmentation scheme of **1** and proposed structure of identified key ion fragments; Figure S4: ^1^H NMR spectrum of stigmatellic acid (**1**) in methanol-d_4_; Figure S5: ^13^C NMR spectrum of stigmatellic acid (**1**) in methanol-d_4_; Figure S6: HSQC spectrum of stigmatellic acid (**1**) in methanol-d_4_; Figure S7: DQF-COSY spectrum of stigmatellic acid (**1**) in methanol-d_4_; Figure S8: HMBC spectrum of stigmatellic acid (**1**) in methanol-d_4_; Figure S9: ROESY spectrum of stigmatellic acid (**1**) in methanol-d_4_; Figure S10: UV/VIS and partial ESI+MS spectra of purified *iso*-methoxy stigmatellin (**2**); Figure S11: MS^2^ fragmentation scheme of **2** and proposed structure of identified key ion fragments; Figure S12: Fragmentation pattern in ESI+MS^2^ experiment of *iso*-methoxy stigmatellin A (**2**); Figure S13: MS^2^ fragmentation scheme of **4** and proposed structure of identified key ion fragments; Figure S14: Fragmentation pattern in ESI+MS^2^ experiment of stigmatellin A (**4**); Figure S15: ^1^H NMR of *iso*-methoxy stigmatellin A (**2**) in methanol-d_4_; Figure S16: ^13^C NMR of *iso*-methoxy stigmatellin A (**2**) in methanol-d_4_; Figure S17: HSQC spectrum of *iso*-methoxy stigmatellin A (**2**) in methanol-d_4_; Figure S18: DQF-COSY spectrum of *iso*-methoxy stigmatellin A (**2**) in methanol-d_4_; Figure S19: HMBC spectrum of *iso*-methoxy stigmatellin A (**2**) in methanol-d_4_; Figure S20: ROESY spectrum of *iso*-methoxy stigmatellin A (**2**) in methanol-d_4_; Figure S21: UV/VIS and partial ESI+MS spectra of purified stigmatellin C (isomer 1, **3**); Figure S22: UV/VIS and partial ESI+MS spectra of purified stigmatellin C (isomer 2, **3**); Figure S23: MS^2^ fragmentation scheme of **3** and proposed structure of identified key ion fragments; Figure S24: Fragmentation pattern in ESI+MS^2^ experiment of stigmatellin C (isomer 1, **3**); Figure S25: Fragmentation pattern in ESI+MS^2^ experiment of stigmatellin C (isomer 2, **3**) Figure S26: ^1^H NMR spectrum stigmatellin C (**3**) in methanol-d_4_; Figure S27: ^13^C NMR spectrum of stigmatellin C (**3**) in methanol-d_4_; Figure S28: HSQC spectrum of stigmatellin C (**3**) in methanol-d_4_; Figure S29: g-COSY spectrum of stigmatellin C (**3**) in methanol-d_4_; Figure S30: g-COSY spectrum (expanded) of stigmatellin C (**3**) in methanol-d_4_; Figure S31: ^1^H-^13^C *HMBC* spectrum of stigmatellin C (**3**) in methanol-d_4_; Figure S32: ^1^H-^13^C *HMBC* spectrum (expanded) of stigmatellin C (**3**) in methanol-d_4_; Figure S33: HMBC spectrum (expanded) of stigmatellin C (**3**) in methanol-d_4_; Figure S34: ROESY phpr spectrum of stigmatellin C (**3**) in methanol-d_4_; Figure S35: High performance liquid chromatography–mass spectrometry base peak chromatogram (HPLC–MS BPC) (grey) and extracted ion chromatograms from *Stigmatella aurantiaca* Sg a15; Figure S36: Genetic organization of the Stigmatellin BGC from *Vitiosangium cumulatum* MCy10943^T^ and the proposed model of the biosynthesis leading to **1–3**; Figure S37: Dotplot of the entire herein identified stigmatellin BGC from *Vitiosangium cumulatum* MCy10943^T^ (GenBank: ON210143, 87854 bp) and the previously characterized BGC from Sg a15 (MIBiG: BGC0000153, 66808 bp); Table S1: Spectroscopic values for stigmatellin A (**4**) acquired in methanol-d_4_ at 700 MHz; Table S2: Spectroscopic values of stigmatellic acid (**1**) acquired in methanol-d_4_ at 700 MHz; Table S3: Alignment of KR domain sequences from the stigmatellin biosynthesis (MCy10943^T^) and comparison of predicted and observed product stereochemistry; Table S4: Alignment of KR domain sequences from the stigmatellin biosynthesis (Sg a15) and comparison of predicted and observed product stereochemistry; Table S5: Spectroscopic values for *iso*-methoxy stigmatellin (**2**) acquired in methanol-d_4_ at 700 MHz; Table S6: Spectroscopic values for stigmatellin C (**3**) acquired in methanol-d_4_ at 700 MHz; Table S7: Predicted functions of the encoded proteins by the stigmatellin BGC from *Vitiosangium cumulatum* MCy10943^T^. References [48,49] are cited in theSupplementary Materials.

## Sample Availability

Samples of compounds **1**–**4** are available from the authors.
