# Supplementary material for: Three New Stigmatellin Derivatives Reveal Biosynthetic Insights of Its Side Chain Decoration
Source: Molecules. 2022 Jul 21;27(14):4656. doi: 10.3390/molecules27144656 (PMC9317276; doi:10.3390/molecules27144656)
Supplement: Supplementary file 1 [file molecules-27-04656-s001.zip › molecules-1789587-supplementary.pdf]

molecules MDPI

Supplementary Information

Three New Stigmatellin Derivatives Reveal Biosynthetic Insights of its Side Chain Decoration

Dorothy A. Okoth [1,2†], Joachim J. Hug [1,2,3†], Ronald Garcia [1,2,3] and Rolf Müller [1,2,3*]

1 Helmholtz-Institute for Pharmaceutical Research Saarland (HIPS), Helmholtz Centre for Infection Research (HZI) and Department of Pharmacy, Saarland University, Campus E8 1, 66123 Saarbrücken, Germany

2 German Center for Infection Research (DZIF), Partner Site Hannover-Braunschweig, 38124 Braunschweig, Germany

3 Helmholtz International Labs, Department of Microbial Natural Products, Saarland University, Campus E8 1, 66123 Saarbrücken, Germany

* Correspondence: rolf.mueller@helmholtz-hips.de; Tel.: +49-681-98806-3000

† These authors contributed equally to this work.

Table of Contents

1. Results and Discussion 3

Supplementary Material 1 3

Supplementary Material 2 4

Supplementary Material 3 7

Supplementary Material 4 14

Supplementary Material 5 15

Supplementary Material 6 16

Supplementary Material 7 20

Supplementary Material 8 27

Supplementary Material 9 28

Supplementary Material 10 31

Supplementary Material 11 41

Supplementary Material 12 42

Supplementary Material 13 44

2. References 45

molecules MDPI

1. Results and Discussion

Supplementary Materials 1

Table S1. Spectroscopic values for stigmatellin A (**4**) acquired in methanol-d_4 at 700 MHz.

Stigmatellin A (**4**)			
**Position**	**δC**	**δH,** [m, *J* (Hz)]	**δC,** type [1]
1a	148.0	-	148.0
2	165.4	-	165.4
3	117.3	-	117,3
4	179.7	-	179.6
4a	108.2	-	108.7
5	153.8	-	154.0
6	93.7	6.59 s 1H	93.8
7	152.5	-	152.6
8	128.9	-	129.1
9	10.0	1.97 s 3H	9.9
1’	30.5	2.83 m 1H<br>2.67 m 1H	30.6
2’	28.2	1.92 m 1H<br>1.56 m 1H	28.5
3’	35.5	1.73 m 1H	35.6
4’	88.5	3.20 m 3H	88.8
5’	42.9	1.66 m 1H	43.0
6’	82.4	3.88 m 3H	82.7
7’	132.5	5.57 dd 1H (7.7)	132.5
8’	134.7	6.20 m 1H	134.6
9’	126.4	6.20 m 1H	126.3
10’	138.9	6.20 m 1H	138.9
11’	135.9	-	135.9
12’	128.5	5.57 q 1H (7.7)	128.4
13’	14.0	1.75 s 3H	13.9
14’	18.3	1.14 dd 3H (3.05/6.7)	18.3
15’	10.5	0.74 d 3H (7.0)	10.7
16’	12.1	1.75 s 3H	12.0
17’	56.6	3.87 s 3H	56.8
18’	56.9	3.97 s 3H	57.0
19’	61.7	3.46 s 3H	61.6
20’	56.5	3.20 s 3H	56.5

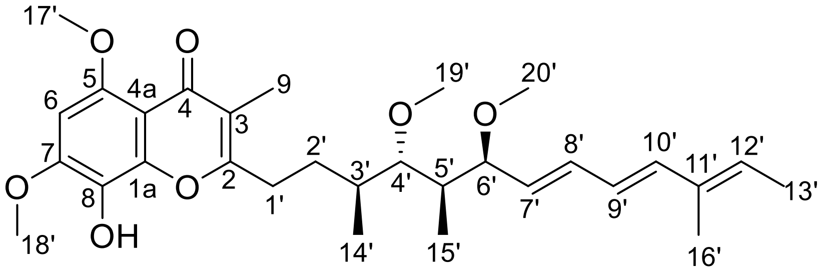

Stigmatellin A

Supplementary Materials 2

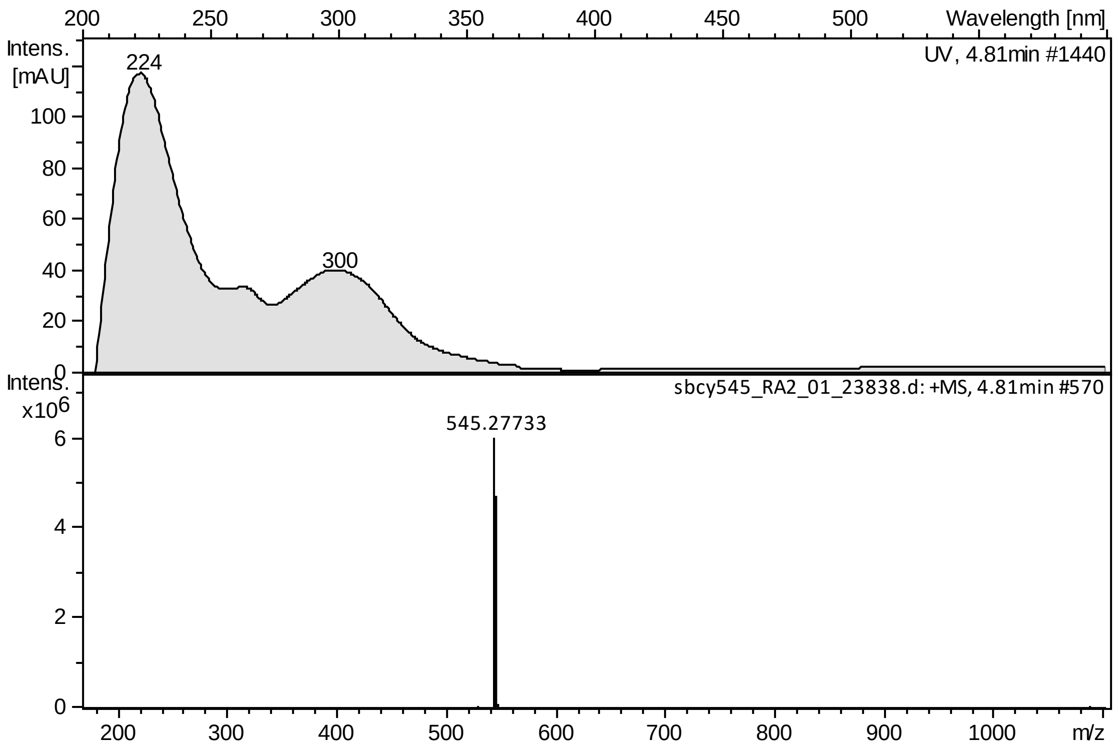

Figure S1. UV/VIS and partial ESI+MS spectra of purified stigmatellic acid (**1**).

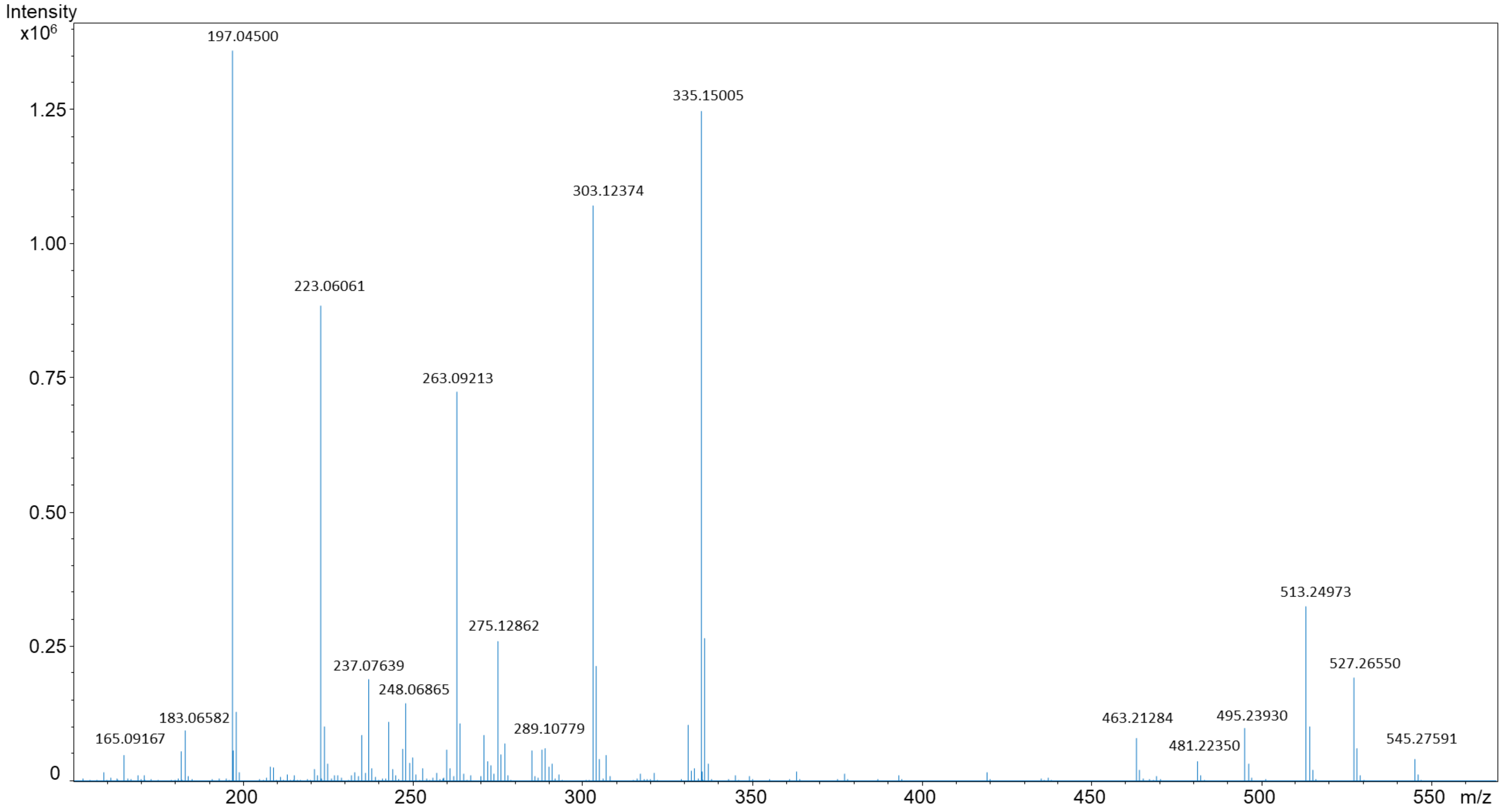

Figure S2. Fragmentation pattern in ESI+MS^2 experiment of stigmatellic acid (**1**).

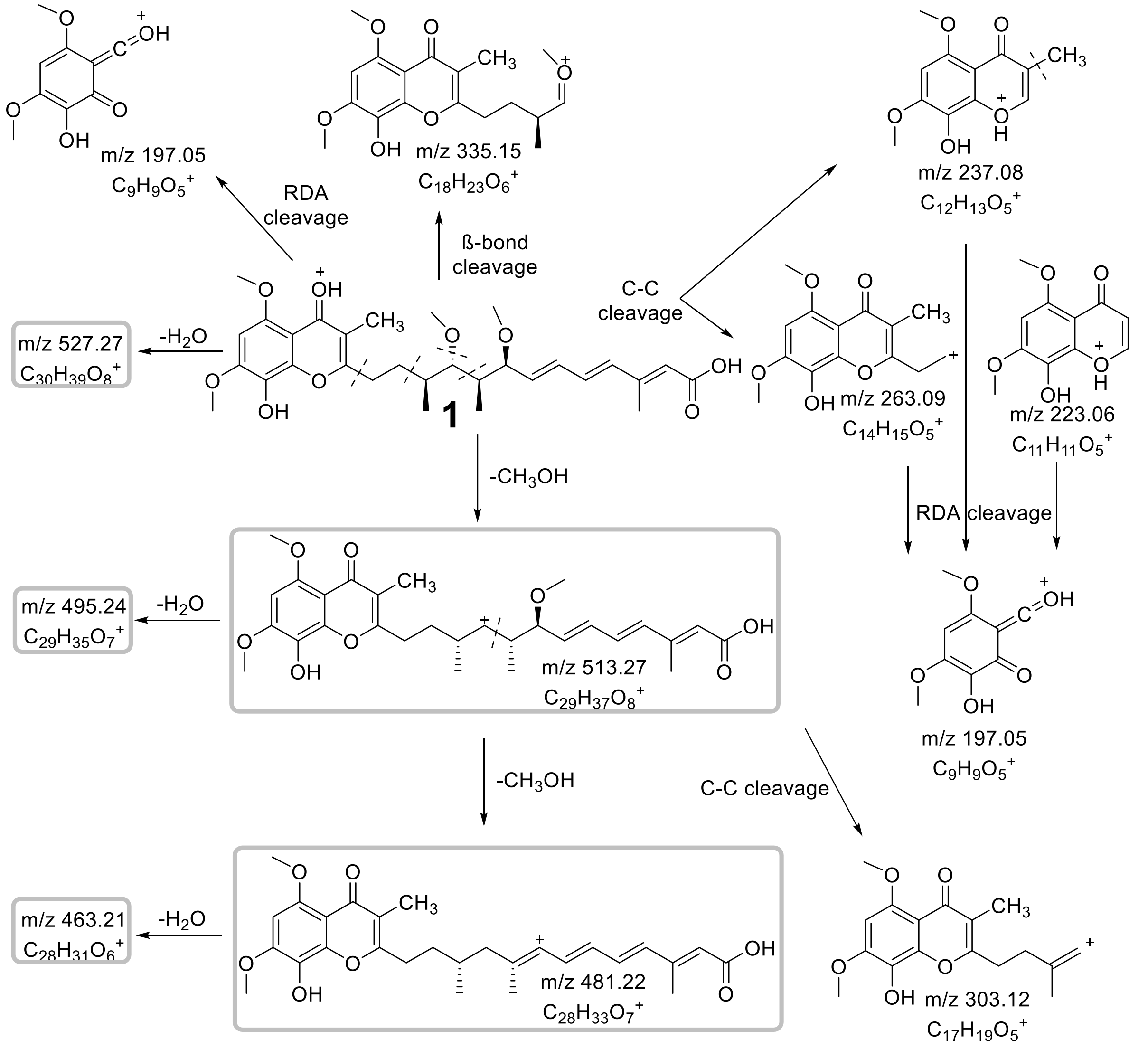

Figure S3. MS^2 fragmentation scheme of **1** and proposed structure of identified key ion fragments (ion fragments in grey box are specific for **1**). RDA: retro-Diels Alder.

molecules

MDPI

Supplementary Materials 3

Table S2. Spectroscopic values of stigmatellic acid (**1**) acquired in methanol-d_4 at 700 MHz.

Stigmatellic acid (**1**)					
**Position**	**δC**	**δH,** [m, *J* (Hz)]	**COSY**	**HMBC**	**ROESY**
1a	148.2	-	-	-	-
2	165.6	-	-	-	-
3	117.5	-	-	-	-
4	179.9	-	-	-	-
4a	108.4	-	-	-	-
5	154.0	-	-	-	-
6	93.9	6.62 s 1H	-	1a, 4a, 4, 5,8, 7,	H-5-O–, H-7-O–
7	152.7	-	-	-	-
8	129.1	-	-	-	-
9	10.2	1.98 s 3H	-	2, 3, 4	-
1’	30.6	2.84 m 1H<br>2.69 m 1H	H-2’	2, 3, 2’, 3’	-
2’	28.3	1.92 m 1H<br>1.55 m 1H	H-3’	2, 1’, 3’,4’ 14’	-
3’	35.6	1.71 m 1H	H-14’	1’,2’,4’, 15’	-
4’	88.7	3.11 dd 1H (2.3/9.4)	H-3’	2’, 3’, 5’, 6’,14’, 15’, 4’-OCH_3, 6’-OCH_3	H-14‘, H-15‘
5’	42.9	1.68 m 1H	H-15’	4’, 15’	-
6’	82.4	3.94 dd 1H (2.5/7.0)	H-5’, H-7’	4’, 5’, 7’, 8’, 15’, 6’O–	H-3`, H-5`
7’	138.3	5.86 dd 1H (7.0/15.6)	H-6’, H-8’	5’, 6’, 9’	-
8’	133.3	6.31 dd 1H (10.5/15.3)	H-9’	6’, 9’, 10’	-
9’	135.2	6.71 dd 1H (10.5/15.3)	H-8’,H-10’	7’, 8’, 11’	H-7’, H-16’
10’	136.8	6.32 d 1H (15.3)	H-9’	8’, 9’, 11’, 12’, 16’	H-6’, H-8’, H-12’
11’	153.4	-	-	-	-
12’	121.1	5.81 s 1H	-	10’, 11’, 13’, 16’	H-10’
13’	170.8	-	-	-	-
14’	18.4	1.15 d 3H (6.9)	H-3’	2’, 3’, 4’	-
15’	10.6	0.72 d 3H (7.1)	H-5’	4’, 5’, 6’	-
16’	14.0	2.28 d 3H (1.1)	-	10’, 11’, 12’, 13’	-
5O–	56.8	3.88 s 3H	-	5,6	-
7O–	57.1	3.98 s 3H	-	7	-
4’O–	62.0	3.47 s 3H	-	4’	-
6’O–	57.0	3.24 s 3H	-	6’	-

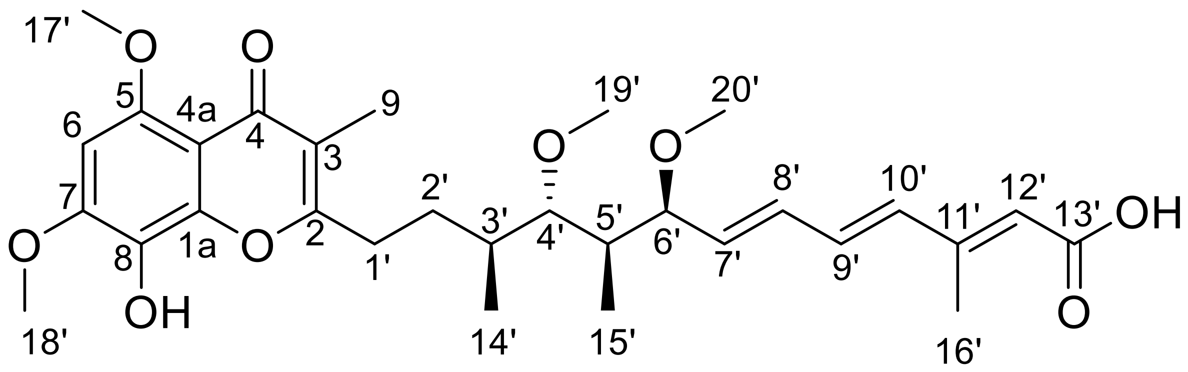

Stigmatellic acid
1

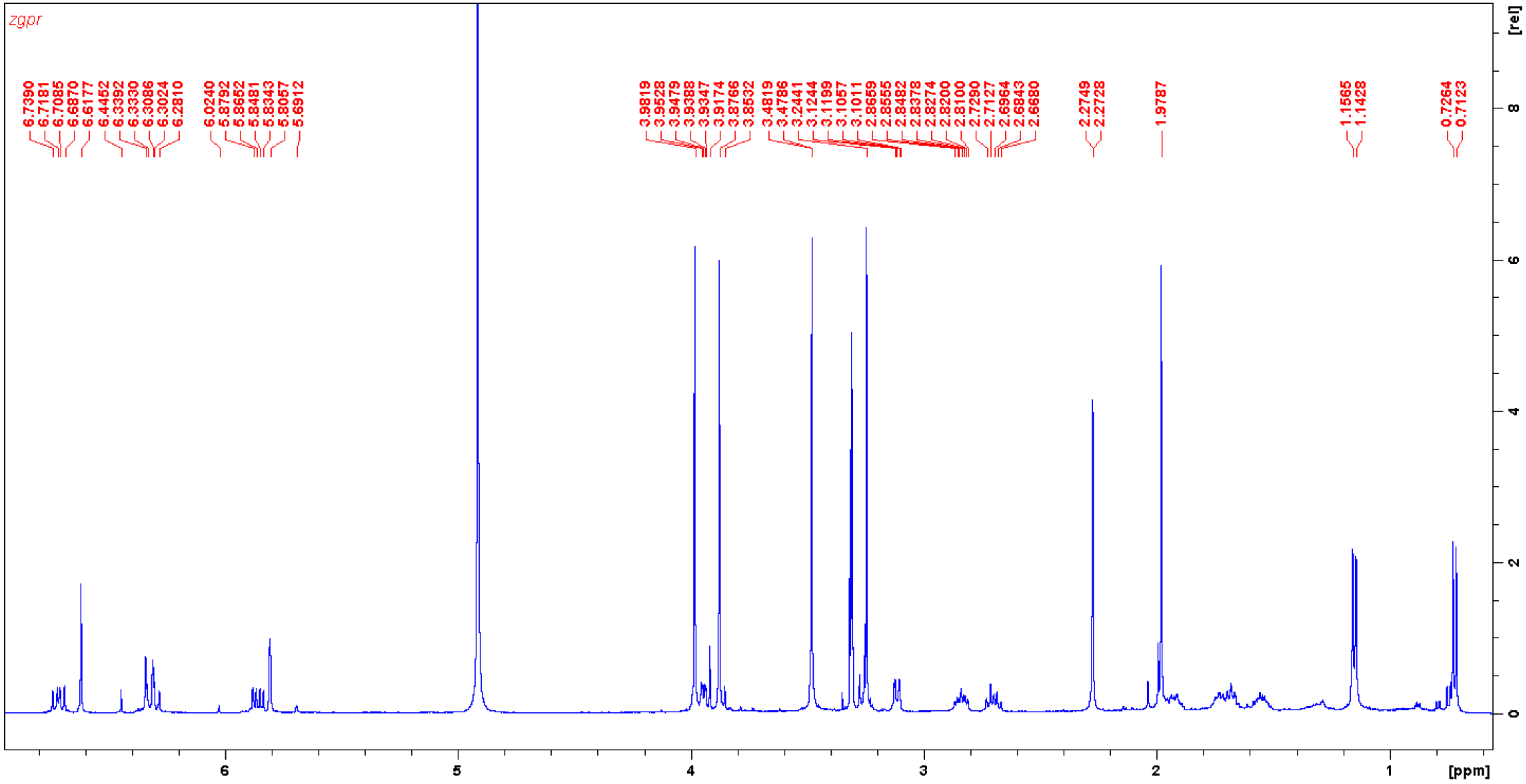

Figure S4. ^{1}H NMR spectrum of stigmatellic acid (**1**) in methanol-d$_4$.

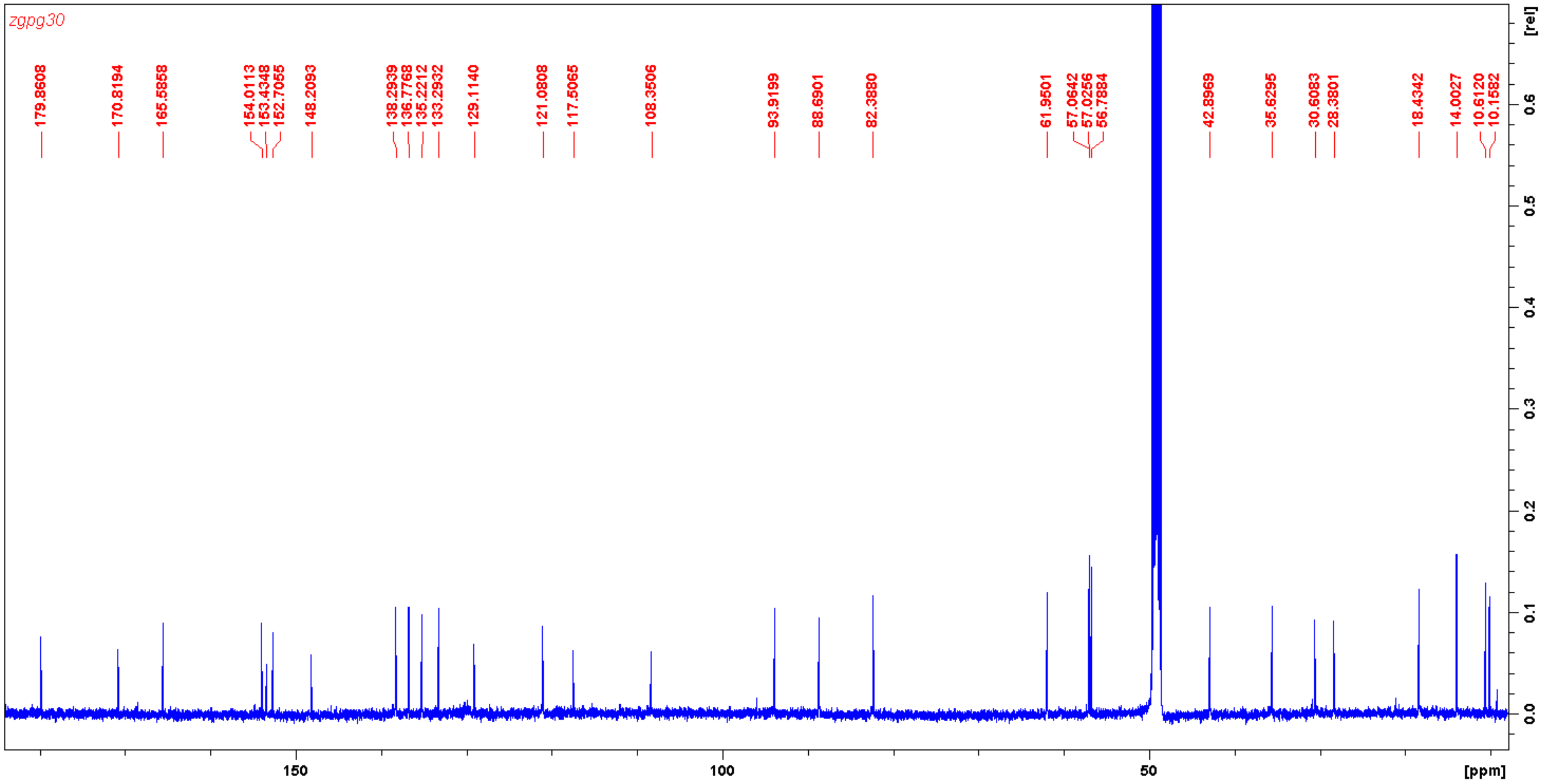

Figure S5. ^{13}C NMR spectrum of stigmatellic acid (**1**) in methanol-d_4.

molecules MDPI

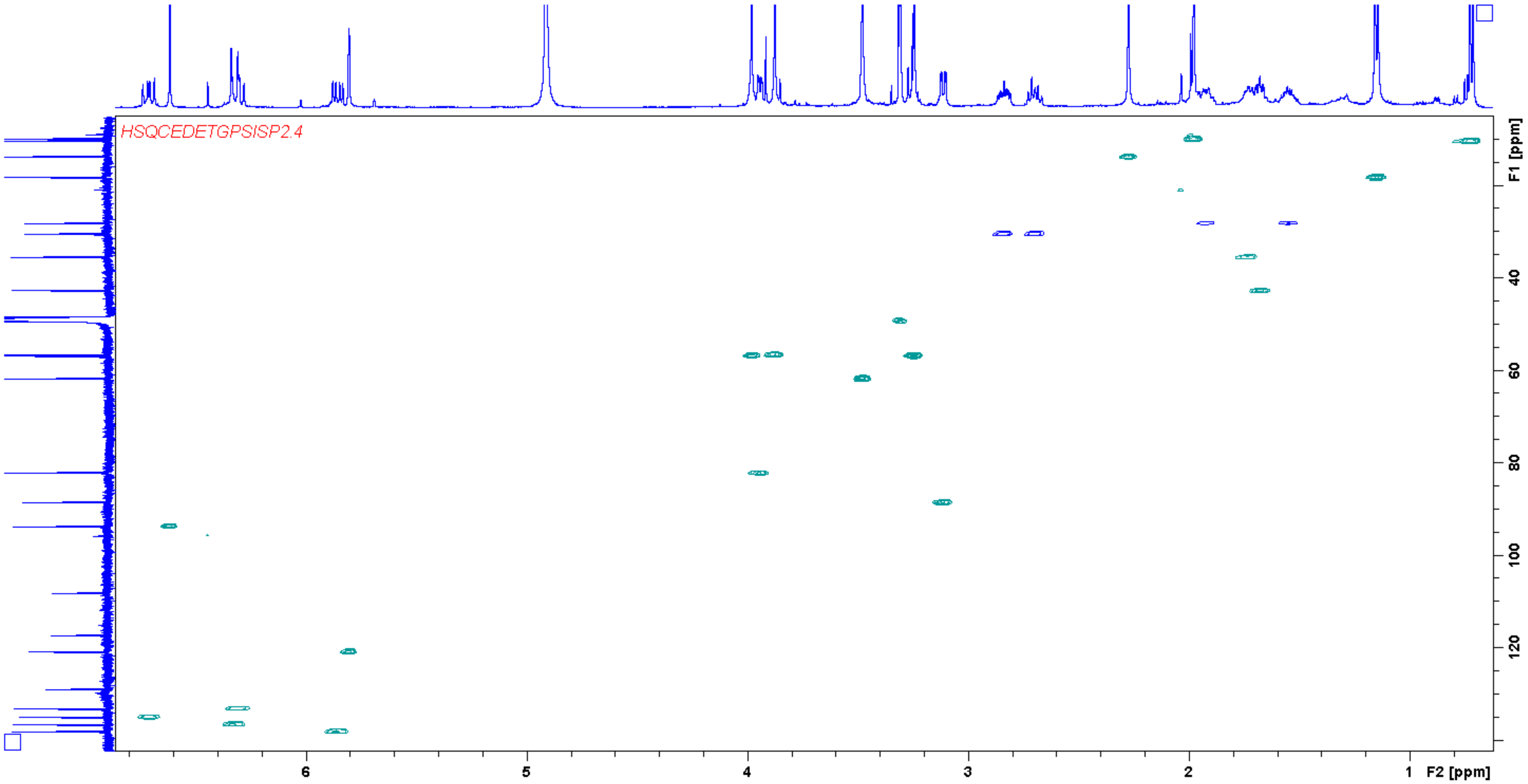

Figure S6. HSQC spectrum of stigmatellic acid (**1**) in methanol-d_4.

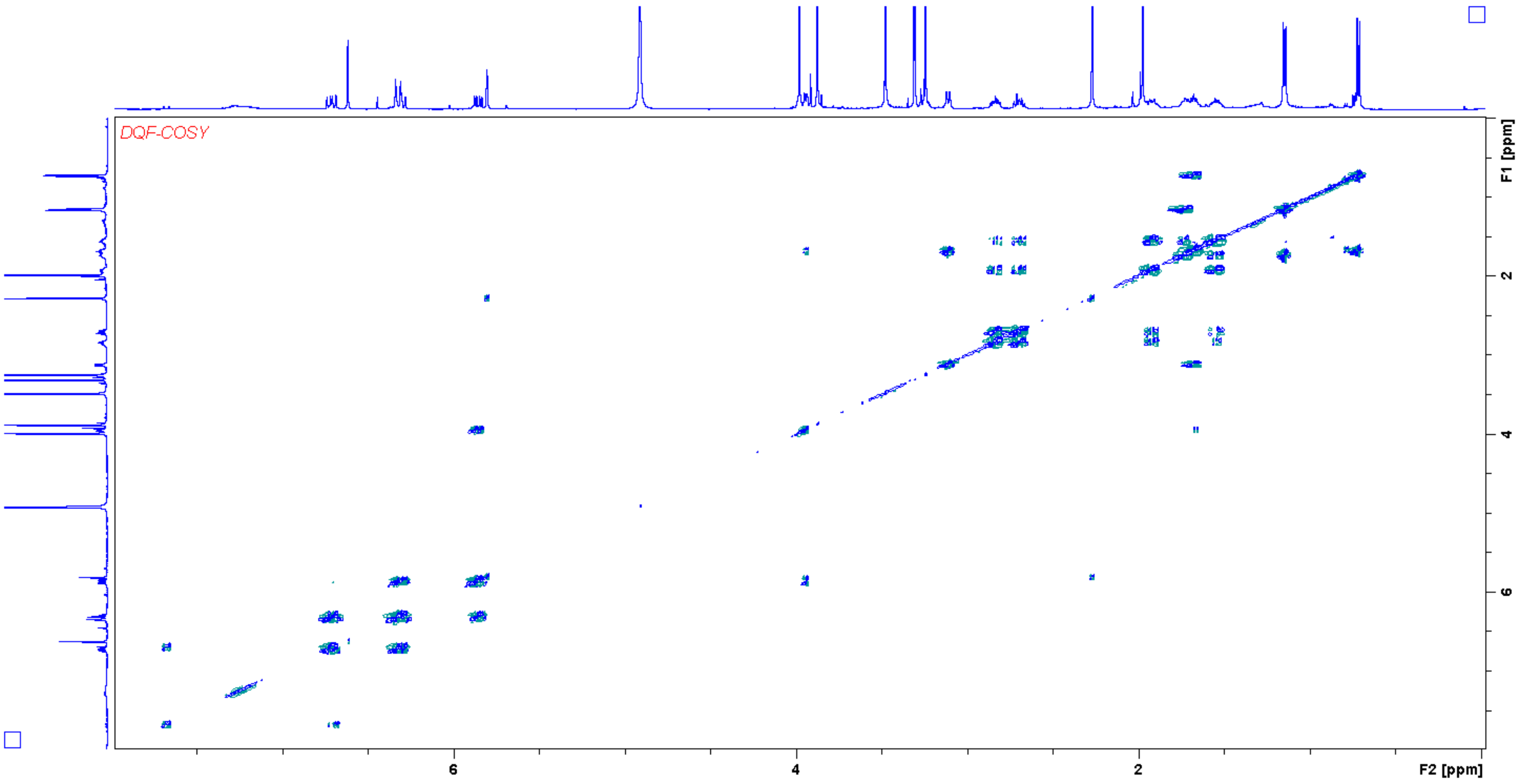

Figure S7. DQF-COSY spectrum of stigmatellic acid (**1**) in methanol-d_4.

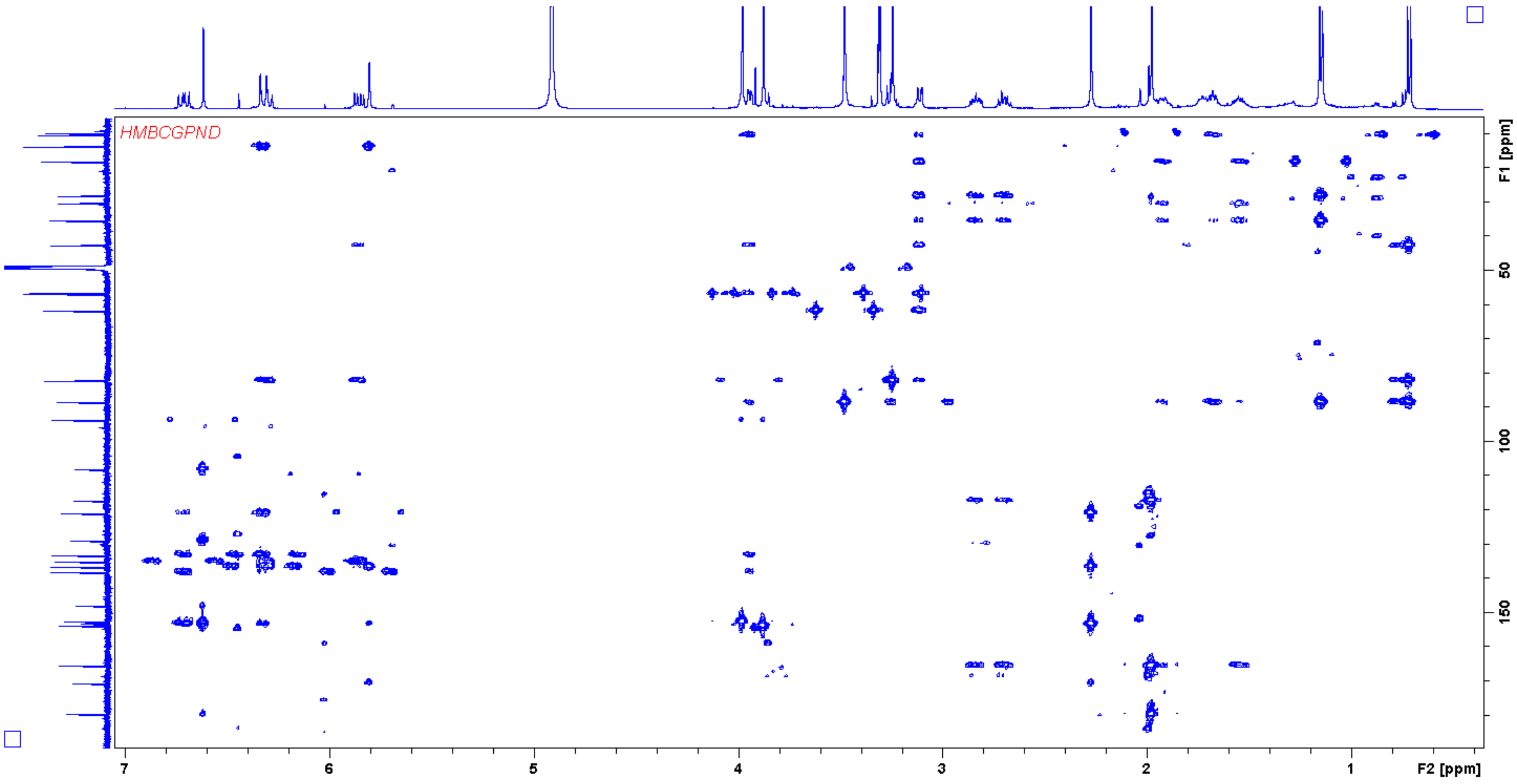

Figure S8. HMBC spectrum of stigmatellic acid (**1**) in methanol-d$_4$.

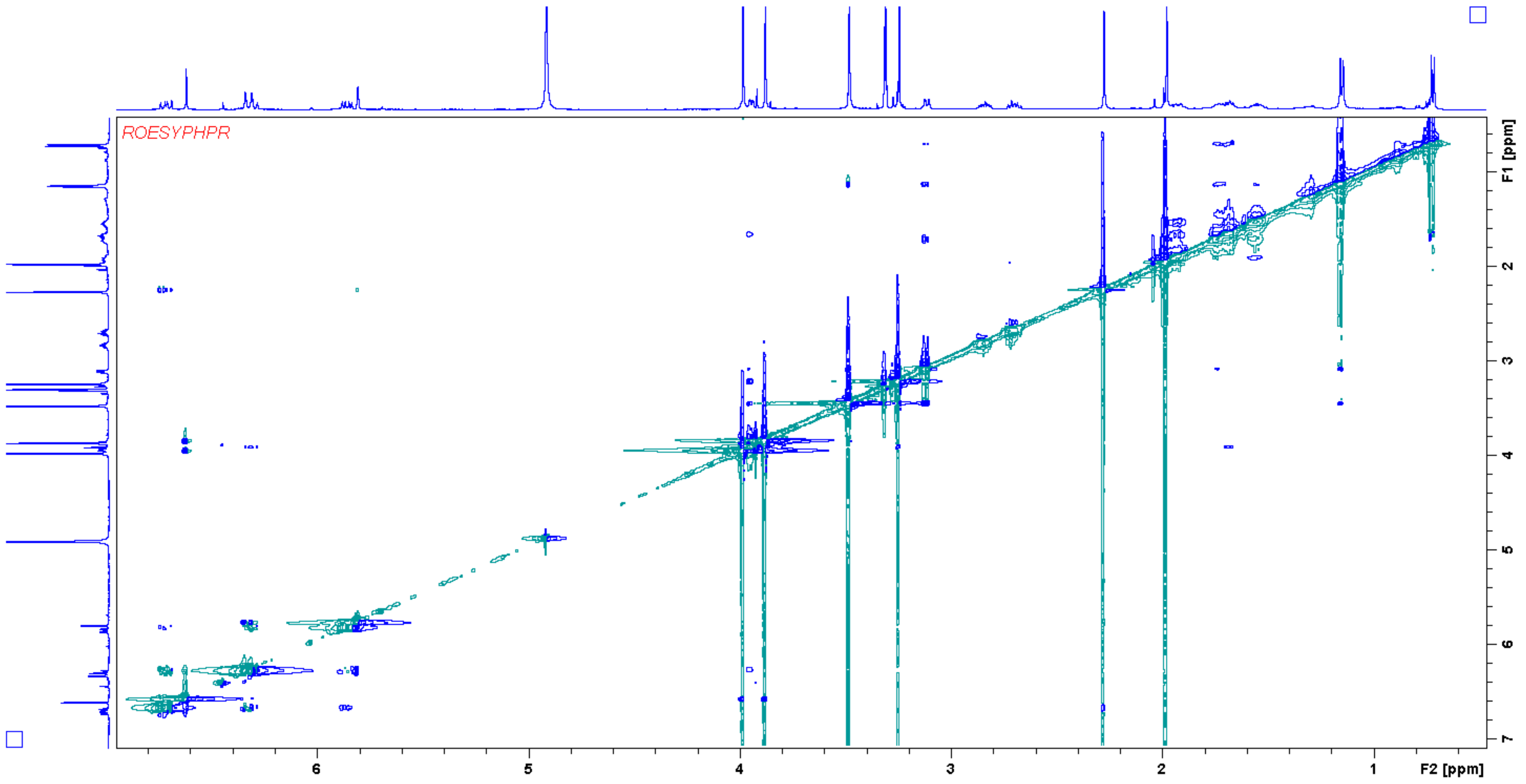

Figure S9. ROESY spectrum of stigmatellic acid (**1**) in methanol-d_4.

Supplementary Materials 4

Table S3 Alignment of KR domain sequences from the stigmatellin biosynthesis (MCy10943^{T}) and comparison of predicted and observed product stereochemistry. Influential residues in the sequence are shown in bold letters. Since the predicted stereospecificity of the KR domains involved in the stigmatellin biosynthesis in the myxobacterial strain *V. cumulatum* MCy10943^{T} is – according to previously identified sequence motifs within KR domains [3] – identical to the homologs in the myxobacterial strain *S. aurantiaca* Sg a15 (**Table S7**), the absolute configuration of **1** and **3** might resemble the absolute configuration of **4**.

Gene	Module	Loop region	Catalytic region	KR type	Pred.	Obs.
stiA	1	HAAGL**LDD**GTVLQL	S**S**SASLVGMPGQGN**Y**AAANA	**B**	active (C^S)	C=C (*E*)
stiB	2	HAAGV**LDD**GVLLRQ	S**S**MASALGSRGQGN**Y**AAANA	**B**	C^S–OH	C=C (*E*)
stiC	3	HAAGVVQDGALLRQ	S**S**AASLLGPRGQGN**Y**AAGNA	**?**	active	C=C (*E*)
stiD	4	HAAGVAHPRSLVEL	S**S**GSALMSSPMLGG**Y**AAANS	**?**	active	C^S–OH
stiE	5	HSAVV**LDD**GVLLQQ	S**S**LASMLGAPGQGN**Y**TAAGA	**B1**	active	C^S–OH
stiF	6	HSAGV**LDD**GVLRQ	S**S**VASLFGSASQGN**Y**AAANAF	**B**	active (C^S)	CH_2
stiH	7	HTGGPWTVRASREL	S**S**VASVWGSGGLSHHAAAHH	---	inactive	C=O

Table S4 Alignment of KR domain sequences from the stigmatellin biosynthesis (Sg a15) and comparison of predicted and observed product stereochemistry. Influential residues in the sequence are shown in bold letters.

Gene	Module	Loop region	Catalytic region	KR type	Pred.	Obs.
stiA	1	HAAGL**LDD**GTVLQL	S**S**AASLVGIPGQGN**Y**AAGNA	**B**	active (C^S)	C=C (*E*)
stiB	2	HAAGV**LDD**GVLIRQ	S**S**MASALGSRGQGN**Y**AAANA	**B**	C^S–OH	C=C (*E*)
stiC	3	HAAGVVQDGALLRQ	S**S**AASLLGPRGQGN**Y**AAGNA	**?**	active	C=C (*E*)
stiD	4	HAAGVANPKPLADL	S**S**GSALMSSPLLGG**Y**AAANAF	**?**	active	C^S–OH
stiE	5	HSAVV**LDD**GVLLKQ	S**S**LASMLGAPGQGN**Y**TAAGA	**B1**	active	C^S–OH
stiF	6	HSAGV**LDD**GVLRQ	S**S**IASMFGSASQGN**Y**AAANAF	**B**	active (C^S)	CH_2
stiH	7	HAGGAWTPRASRD	S**S**VASVWGSGGLSHHAAAHH	---	inactive	C=O

molecules

MDPI

Supplementary Materials 5

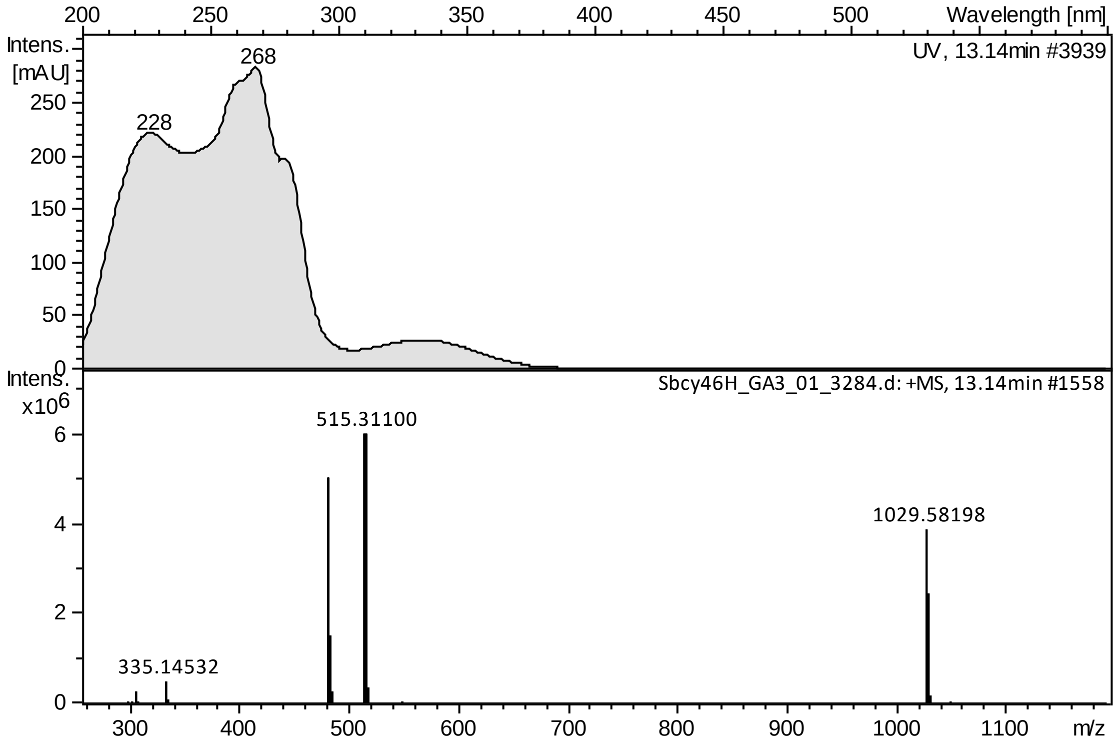

Figure S10. UV/VIS and partial ESI+MS spectra of purified *iso*-methoxy stigmatellin (**2**).

molecules MDPI

Supplementary Materials 6

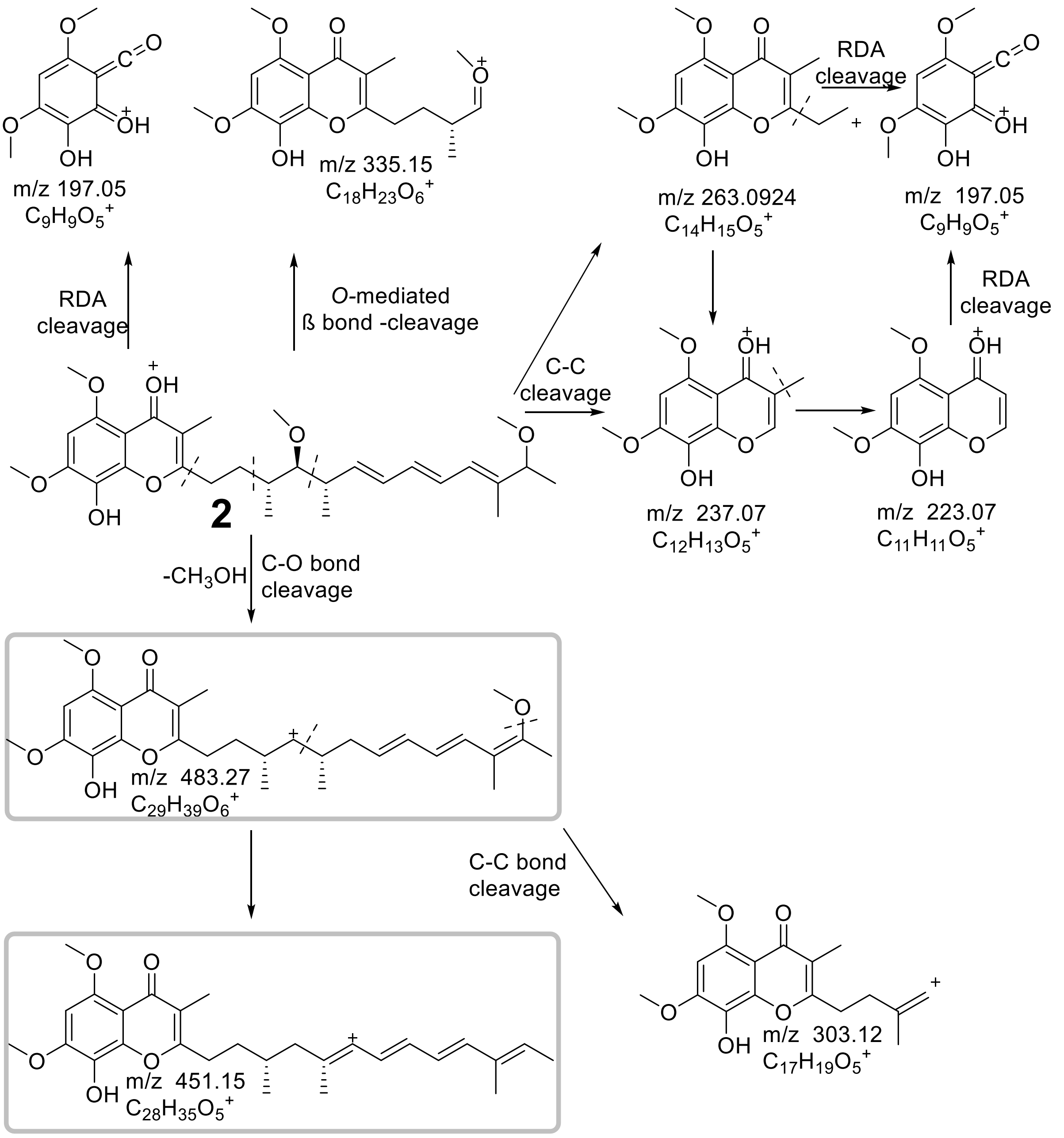

Figure S11. MS[2] fragmentation scheme of **2** and proposed structure of identified key ion fragments (ion fragments in grey box have been observed specifically for **2** and **4**). RDA: retro-Diels Alder.

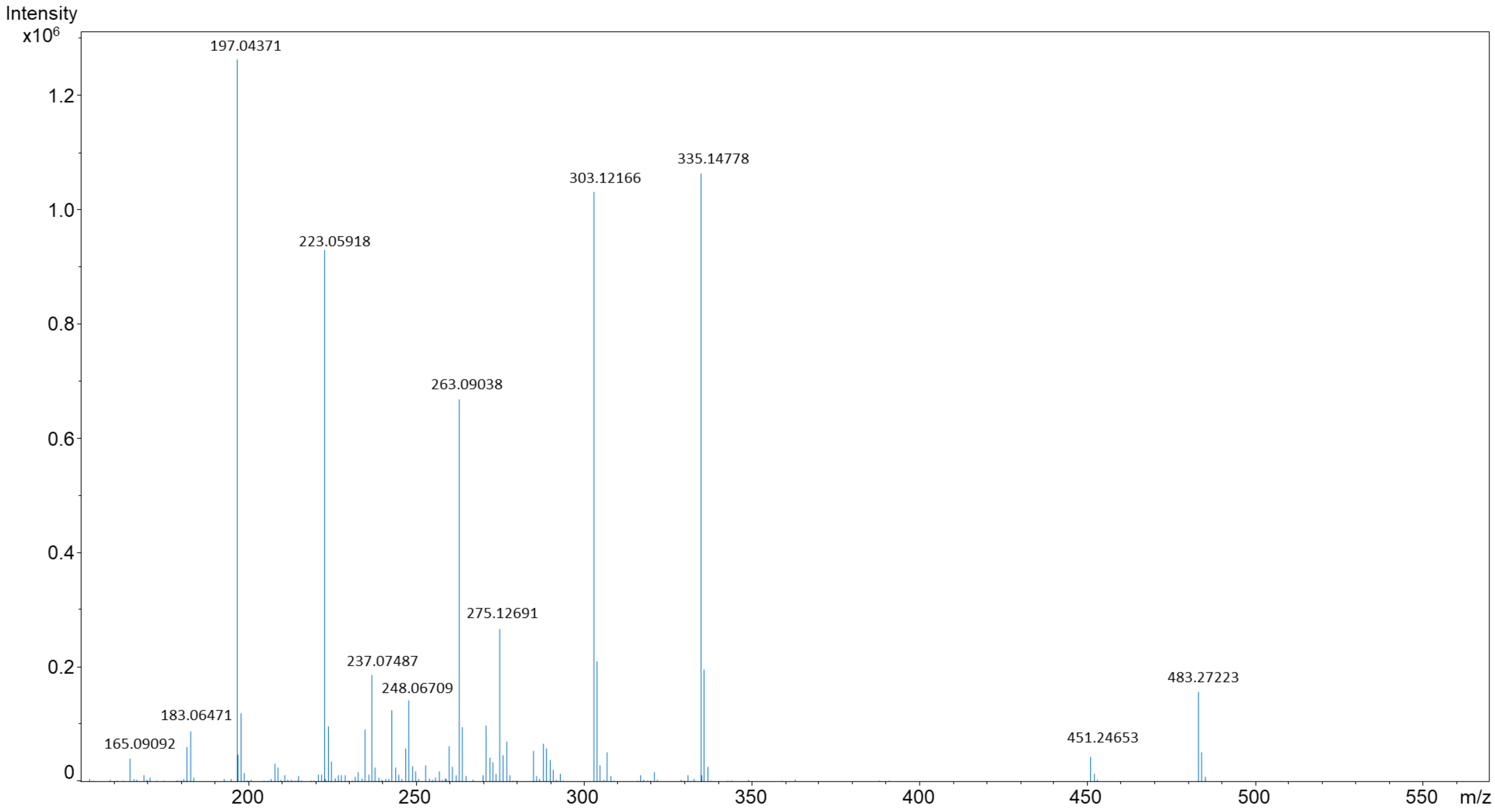

Figure S12. Fragmentation pattern in ESI+MS^2 experiment of *iso*-methoxy stigmatellin A (**2**).

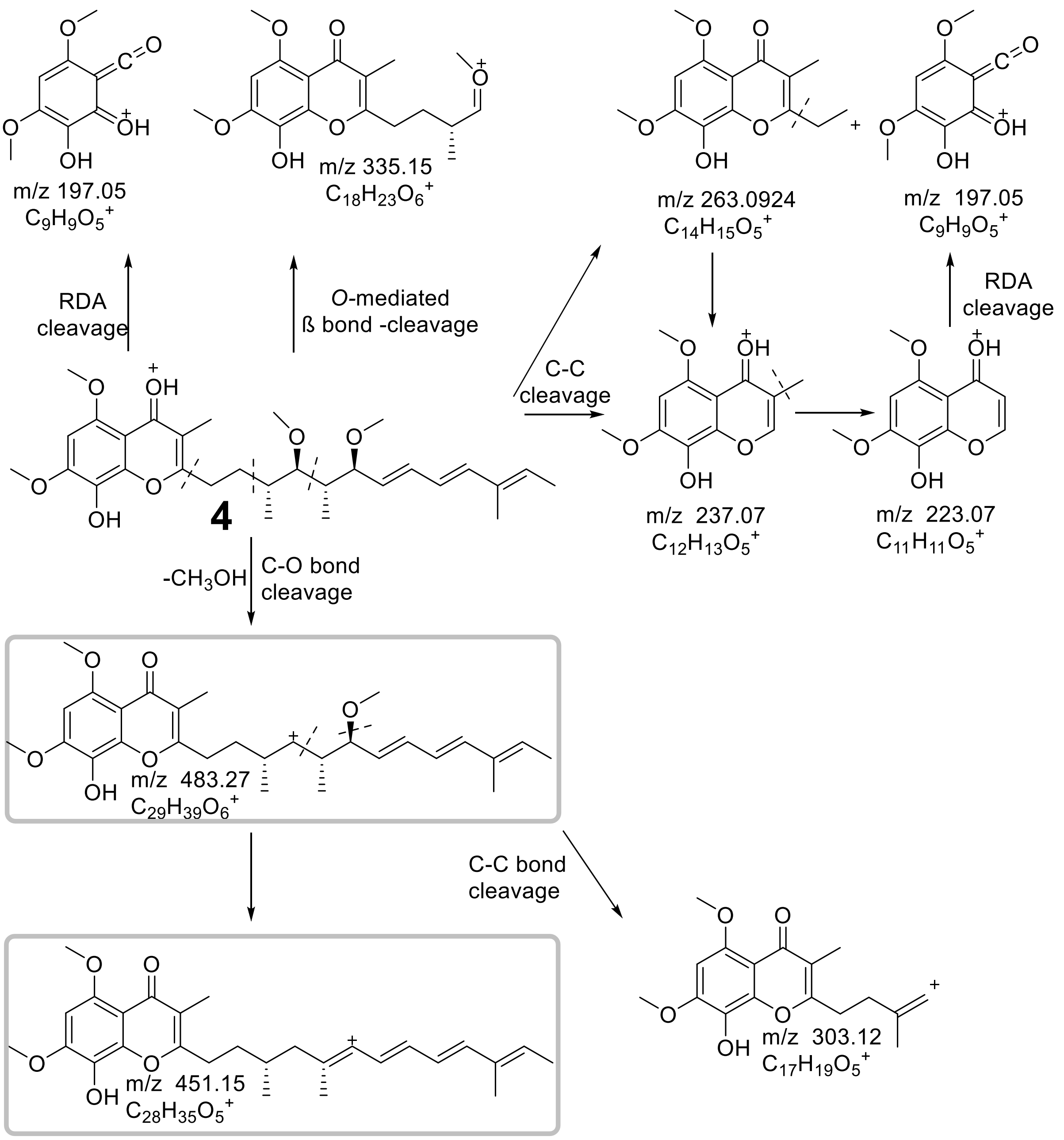

Figure S13. MS^2 fragmentation scheme of **4** and proposed structure of identified key ion fragments (ion fragments in grey box have been observed specifically for **2** and **4**). RDA: retro-Diels Alder.

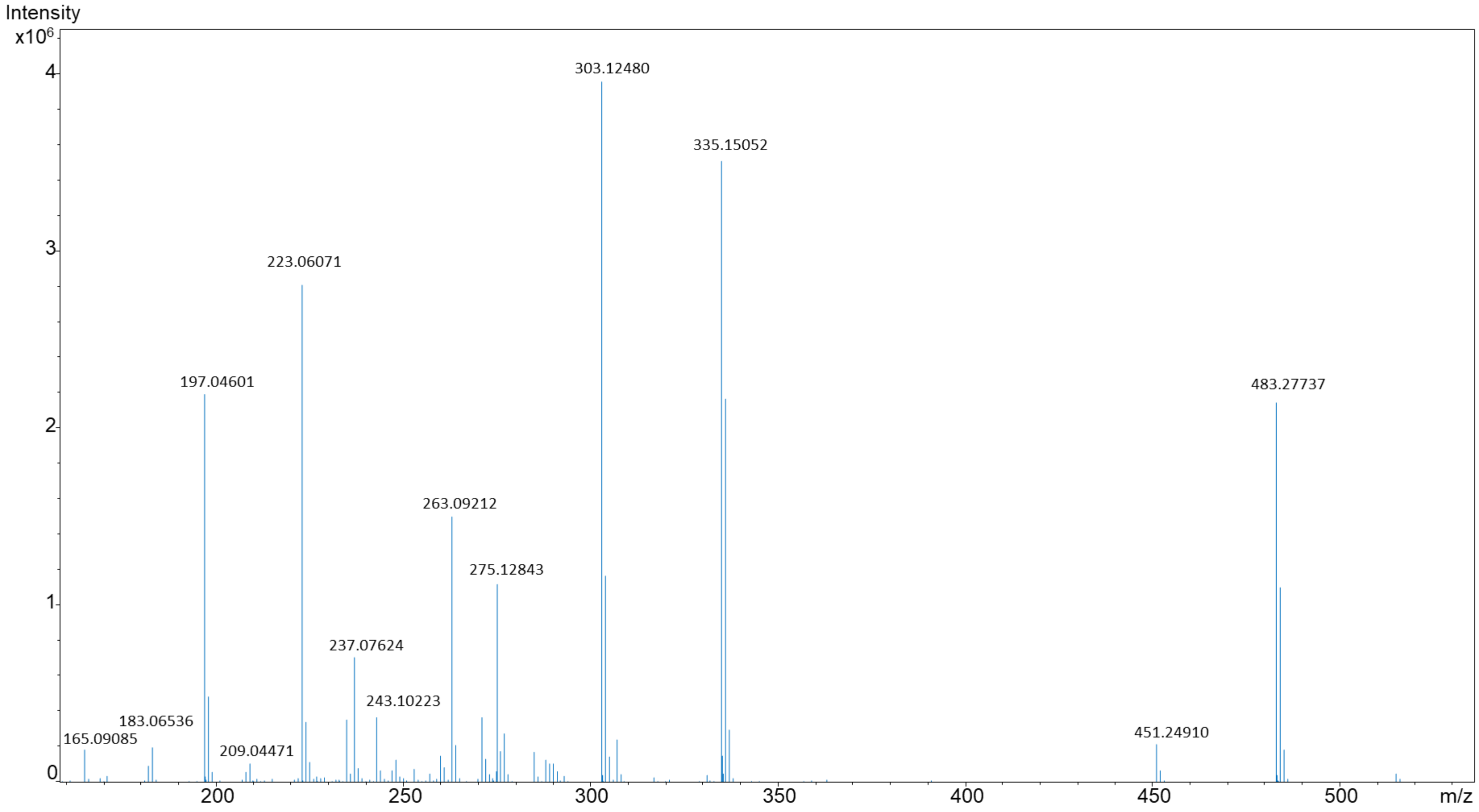

Figure S14. Fragmentation pattern in ESI+MS^2 experiment of stigmatellin A (**4**).

molecules

MDPI

Supplementary Materials 7

Table S5. Spectroscopic values for *iso*-methoxy stigmatellin (**2**) acquired in methanol-d_4 at 700 MHz.

Iso-methoxy-stigmatellin (**2**)				
**Position**	**δC**	**δH,** [m, *J* (Hz)]	**COSY**	**HMBC**
1a	148.1	-	-	-
2	165.5	-	-	-
3	117.2	-	-	-
4	179.7	-	-	-
4a	108.2	-	-	-
5	153.9	-	-	-
6	93.8	6.62 s 1H	-	4a,5,7 8, 1a,
7	152.5	-	-	-
8	128.9	-	-	-
9	9.9	1.98 s 3H	-	1', 2, 3,4
1'	30.6	2.84 m 1H<br>2.69 m 1H	H-2'	2',3', 2,3
2'	30.4	2.06 m 1H<br>1.56 m 1H	H-3'	1', 3', 4',14',
3'	37.2	1.67 m 1H (1.1)	H-14'	1',2', 4',14'
4'	91.6	2.86 m 1H	H-3',H-5'	2', 3', 5', 6', 4'-O–, 14',15'
5'	41.3	2.51 m 1H	H-6'	4', 6',7',15'
6'	137.7	5.69 m 1H	H-7'	4',5',7',8',15'
7'	132.1	6.14 dd 1H	H-6',H-8'	5', 6',8',9'
8'	127.7	6.36 m 1H	H-7,H-9'	6', 7',9',10'
9'	134.5	6.14,m, 1H	H-8',H-10'	7',8',10', 11'
10'	128.4	6.01, d, 1H(11.0)	H-9'	8',9',11'12',16'
11'	138.7	-	-	-
12'	83.9	3.37 s 1H (6.4)	H-13'	10',11'12'-O–,13',16',
13'	20.3	1.21 dd 3H (0.5/6.4)	H-12'	11';12'
14'	16.8	1.01 d 3H (6.5)	H-3'	2',3',4',
15'	18.4	1.04 d 3H (6.9)	H-5'	4',5', 6'
16'	11.1	1.67 d 3H (1.1)	-	10',11',12',13'
5O–	56.6	3.88 s 3H	-	5
7O–	56.9	4.00 s 3H	-	7
4'O–	61.7	3.45 s 3H	-	4'
6'O–	56.1	3.17 s 3H	-	12'

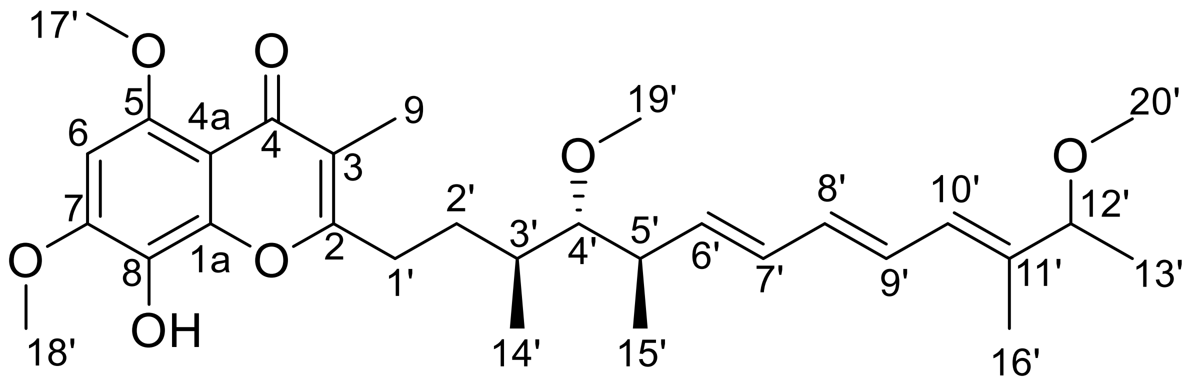

Iso-methoxy-stigmatellin A
2

molecules

MDPI

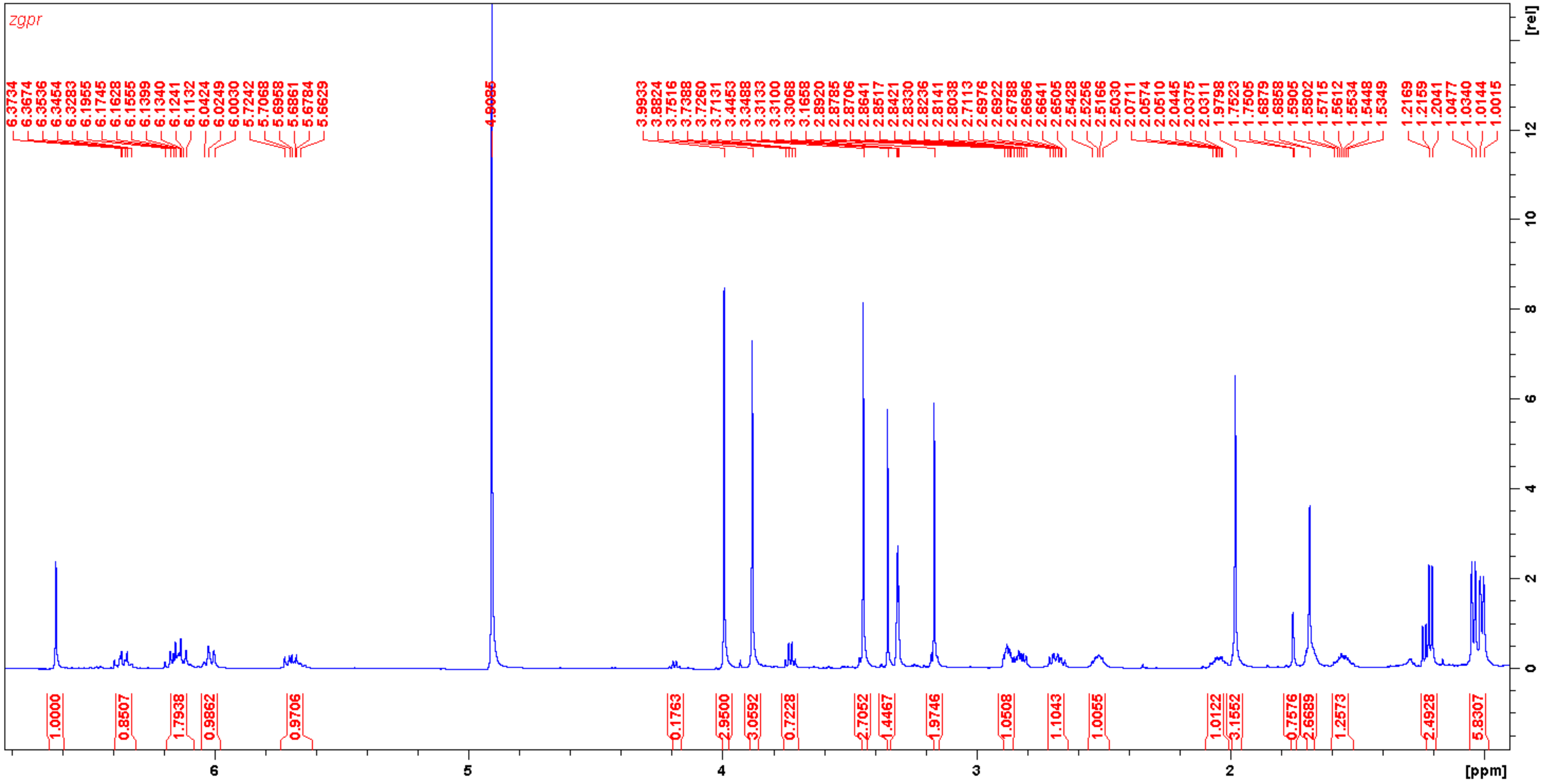

Figure S15. ^{1}H NMR of *iso*-methoxy stigmatellin A (**2**) in methanol-d_4.

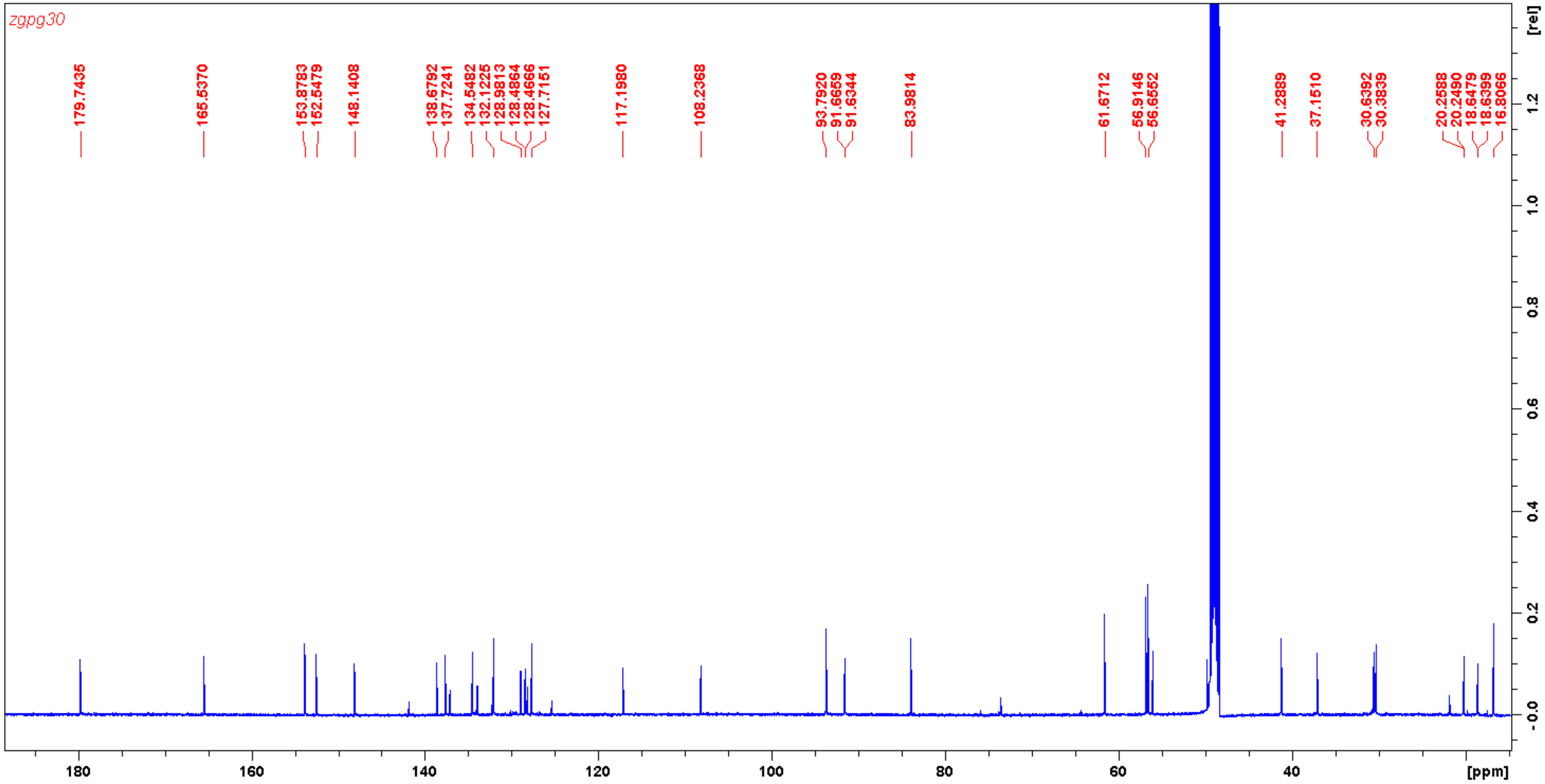

Figure S16. ^{13}C NMR of *iso*-methoxy stigmatellin A (**2**) in methanol-d_4.

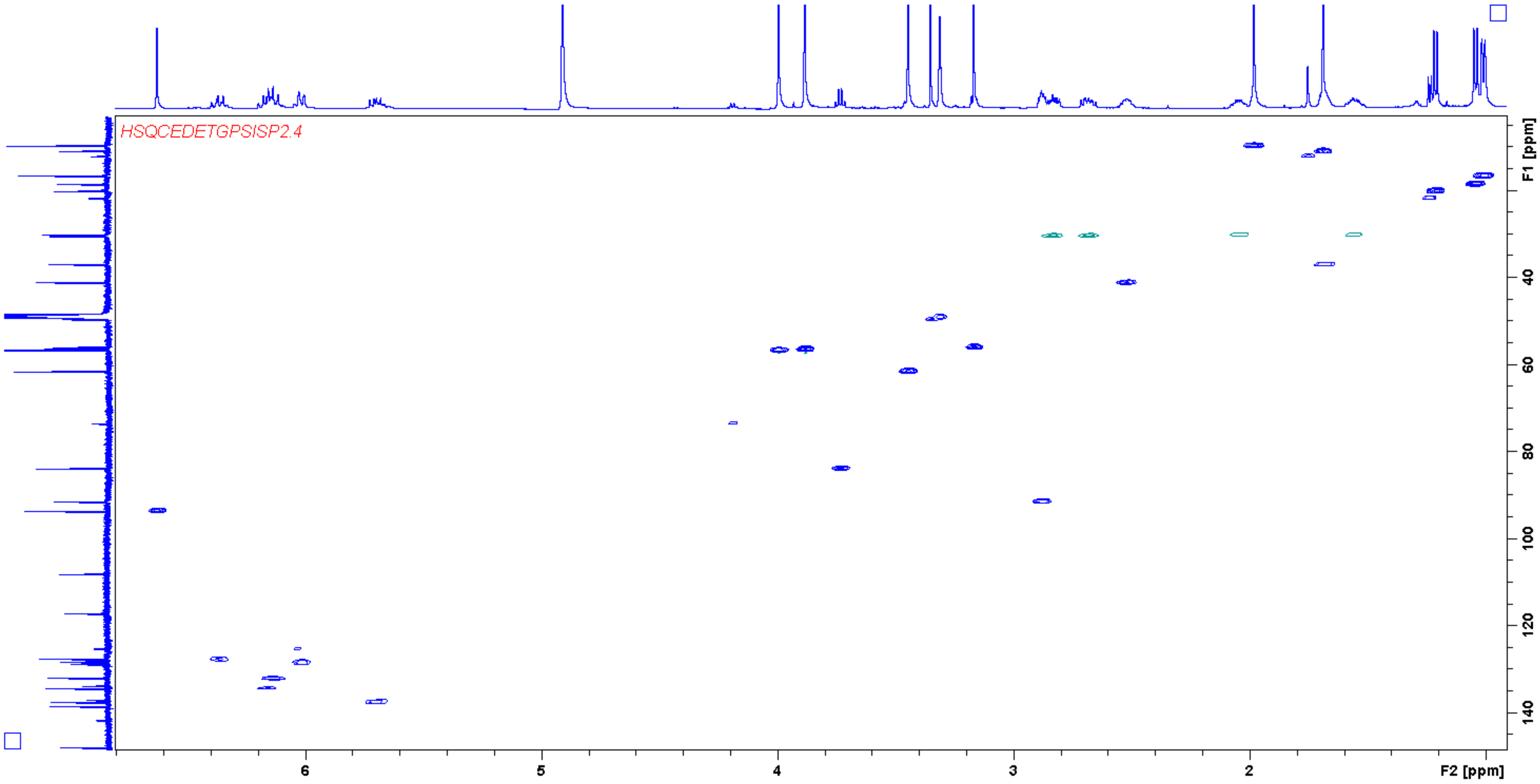

Figure S17. HSQC spectrum of *iso*-methoxy stigmatellin A (**2**) in methanol-d$_4$.

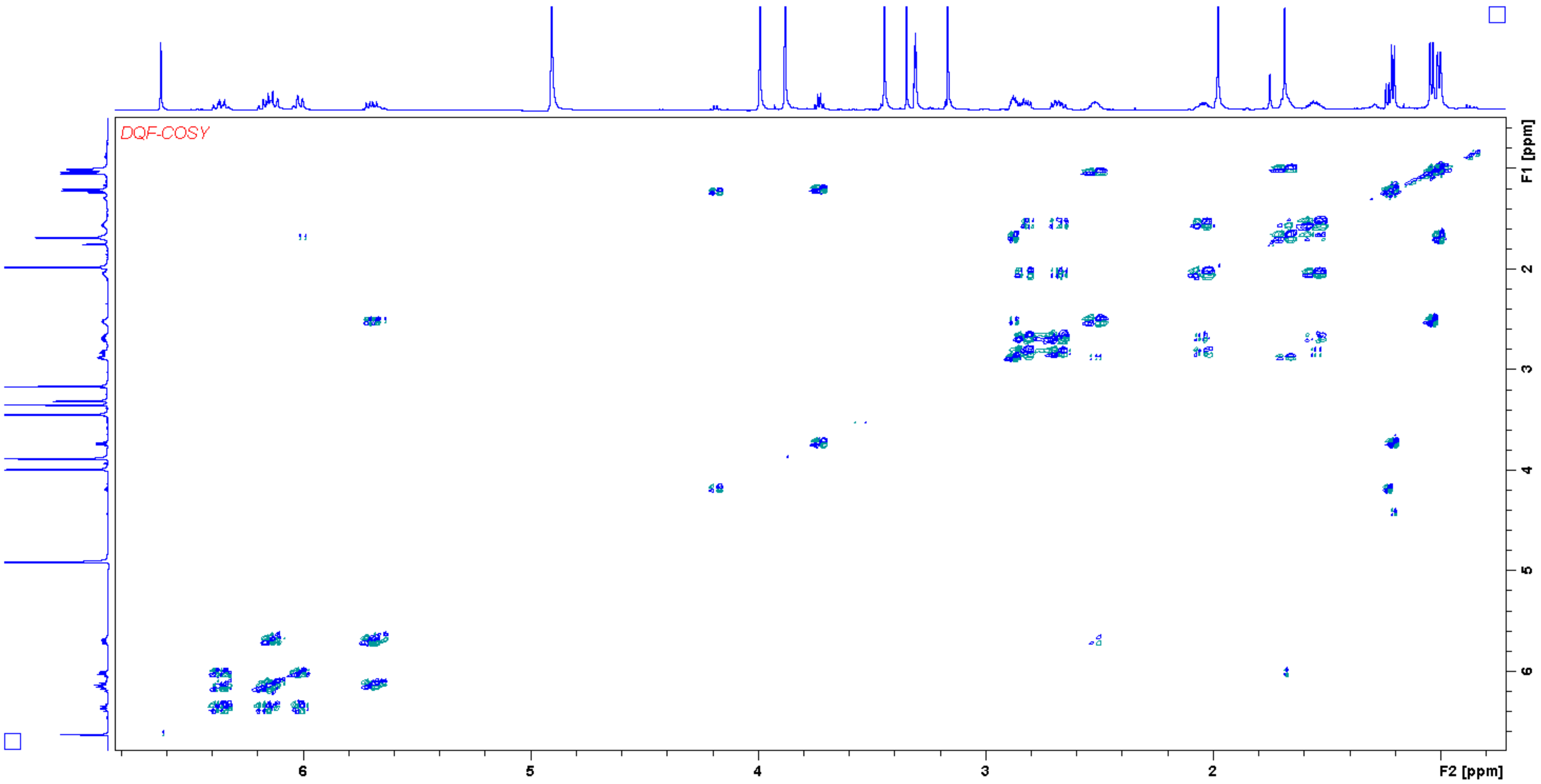

Figure S18: DQF-COSY spectrum of *iso*-methoxy stigmatellin A (**2**) in methanol-d$_4$.

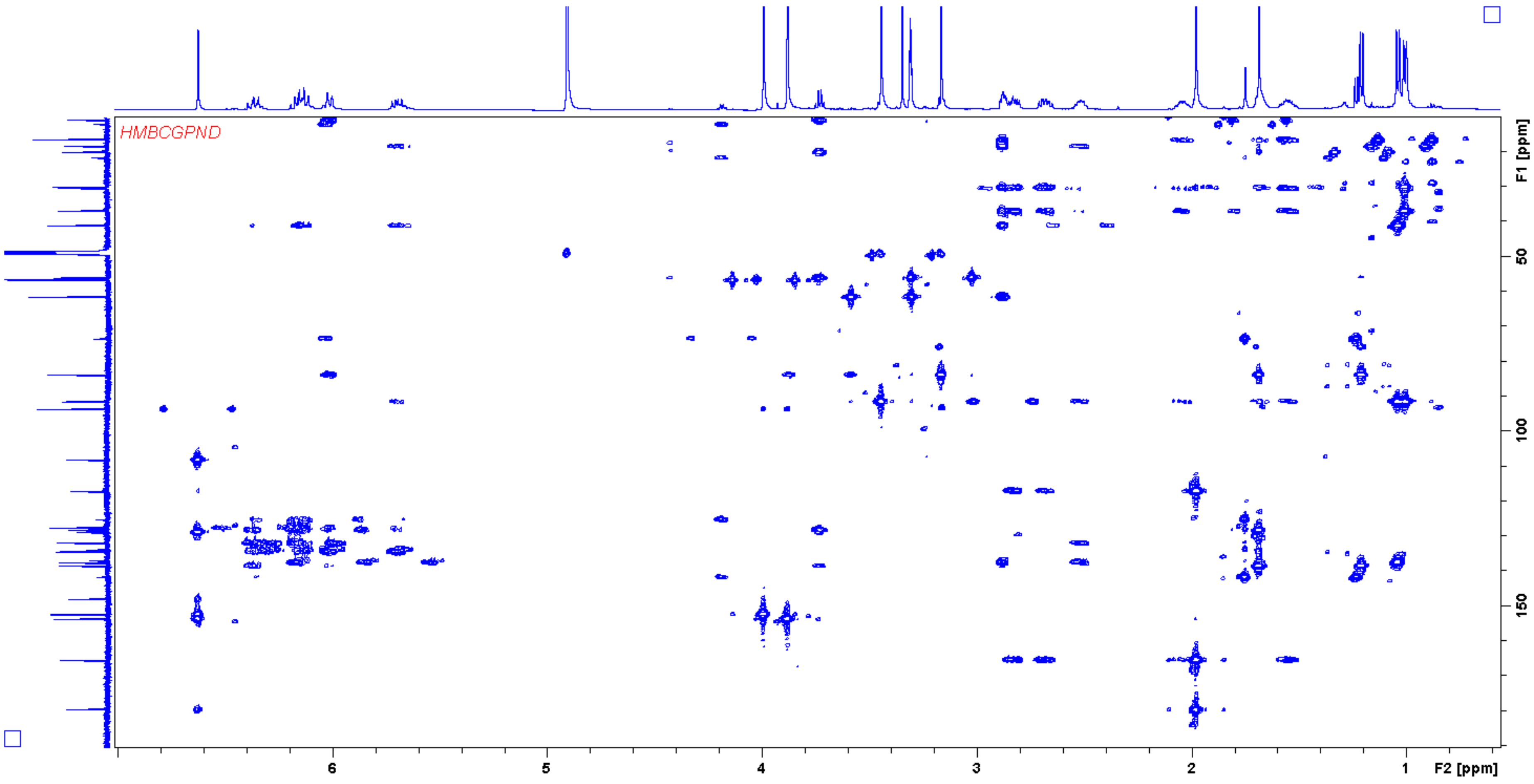

Figure S19. HMBC spectrum of *iso*-methoxy stigmatellin A (**2**) in methanol-d4.

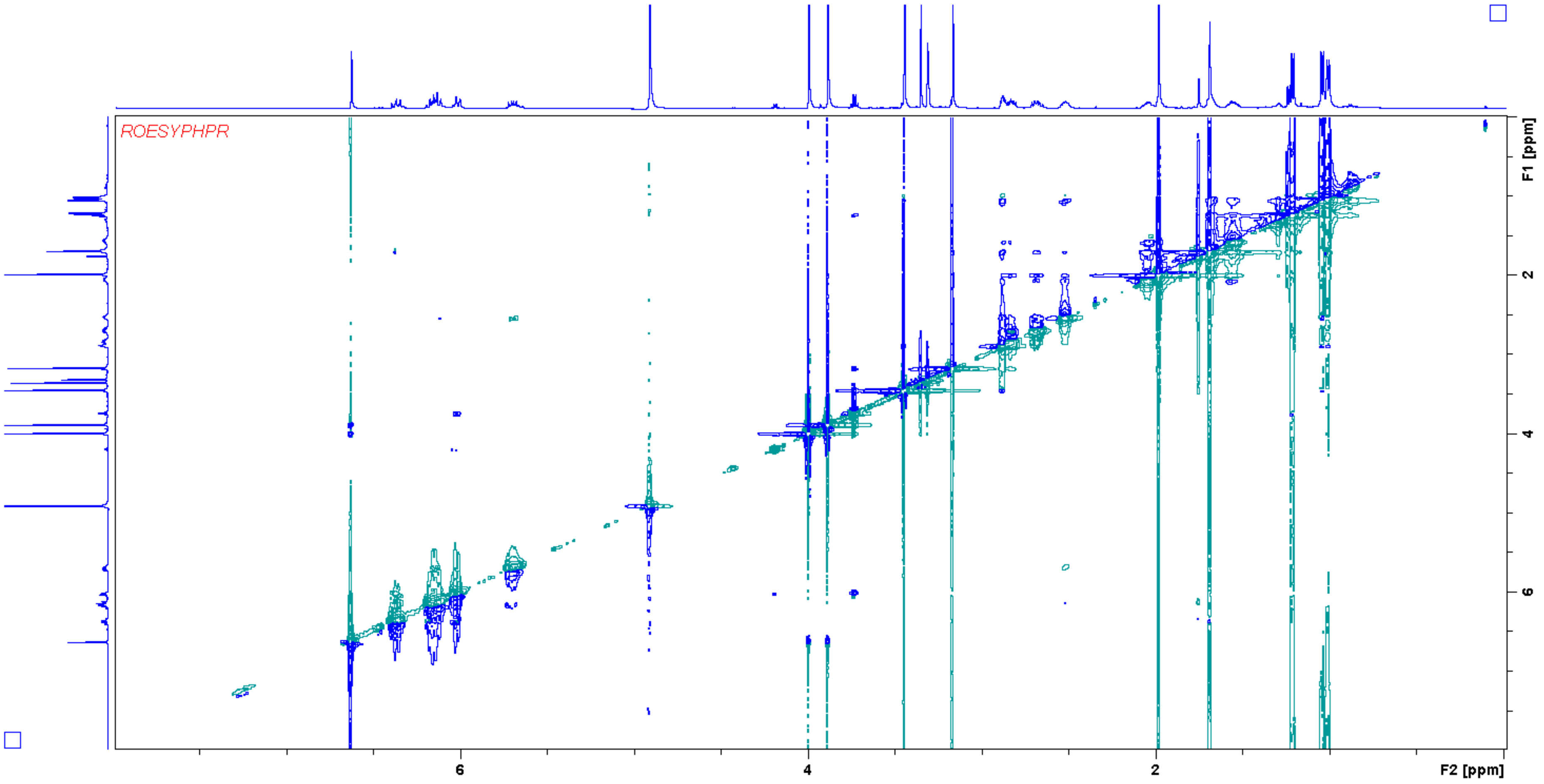

Figure S20. ROESY spectrum of *iso*-methoxy stigmatellin A (**2**) in methanol-d$_4$.

Supplementary Materials 8

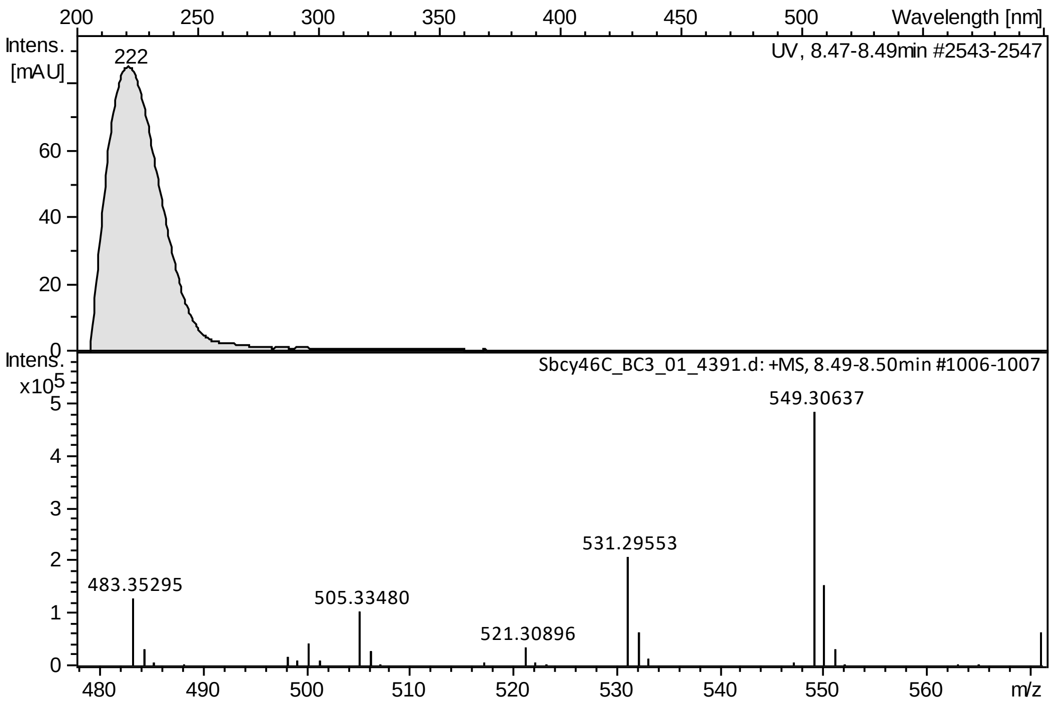

Figure S21. UV/VIS and partial ESI+MS spectra of purified stigmatellin C (isomer 1, **3**).

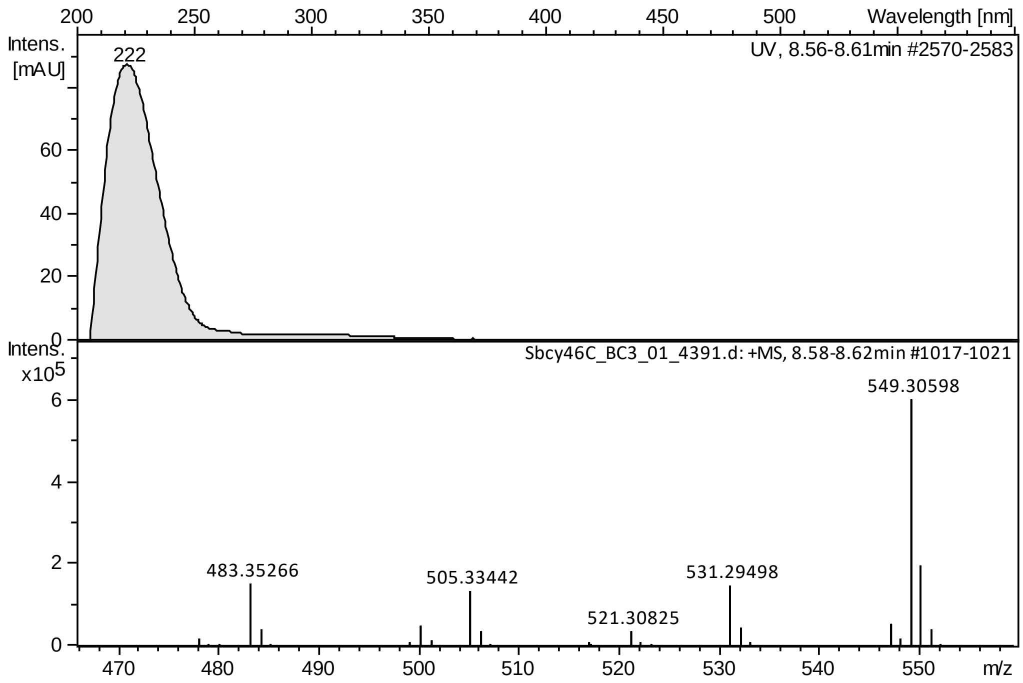

Figure S22. UV/VIS and partial ESI+MS spectra of purified stigmatellin C (isomer 2, **3**).

molecules

MDPI

Supplementary Materials 9

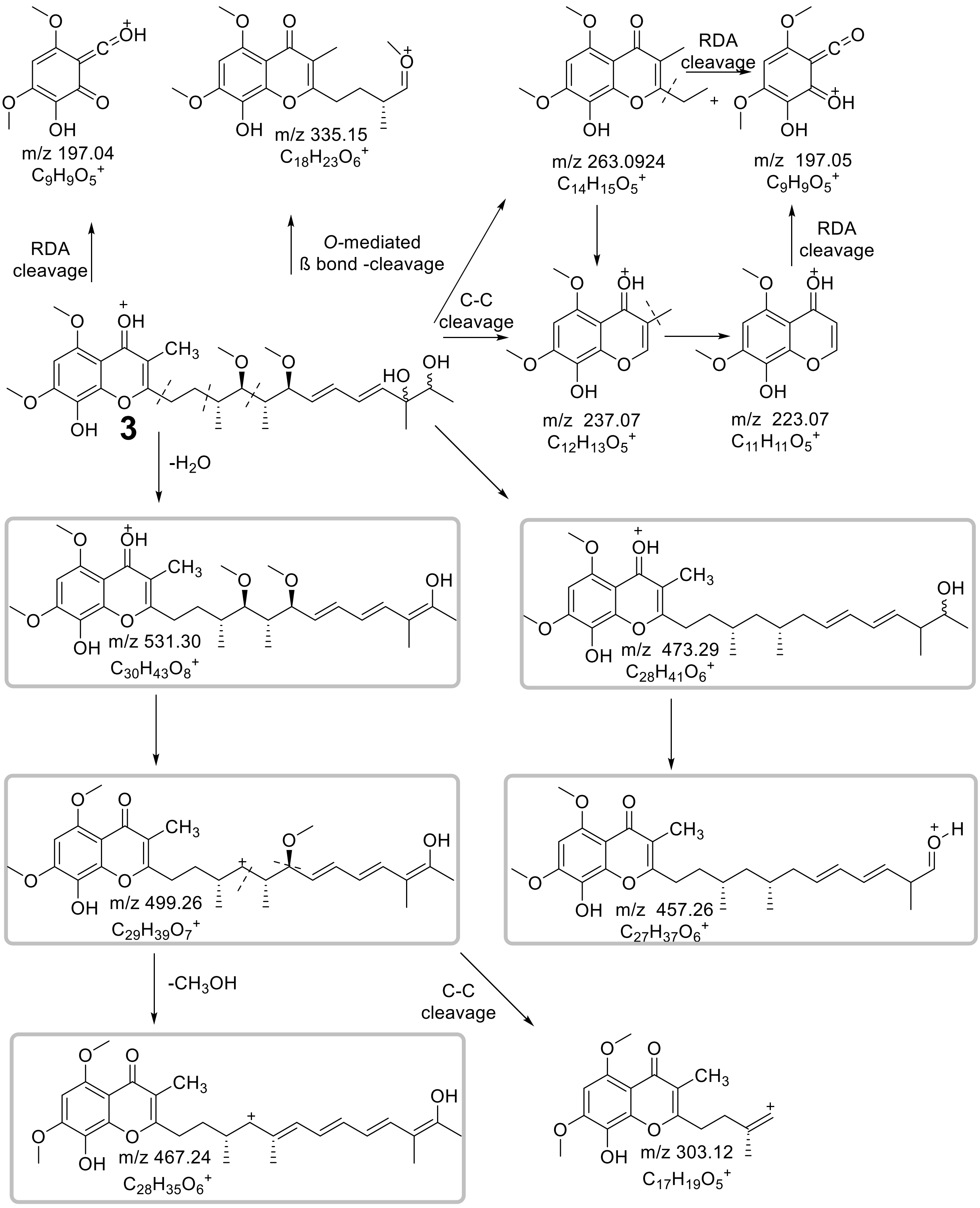

Figure S23. MS^2 fragmentation scheme of **3** and proposed structure of identified key ion fragments (ion fragments in grey box are specific for **3**). RDA: retro-Diels Alder.

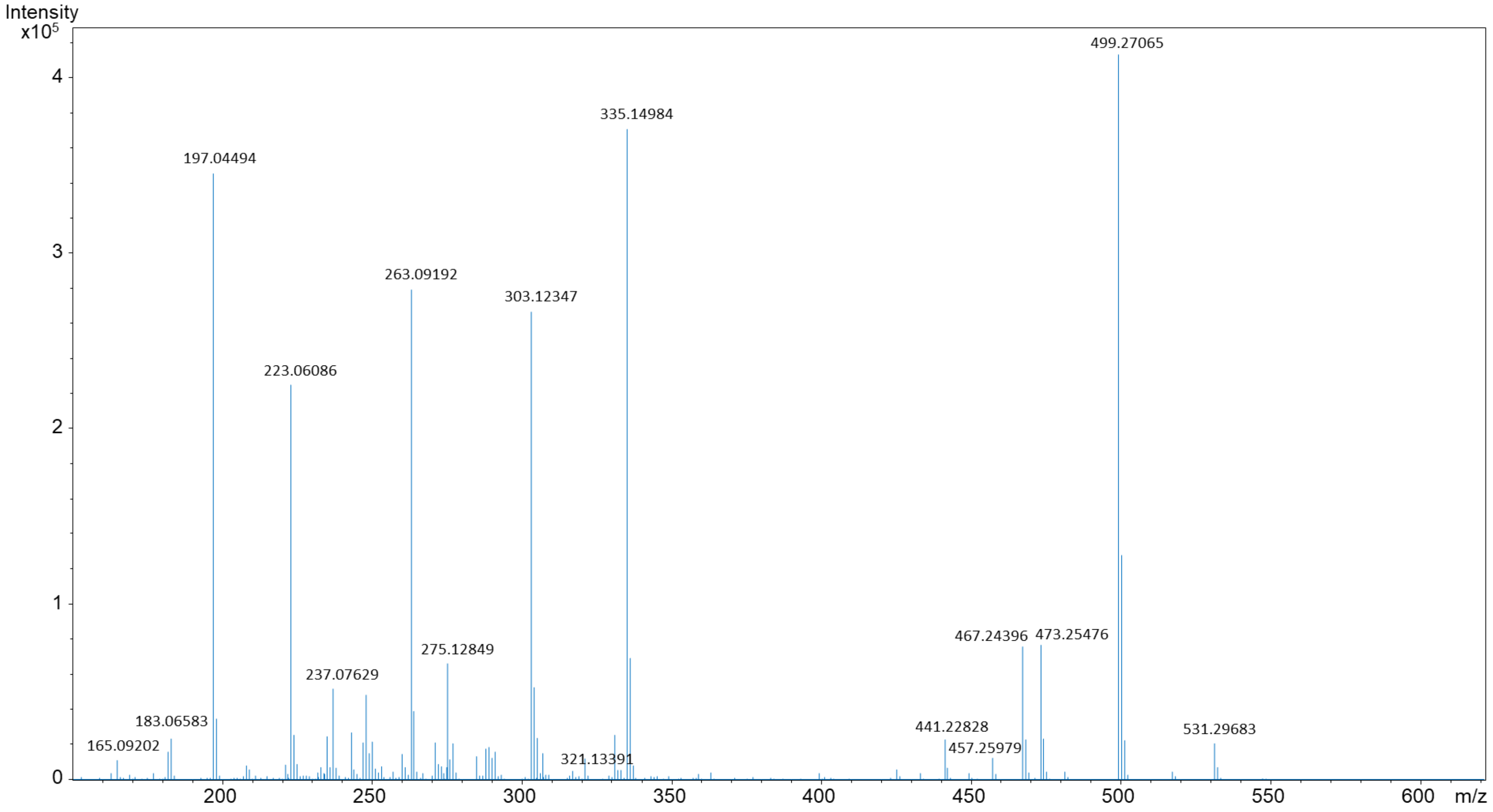

Figure S24. Fragmentation pattern in ESI+MS^2 experiment of stigmatellin C (isomer 1, **3**).

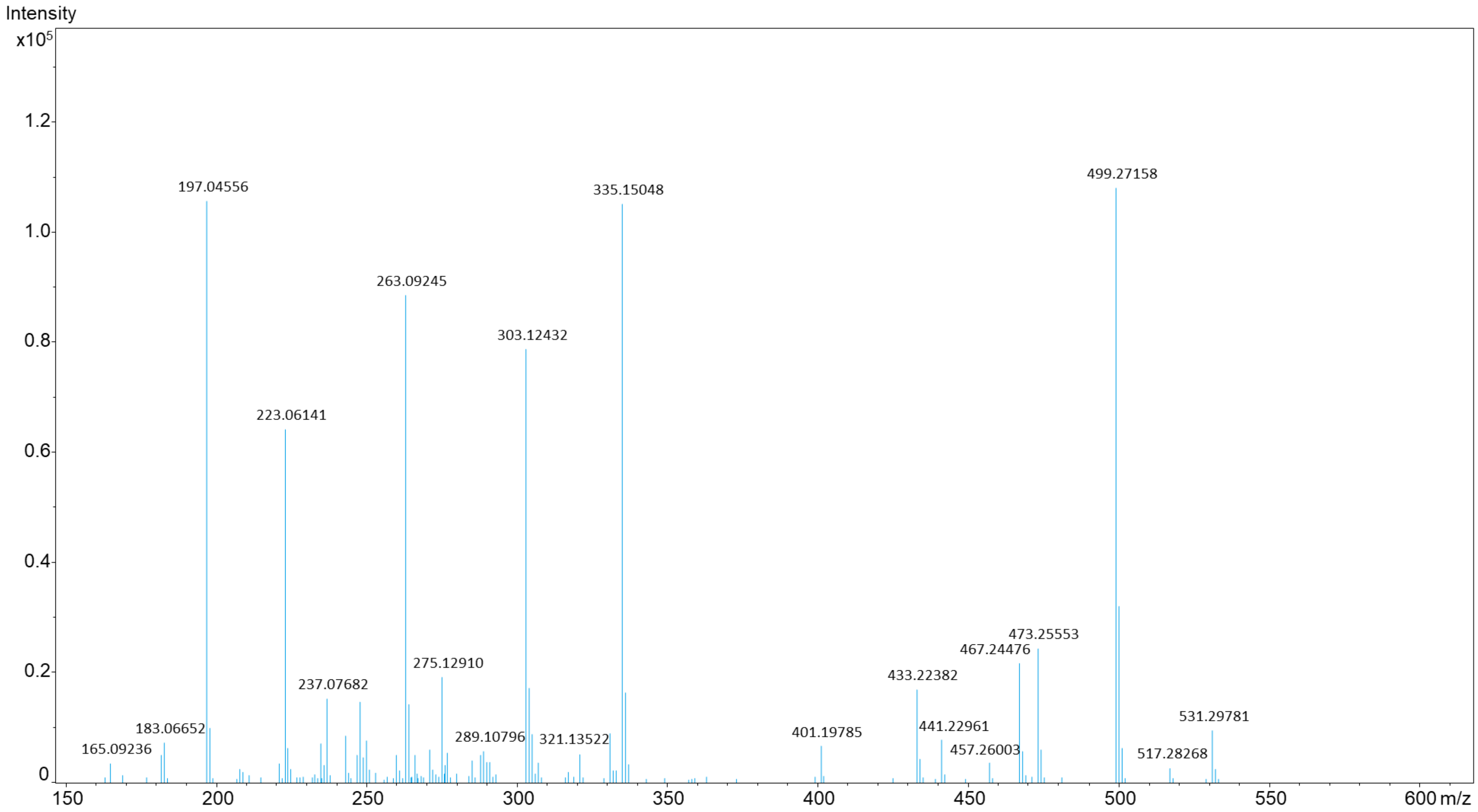

Figure S25. Fragmentation pattern in ESI+MS^2 experiment of stigmatellin C (isomer 2, **3**).

molecules

MDPI

Supplementary Materials 10

Table S6. Spectroscopic values for stigmatellin C (**3**) acquired in methanol-d_4 at 700 MHz.

Stigmatellin C (**3**)				
**Position**	**δ$_C$,**	**δ$_H$,** [m, *J* (Hz)]	**COSY**	**HMBC**
1a	148.1	-	-	-
2	165.5	-	-	-
3	117.3	-	-	-
4	179.7	-	-	-
4a	108.2	-	-	-
5	153.9	-	-	-
6	93.8	6.63 s 1H	-	4a, 5, 7, 8
7	152.6	-	-	-
8	129.6	-	-	-
9	10.0	1.98 s 3H	-	2, 3, 4, 1’, 2’
1’	30.5	2.84 m 1H<br>2.70 m 1H	H-2’	2, 3, 2’, 3’
2’	28.3	1.88 m 1H<br>1.55 m 1H	H-3’	1’, 3’,4’ 14’
3’	35.5	1.73 m 1H	H-2’,H-4’,H-14’	2’, 14’
4’	88.5	3.11 dd 1H (2.3/9.4)	H-4’,H-5’	2’, 3’, 4’-OCH_3, 5’, 6’,14’, 15’
5’	42.9	1.66 m 1H	H-4’’,H-6’,H-15’	4’, 15’
6’	82.4	3.88 dd 1H	H-5’,H-7’	4’, 5’, 7’, 8’, 15’, 6’-OCH_3
7’	133.4	5.60 dd 1H (7.4/15.2)	H-8’	5’, 6’, 8’, 9’
8’	133.8/133.9	6.20 dd 1H (10.5/15.3)	H-7’,H-9’	6’, 9’,10’
9’	129.6	6.31 ddd 1H (3.3/4.9/7.2/15.4)	H-10’	8’, 9’, 10’
10’	138.9/139.1	5.81 dd 1H (15.3/20.2)	H-9’	9’, 10’, 11’, 16’
11’	76.3/76.2	-	-	-
12’	74.9/74.8	3.56 s 1H (3.8/6.5)	H-13’	10’, 11’, 13’, 16’
13’	17.8	1.10 dd 3H (6.5/10.5)	-	11’, 12’, 16’
14’	18.2	1.14 d 3H (6.8)	H-3’	2’, 3’, 4’
15’	10.3	0.73 d 3H (7.1)	H-5’	4’, 5’
16’	23.2	1.24 d 3H (2.1/7.8)	-	9’, 10’, 11’, 12’
5O–	56.7	3.88 s 3H	-	5
7O–	56.9	4.00 s 3H	-	7
4’O–	61.7	3.47 s 3H	-	4’
6’O–	56.6	3.22 s 3H	-	6’

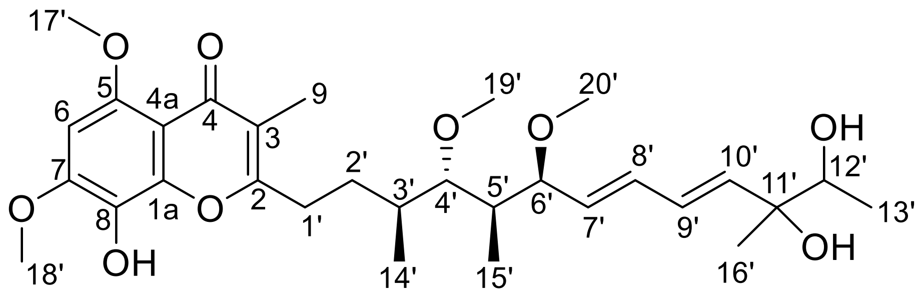

Stigmatellin C
3

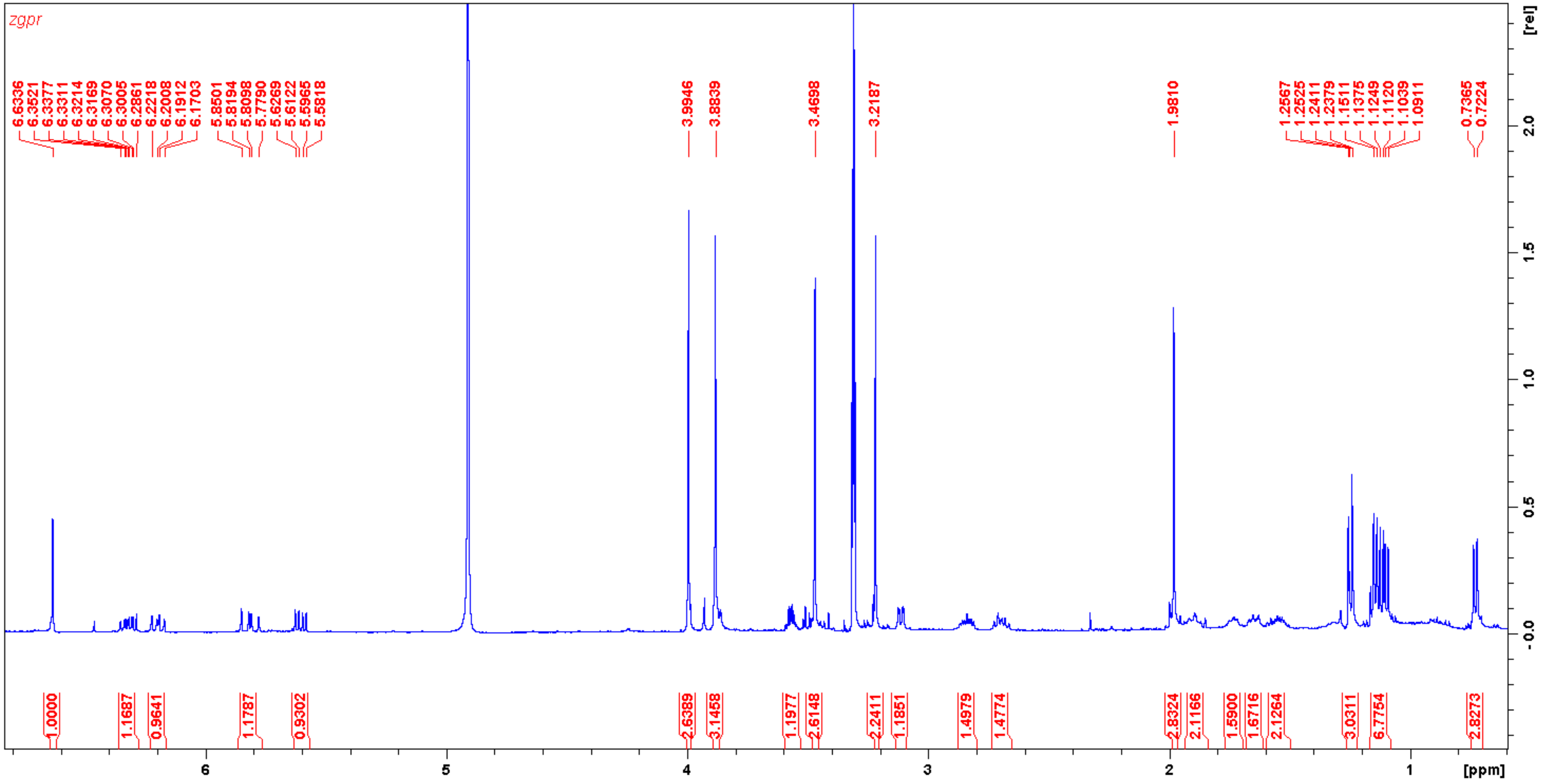

Figure S26. ^{1}H NMR spectrum stigmatellin C (**3**) in methanol-d_4.

molecules

MDPI

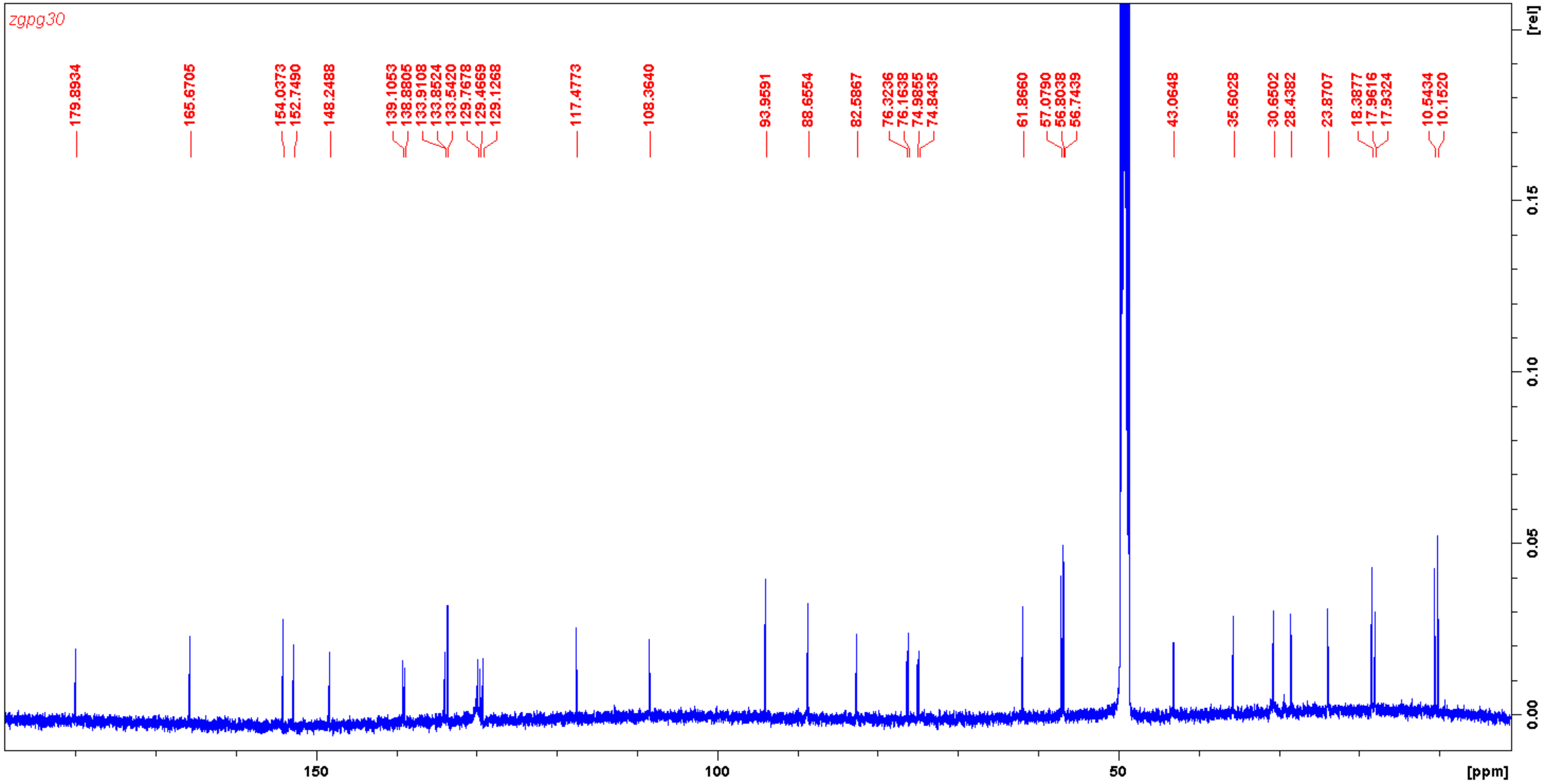

Figure S27. ^{13}C NMR spectrum of stigmatellin C (**3**) in methanol-d_4.

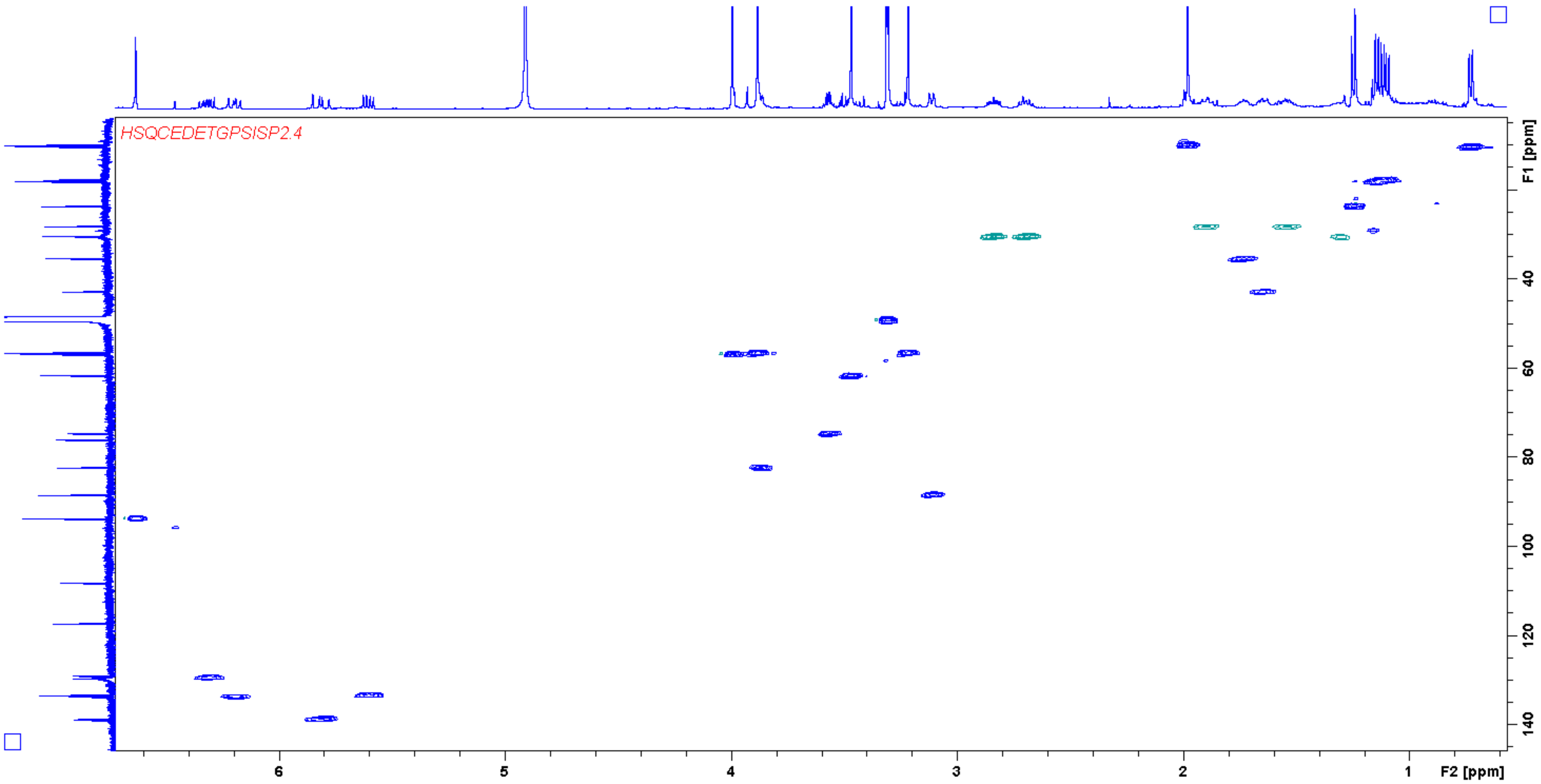

Figure S28. HSQC spectrum of stigmatellin C (**3**) in methanol-d4.

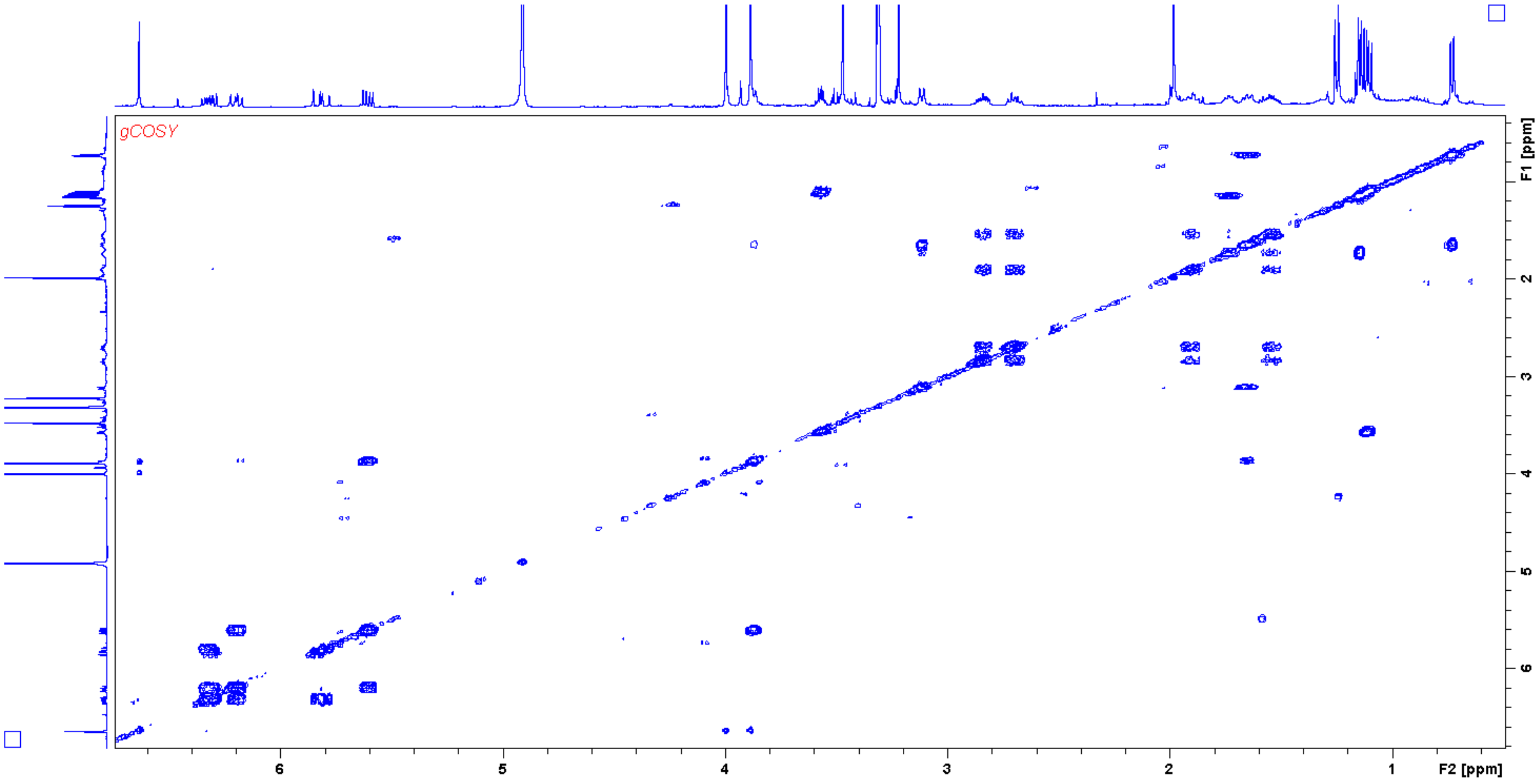

Figure S29. g-COSY spectrum of stigmatellin C (**3**) in methanol-d_4.

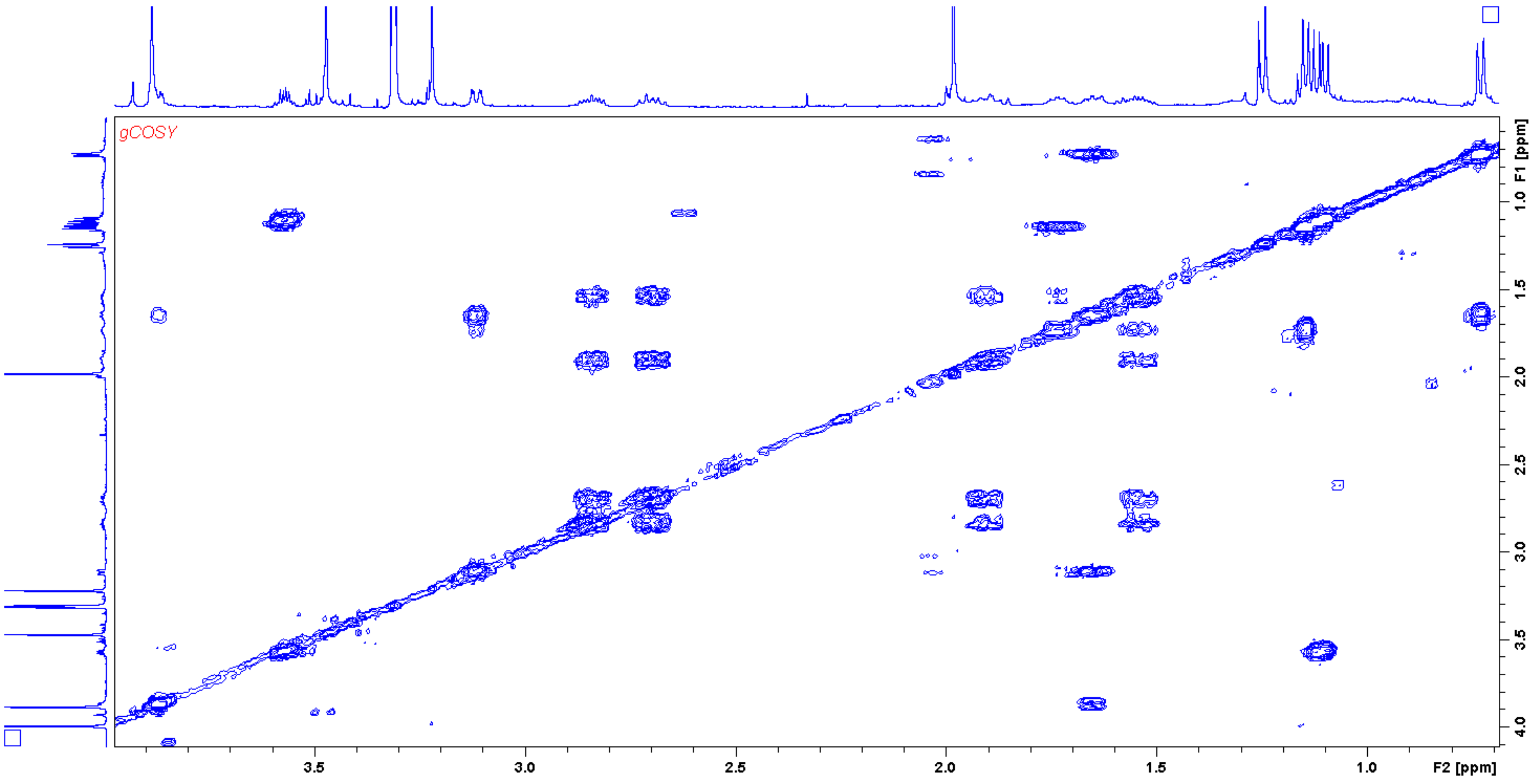

Figure S30. g-COSY spectrum (expanded) of stigmatellin C (**3**) in methanol-d_4.

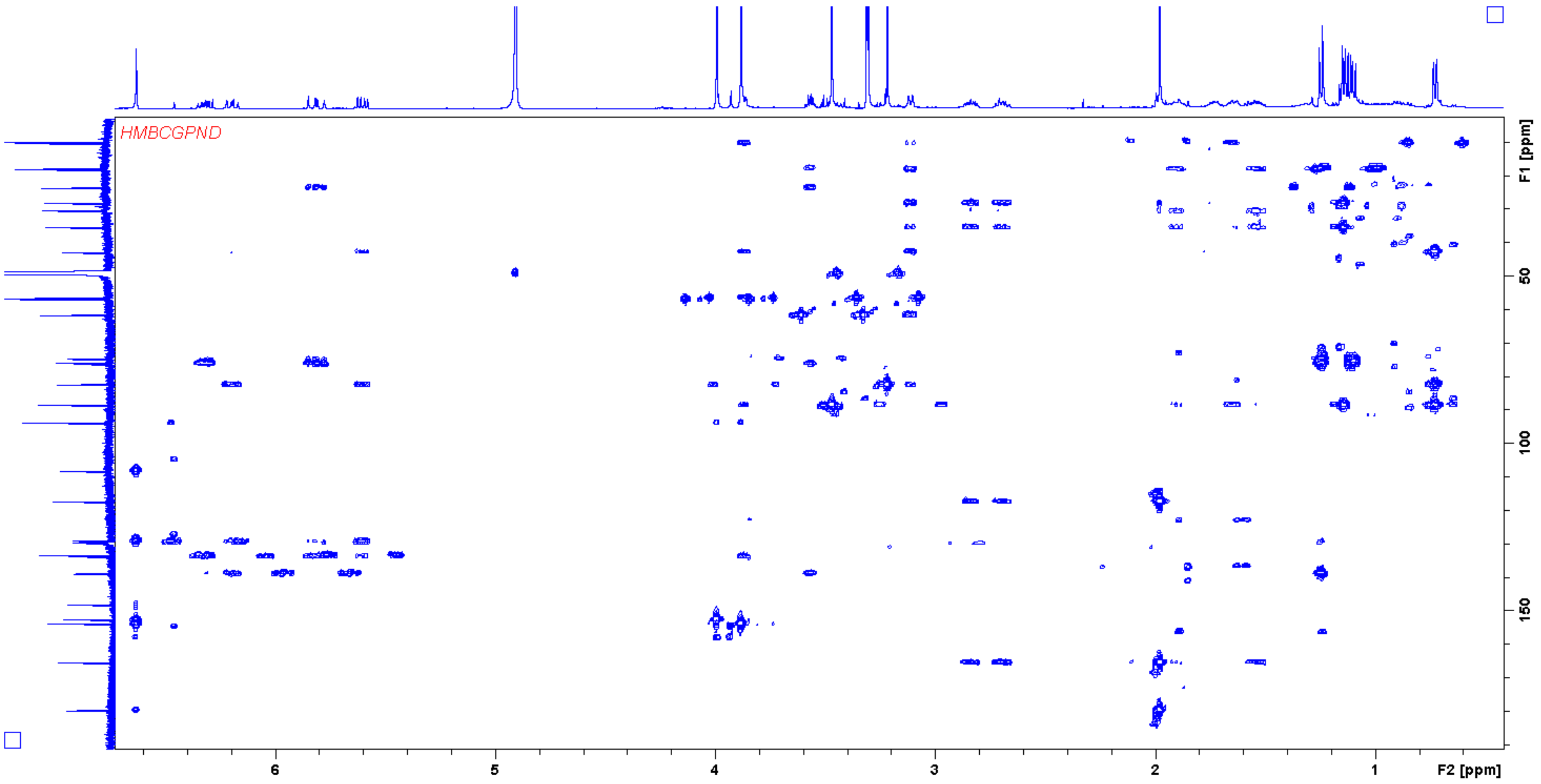

Figure S31. HMBC spectrum of stigmatellin C (**3**) in methanol-d_4.

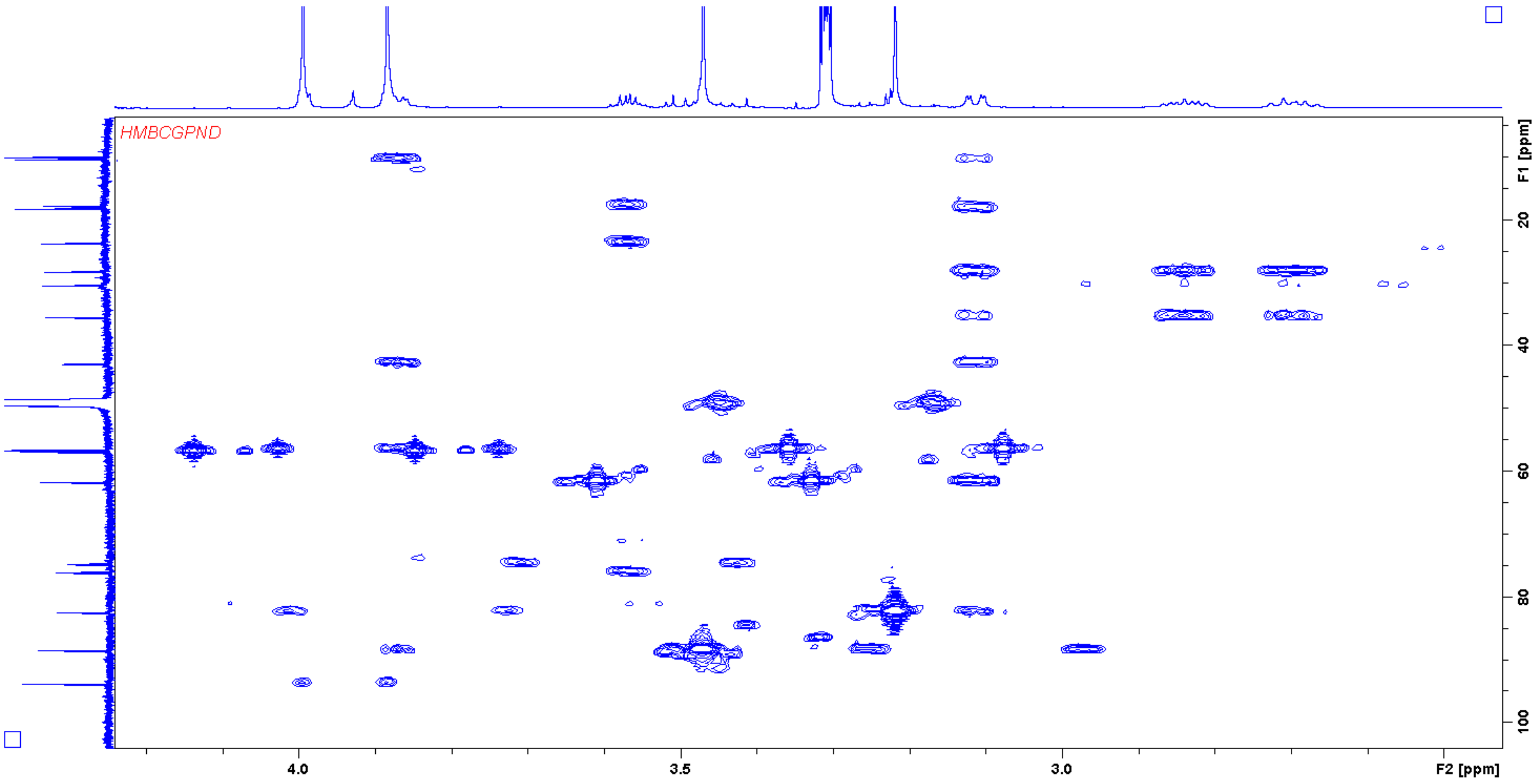

Figure S32. HMBC spectrum (expanded) of stigmatellin C (**3**) in methanol-d_4.

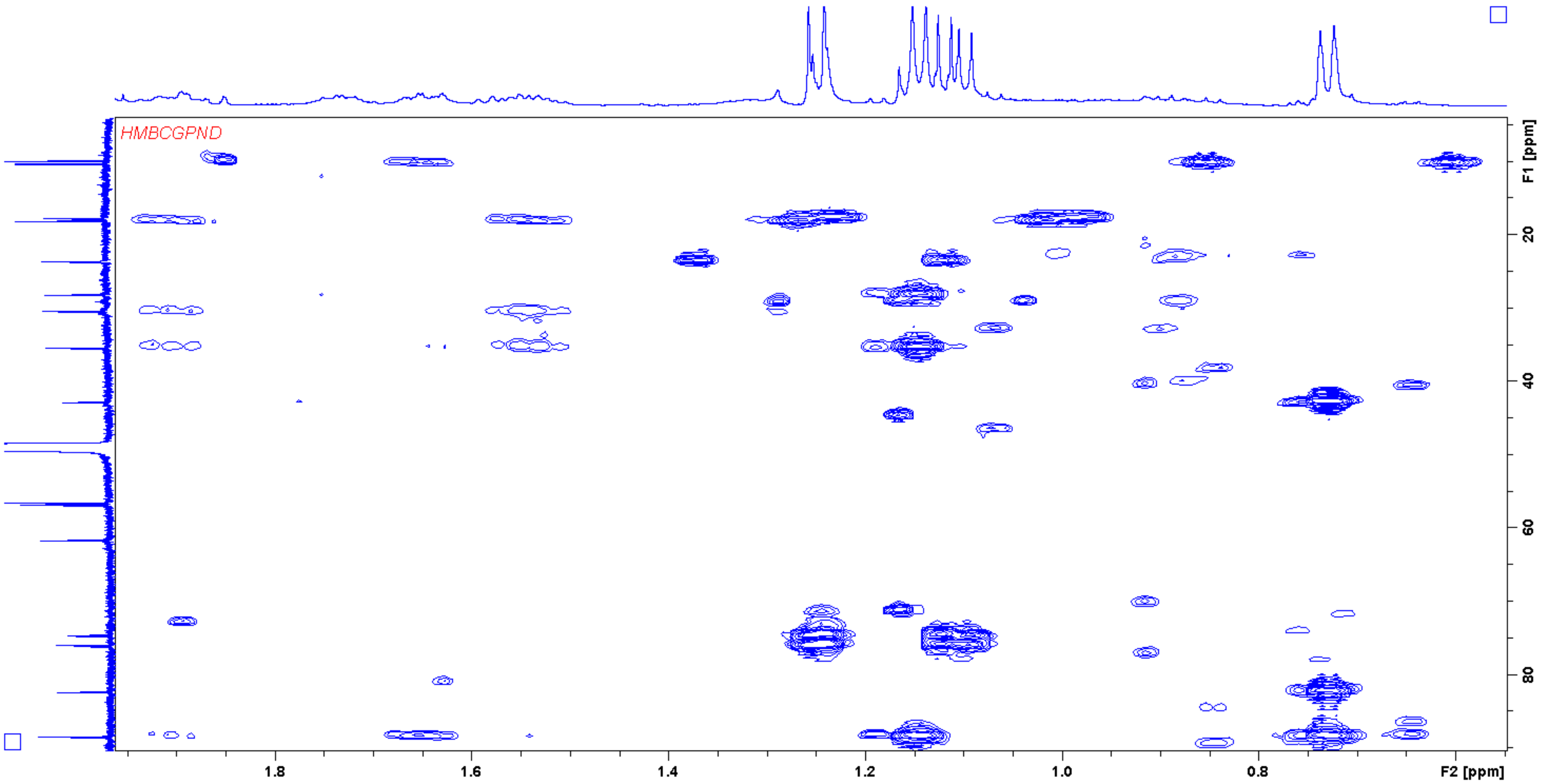

Figure S33. HMBC spectrum (expanded) of stigmatellin C (**3**) in methanol-d_4.

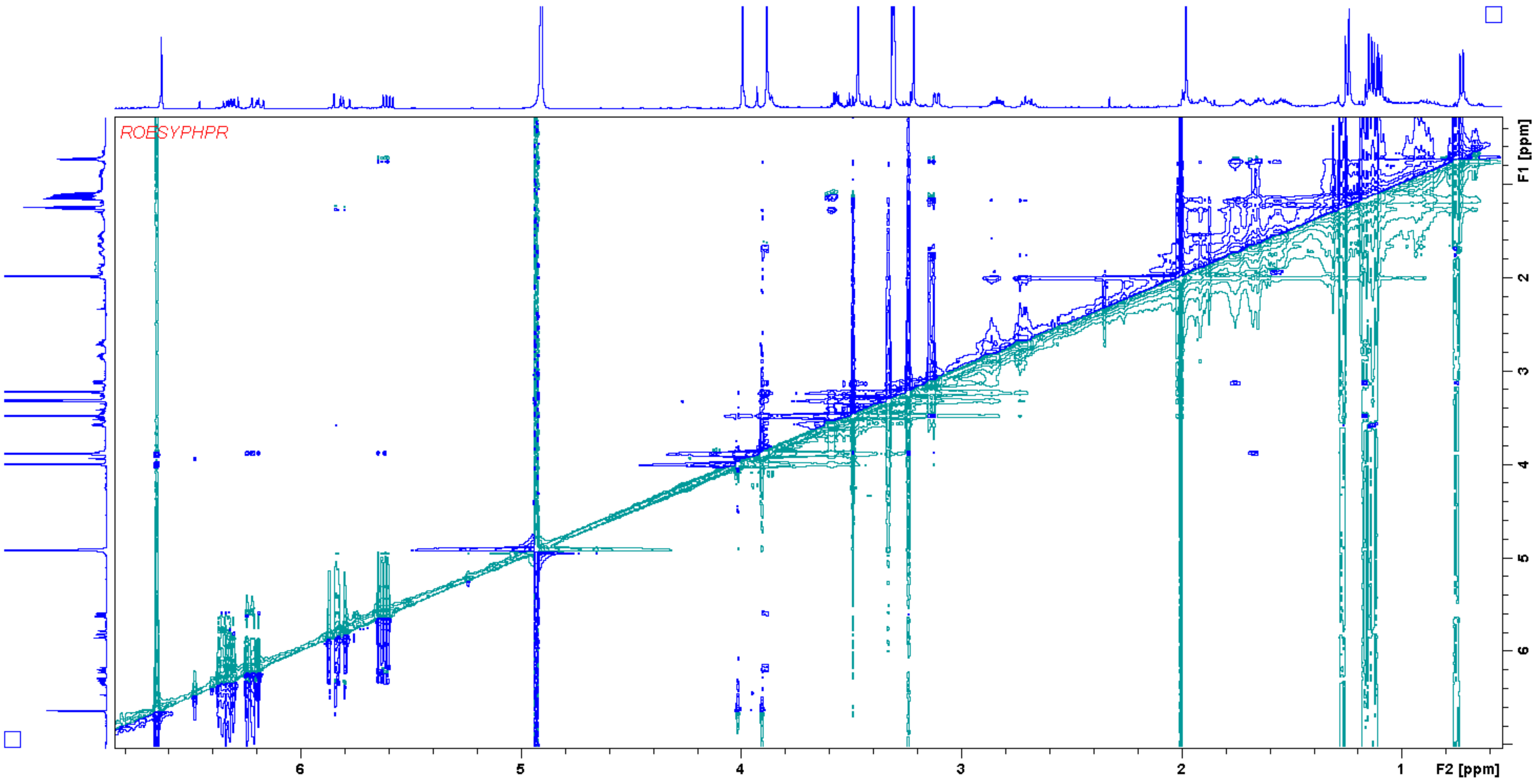

Figure S34. ROESY phpr spectrum of stigmatellin C (**3**) in methanol-d_4.

molecules

MDPI

Supplementary Materials 11

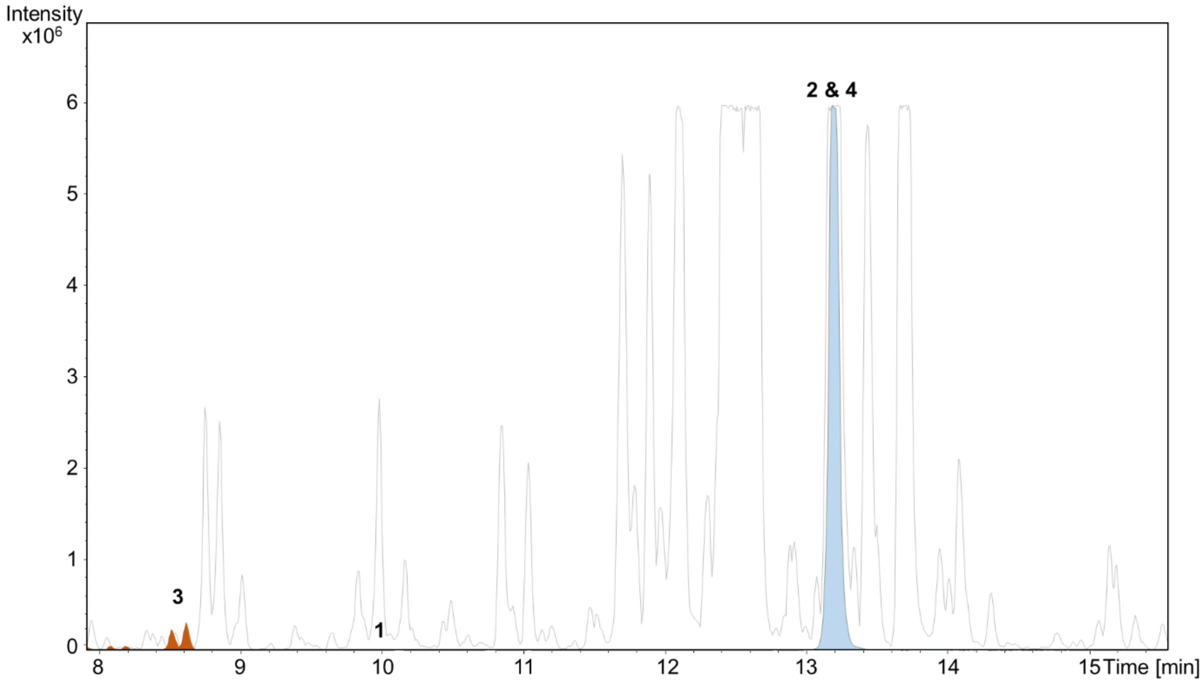

Figure S35. High performance liquid chromatography–mass spectrometry base peak chromatogram (HPLC–MS BPC) (grey) and extracted ion chromatograms (EICs) of **1** ($[M+H]^+$ 545.2755 m/z, green (not visible), **2** ($[M+H]^+$ 515.3004 m/z, blue), **3** ($[M+H]^+$ 549.3064 and 549.3060 m/z, brown), and **4** ($[M+H]^+$ 545.2755 m/z, blue) from *Stigmatella aurantiaca* Sg a15.

Supplementary Materials 12

Table S7. Predicted functions of the encoded proteins by the stigmatellin BGC from *Vitiosangium cumulatum* MCy10943^T.

Gene	Size (aa)	Deduced function	Closest homolog[1]	Coverage/ Identity (%)	Sg a15 homolog
orf1	308	DUF2381 family protein	WP_083682780.1	92/83	---
orf2	393	Serine/Threonine Protein Kinase	WP_204220004.1	99/92	---
orf3	134	Transcriptional regulator	WP_204220003.1	72/86	---
orf4	123	Transcriptional regulator	WP_204220002.1	100/89	---
orf5	458	HNH/ENDO VII superfamily nuclease	WP_204220001.1	93/93	---
orf6	203	Hypothetical protein	WP_203410745.1	96/75	---
orf7	122	Hypothetical protein	MBN1207532.1	82/84	---
orf8	1209	Helicase	WP_149184102.1	97/43	---
orf9	1097	Hypothetical protein	WP_230650848.1	97/32	---
orf10	388	Hypothetical protein	NQT93121.1	96/30	---
orf11	203	Hypothetical protein	WP_095987131.1	99/67	---
stiA	2364	PKS biosynthesis ACP-KS-AT-AT-DH-KR-ACP	AAK57190.1	99/49	99/79
stiB	1588	PKS biosynthesis KS-AT-KR-ACP	WP_203404417.1	97/53	100/82
stiC	1880	PKS biosynthesis KS-AT-DH-KR-ACP	WP_194142736.1	99/47	99/81
stiD	1932	PKS biosynthesis KS-AT-OMT-KR-ACP	WP_075010838.1	98/50	100/83
stiE	1925	PKS biosynthesis KS-AT-OMT-KR-ACP	WP_063796277.1	96/56	100/84
stiF	2212	PKS biosynthesis KS-AT-DH-ER-KR-ACP	BAZ29707.1	98/48	99/84
stiG	1402	PKS biosynthesis KS-AT-DH-ACP	NOT56266.1	87/51	100/81
stiH	1597	PKS biosynthesis KS-AT-DH-ACP	WP_196523766.1	95/48	99/79
stiJ1	1045	PKS biosynthesis KS-AT-ACP	MBD1836177.1	87/58	99/84
stiJ2	180	PKS biosynthesis	MBW4628907.1	99/28	99/78
stiK	256	PKS biosynthesis tailoring *O*-Methyltransferase	MBA2429587.1	100/51	100/85
stiL1	306	PKS biosynthesis tailoring Cytochrome P450 enzyme	WP_104977779.1	79/43	82/82
stiL2	230	PKS biosynthesis tailoring Cytochrome P450 enzyme	CTS25513.1	79/43	82/82
orf12	374	Transport	WP_108074850.1	100/95	---
orf13	911	Transport	WP_108074851.1	99/93	---
orf14	345	Transport	WP_108074852.1	95/88	---
orf15	530	Transport	WP_108074853.1	100/89	---
orf16	701	Glycogen debranching enzyme	MBC7855611.1	95/62	---
orf17	365	ATP-dependent 6-phosphofructokinase	WP_043407704.1	100/96	---
orf18	326	Glucokinase	WP_108074855.1	95/91	---
orf19	359	D-arabitol-phosphate dehydrogenase	WP_233262020.1	100/93	---
orf20	486	Pyruvate Kinase	WP_108074856.1	100/92	---
orf21	350	Hydroxyacid dehydrogenase	PTL78120.1	100/93	---
orf22	124	Hypothetical protein	WP_108074858.1	85/91	---

[1]For stiA–stiL2 the second closest homolog was denoted.

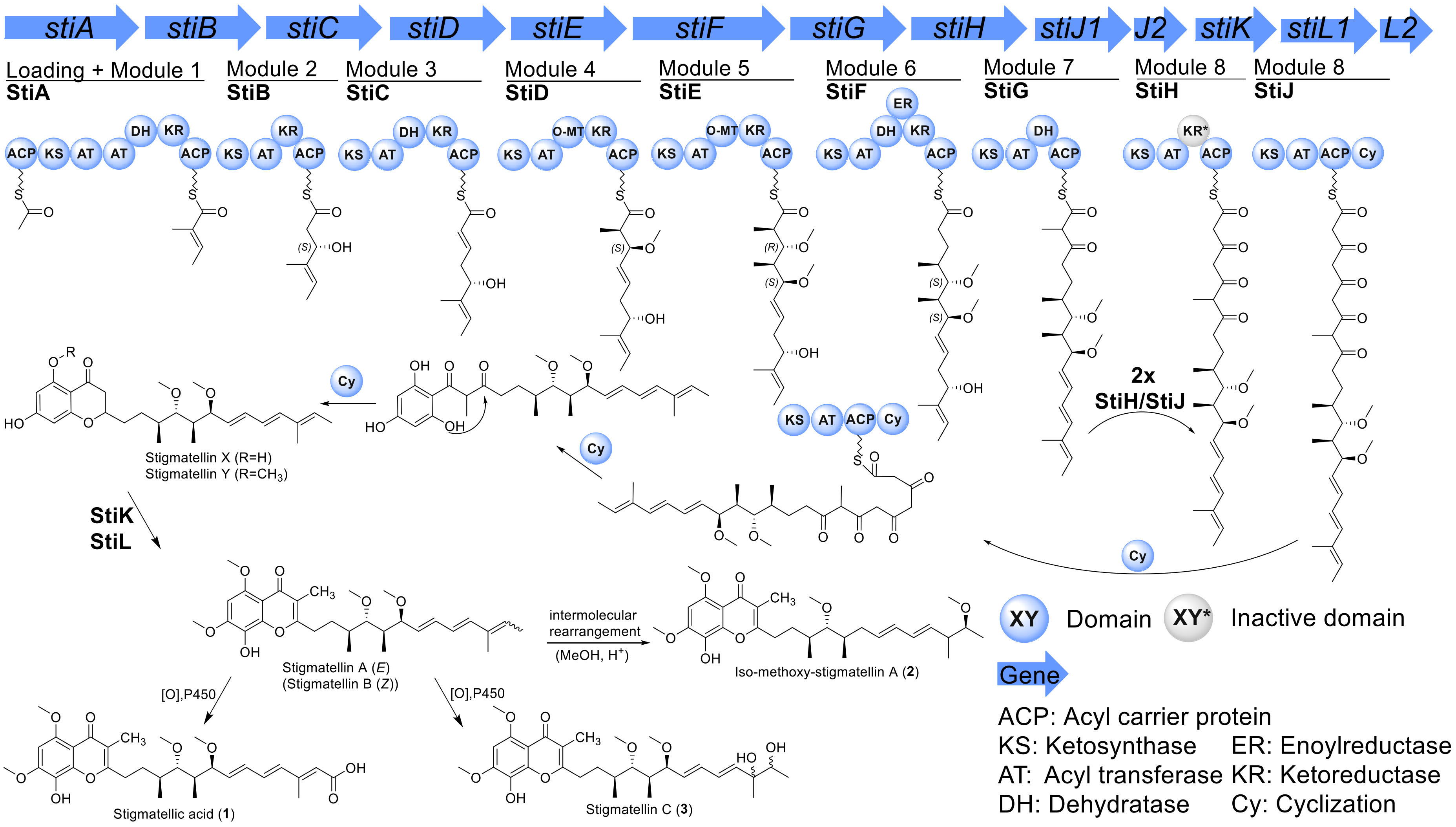

Figure S36. Genetic organization of the Stigmatellin BGC from *Vitiosangium cumulatum* MCy10943[T] and the proposed model of the biosynthesis leading to **1–3**. The stereochemical assignment of the linear biosynthetic intermediates bound to the assembly line was assigned based on the absolute configuration of **1–3**. Scheme adapted from Gaitatzis et al. [1] and Wenzel et al. [2]

molecules

MDPI

Supplementary Materials 13

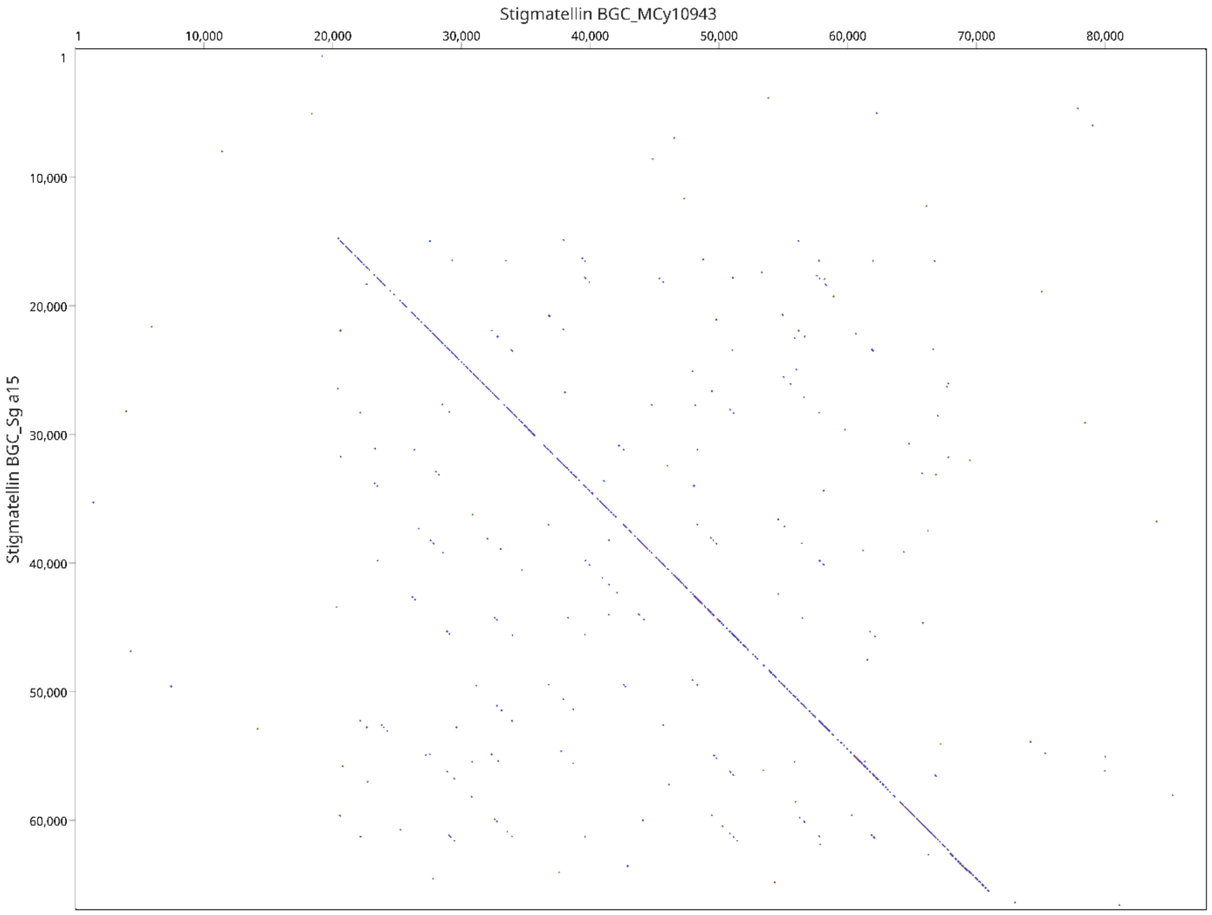

Figure S37. Dotplot of the entire herein identified stigmatellin BGC from *Vitiosangium cumulatum* MCy10943^{T} (GenBank: ON210143, 87854 bp) and the previously characterized BGC from Sg a15 (MIBiG: BGC0000153, 66808 bp). The blue diagonal line indicates the conserved genetic region responsible for the biosynthesis of stigmatellin in both producer strains (*stiA–stiL*), while the surrounding area is different and does not show similarity to each other (no visible lines indicate any similarity between *orf1–22* (MCy10943) and *orf1–8* (Sg a15).

2. References

1. Gaitatzis, N.; Silakowski, B.; Kunze, B.; Nordsiek, G.; Blöcker, H.; Höfle, G.; Müller, R. The biosynthesis of the aromatic myxobacterial electron transport inhibitor stigmatellin is directed by a novel type of modular polyketide synthase. *J. Biol. Chem.* **2002**, *277*, 13082–13090.

2. Wenzel, S.C.; Müller, R. Myxobacteria - unique microbial secondary metabolite factories. In *Comprehensive Natural Products Chemistry II, Vol 2: Structural Diversity II - Secondary Metabolite Sources, Evolution and Selected Molecular Structures*; Moore, B.S., Ed.; Elsevier: Oxford, 2010; pp 189–222.

3. Keatinge-Clay, A.T. A Tylosin Ketoreductase Reveals How Chirality is Determined in Polyketides. *Chem. Biol.* **2007**, *14*, 898–908.
